# TMD symptoms and vertical mandibular symmetry in young adult orthodontic patients in North Sumatra, Indonesia: a cross-sectional study

**DOI:** 10.12688/f1000research.14522.2

**Published:** 2018-07-16

**Authors:** Ervina Sofyanti, Trelia Boel, Benny Soegiharto, Elza I. Auerkari

**Affiliations:** 1 Doctoral Program, Faculty of Dentistry, Universitas Sumatera Utara, Medan, Indonesia; 2 Department of Orthodontics, Faculty of Dentistry, Universitas Sumatera Utara, Medan, Indonesia; 3 Department of Dentomaxillofacial Radiography, Faculty of Dentistry, Universitas Sumatera Utara, Medan, Indonesia; 4 Department of Orthodontics, Faculty of Dentistry, Universitas Indonesia, Jakarta, Indonesia; 5 Department of Oral Biology, Faculty of Dentistry, Universitas Indonesia, Jakarta, Indonesia

**Keywords:** vertical mandibular asymmetry; temporomandibular disorder

## Abstract

**Background:** Temporomandibular joint disorder (TMD) includes symptoms of pain and dysfunction in the muscles of mastication and the temporomandibular joint. Differences in vertical condylar height, observed in the assessment of mandibular asymmetry, is a structural alteration that represents a risk factor for TMD. The study aimed to evaluate the association between TMD symptoms and vertical mandibular symmetry in young adult orthodontic patients in North Sumatra, Indonesia.

**Methods:** The cross-sectional study included 18-25-year-old (mean ± SD, 21.9 ± 2.0 years) old orthodontic patients admitted to the Dental Hospital of Universitas Sumatera Utara, Medan, between June 2016 and March 2017. Vertical mandibular asymmetry was assessed from all 106 subjects using Kjellberg’s technique from pre-treatment panoramic radiographs. The TMD symptoms were assessed by structural interviews using modified questionnaires based on Temporomandibular Disorder Diagnostic Index and Fonseca’s Anamnestic Index.

**Results:** Of the 106 subjects, 26 (24.5% of the total) with vertical mandibular symmetry and 39 (36.8%) with vertical mandibular asymmetry were positive for TMD symptoms. By contrast, 17 patients (16.0% of the total) with vertical condylar symmetry and 24 patients (22.6%) with vertical mandibular asymmetry were regarded negative for TMD symptoms. There was no significant difference (p=0.520) in TMD symptoms based on vertical mandibular symmetry.

**Conclusion:** The results from this studied Sumatran population indicate that there are common TMD symptoms in young adult orthodontic patients, but there is no significant association between vertical mandibular asymmetry and TMD symptoms. Further study on the development of TMD, mandibular asymmetry and treatment planning for growing patients is suggested, using longitudinal and transitional approaches.

## Introduction

The goal of orthodontics for young patients is to provide a functional occlusion to give harmony in the dental arrangement, the anatomy of temporomandibular joints, and the activity of the masticatory muscles in later adulthood ^1^. The assessment of symmetry is important in comprehensive orthodontic treatment, as well as in malocclusions and dental evaluation; it is related to this aforementioned goal of orthodontic treatment, especially the functional and aesthetic evaluation of the craniofacial region ^2, 3^. The asymmetries in human facial structures affect the skeleton, muscles and corresponding attached facial tissues. The prevalence of mandibular asymmetry is highest when compared to asymmetry of cranial base and maxillary arch in the human skull ^4^. In orthodontic assessment, it is also important to consider whether the development of mandibular asymmetry could affect jaw, head and even shoulder movement, creating problems that should not occur in healthy subjects ^5– 7^.

According to a study on 8–30-year-old subjects in Jakarta, Indonesia, based on questionnaires and posteroanterior radiography, the main risk factor of mandibular asymmetry is temporomandibular joint disorder (TMD) ^8^. Asymmetry in the vertical dimension, based on posteroanterior radiography, was significantly correlated with temporomandibular joint internal derangement in a study on 187 Japanese subjects with pre-orthodontic mandibular asymmetry and a mean age of 23.9 years ^9^.

The etiology, diagnosis and management of mandibular asymmetries focuses particularly on developmental asymmetries. Increasing mandibular asymmetry with bilateral asymmetry of morphological traits causes malfunctions in developmental homeostasis, associated with environmental and/or genetic stresses. The development of morphological asymmetry may serve as a risk factor for disorders of developmental origin if these stresses are involved. The biopsychosocial model is hypothesized to be the most accepted theory for developmental asymmetry and complexity of TMD ^5, 10^. Studies concerning TMD and the relevance of orthodontic treatment suggest that the achievement of a balanced in the dynamic occlusion is necessarily related to the development of mandibular symmetry as a part of the successful management of TMD ^2, 10– 13^. 

Panoramic radiography is commonly used to assess the extent of mandibular asymmetry, as bilateral information is provided in routine dental practice. The asymmetry indices of mandibular height based on the ratio of condylar height (CH) and ramus height (RH) asymmetry, according to Habets’ method and Kjellberg’s technique, correlated significantly between TMD and non-TMD patients ^14, 15^. However, Kjellberg’s technique is easier in terms of identifying the points and measurements and compares both sides because the measurement of CH from the highest point of condylar head to the mandibular notch; this differs from Habet’s method, which uses the distance from the highest point of the condyle to the most lateral point of the condyle ^16^. A previous study of 100 patients with TMD in the Seoul National University Dental Hospital between 2009 and 2011 found that asymmetry resulting in more than a 4.37% difference between mandibular heights may increase the risk of TMD and was positively correlated with the incidence of arthritic change in the temporomandibular joint of patients with TMD, although this does not necessarily indicate a direct cause-and-effect relationship ^7^. By contrast, there was no statistically significant difference found between the severity of signs and symptoms of TMD based on vertical mandibular asymmetry, assessed using Habets' method and Kjellberg's techniques in 12–65-year-old patients ^17^. Since TMD and mandibular asymmetry are complex issues that cover a large variety of symptoms, this study aims to analyze the association between TMD symptoms and vertical mandibular asymmetry measured using Kjellberg’s technique in young adults that sought orthodontics treatment at the Dental Hospital in Universitas Sumatera Utara, Medan, Indonesia.

## Methods

This cross-sectional study was conducted at the Dental Hospital of the Faculty of Dentistry, Universitas Sumatera Utara between June 2016 and March 2017. The Health Research Ethical Committee of the Medical Faculty, Universitas Sumatera Utara (100/DATE/KEPK FK USU-RSUP HAM/2017) approved the study. All 106 subjects were 18–25-year-old patients that attended the orthodontic clinic for a consultation, with following eligibility criteria: no previous orthodontic treatment, no history of traumatic facial injury or congenital disease. Patients who attended the orthodontic clinic had been informed that if they provided written informed consent, they would be included in a survey. In compliance with the Declaration of Helsinki, the consenting participants were asked to fill in the questionnaire on the Temporomandibular Disorder Diagnostics Index (TMD-DI; Table 1) at the Orthodontics clinic, Dental hospital Faculty of Dentistry, Universitas Sumatera Utara. The assessment of TMD symptoms were based on TMD-DI with categories of TMD-positive or TMD-negative ^18^. The assessment of stress ( Table 2) was using questions for a modified Fonseca’s Anamnestic Index, related to bruxism, joint noise and nervousness ^19^.

**Table 1.** Temporomandibular Disorder-Diagnostic Index (TMD-DI).

Number	Questions	Code	Filling instructions
1	Do you have headache?		Fill in code with 0=never 1=sometimes 2=often 3=always
2	Do you feel pain when closing and opening mouth?		
3	Do you have joint trismus when getting up in the morning?		
4	Do you feel pain around neck?		
5	Do you have tinnitus?		
6	Do you clench your teeth when worried?		
7	Do you clench your teeth when in anger?		
8	Do you clench your teeth when concentrating?		
*Total score* Total score: 0–24 Total score ≤3: TMD symptom code = 0 (TMD negative) Total score >3: TMD code = 1 (TMD positive)			

**Table 2.** The modified Fonseca’s Anamnestic Index.

Number	Questions	Code	Filling instructions
1	Have you noticed noise in your temporomandibular joints while chewing or opening your mouth?		Fill in code with 0=never 1=sometimes 2=often 3=always
2	Do you have habits of clenching or grinding your teeth?		
3	On whether you consider yourself a tense (nervous) person, please answer the following questions:		
	A. Do you sweat excessively (e.g. sweaty hands) even when it is not hot, or without physical activity? *		
	B. Do you feel changes in cardiac activity even without physical activity (e.g. increased or weakened heart rate)? *		
	C. Will you become easily angry because of trivial things? *		
	D. Will you become impatient when experiencing delays (e.g. in traffic jams or when waiting for something)? *		

*Modification to questionnaires to assess stress.

**Figure 1.**
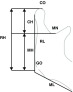
Landmark points of vertical mandibular symmetry according to Kjellberg’s technique.

**Figure 2.**
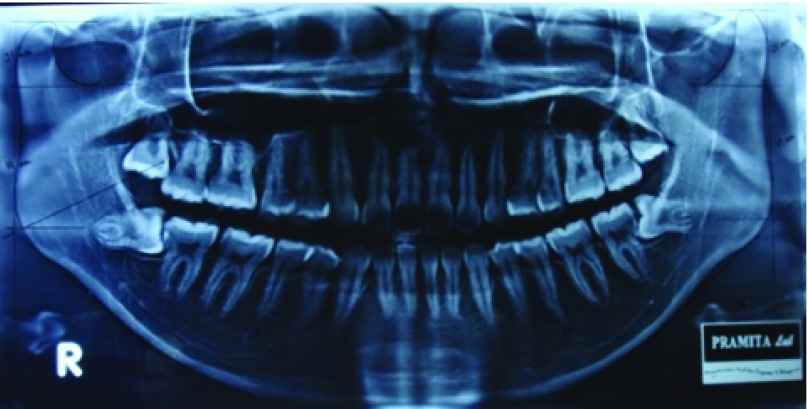
The measurement of panoramic radiograph that used in this study.

Subjects were referred to take panoramic radiography with exposure parameters (80 kV, 15 mA, 12 seconds) in the Pramita Clinic and laboratory, Medan, North Sumatera, Indonesia. Figure 1 shows the classification of vertical mandibular height symmetry. A percentage symmetry of 93.7% or lower was defined as vertical mandibular asymmetry based on Kjellberg’s technique ^17^. Figure 2 showed the measurement of vertical mandibular symmetry manually on tracing paper. Figure 1 showed the points in measuring the vertical mandibular symmetry as follows: CH is defined as the distance from CO (the highest point of the condylar head) to the mandibular notch (the deepest point between the coronoid process and the condylar process). RH is the distance from CO to the Go’ (the reflection of subdivision tangen from ramus and corpus mandibular to the ramus borderline). In order to obtain vertical mandibular symmetry based on the ratio of condylar and ramus height (Kjellberg's technique), the numerator should be smaller than the value resulting from the division of CH and RH/MH regardless of whether it corresponds to the right or left joint. The formula is as follows:

                                                                                                                         Kjellberg symmetry index = $\frac{\left(\frac{\mathrm{CH}}{{\mathrm{RH}}_{A}}\right)}{\left(\frac{\mathrm{CH}}{{\mathrm{RH}}_{B}}\right)}$

To determine the random error, inter-rater (T.B. and E.S.) and intra-rater (E.S. under supervised of T.B.) measurements of variables in this study were randomly done from 20 panoramic radiographs. The validity and reliability, measured using Cohen’s κ, showed moderate agreement for inter-rater measure-ments between T.B and E.S (κ=0.538) whilst intra-rater measurements from E.S that repeated the measurements 1 week after the first examination and blinded to the initial values (κ=0.674). Finally, this study used intra-rater measurement as reference data for assessing vertical mandibular symmetry. Cronbach’s alpha analysis was used to provide reliability measurements of questionnaires in analysing items and total scores in the modified Fonseca’s Anamnestic Index (p>0.05). However, the point related to clenching or grinding was omitted as it failed to show the validity and reliability of criteria (p=0.023). Then any information regarding FAI was only collected for additional information and the TMD-DI used as early screening for analyzing the TMD symptom ^20, 21^. Significance of association between TMD symptoms and vertical mandibular symmetry (or asymmetry) was evaluated using a chi-squared test, with assumed significance at p < 0.05. All statistical analyses were performed using SPSS, version 18.0 (SPSS, Inc., Chicago, IL, USA).

## Results

From 106 young adult orthodontic patients (mean ± SD, 21.9 ± 2.0 years old), TMD symptoms were present in 24.53% (n=26) of patients with vertical mandibular symmetry and in 36.79% (n=39) with vertical mandibular asymmetry. On the other hand, 16.04% (n=17) of patients with vertical mandibular symmetry and 22.64% (n=24) with vertical mandibular asymmetry had no TMD symptoms ( Table 3). There was no significant difference (p=0.520) in the occurrence of TMD symptoms based on vertical mandibular symmetry ( Table 3).

**Table 3.** The difference in temporomandibular joint disorder (TMD) symptoms based on vertical mandibular symmetry.

Vertical mandibular symmetry ≤93.7%	TMD		P-value
	Negative	Positive	
Symmetry	17	26	0.520
	16.04%	24.53%	
Asymmetry	24	39	
	22.64%	36.79%	

All radiographic images taken of the patients Answers to the original Indonesian language questionnaire are also present. Click here for additional data file. Copyright: © 2018 Sofyanti E et al. 2018 Data associated with the article are available under the terms of the Creative Commons Zero "No rights reserved" data waiver (CC0 1.0 Public domain dedication).

Vertical mandibular symmetry measurements using Kjellberg’s technique, alongside responses to each questionnaire A key is present in the “Questionnaires” sheet. Click here for additional data file. Copyright: © 2018 Sofyanti E et al. 2018 Data associated with the article are available under the terms of the Creative Commons Zero "No rights reserved" data waiver (CC0 1.0 Public domain dedication).

## Discussion

The most frequent TMD symptoms include joint noises, followed by reduced mandibular mobility, muscular pain and joint pain. TMJ status is an important factor to consider in orthodontic diagnosis because related to imbalance occlusion and the development of mandibular asymmetry ^3, 7^. A previous study suggested that MRI or arthrography could be used as a valuable radiographic assessment in analyzing condylar hyperplasia or discus displacement in mandibular asymmetry and TMJ ^22^. The assessment of posteroanterior cephalometric variables could be used as a key factor for evaluating the presence of unilateral TMD ^23^. TMD signs and symptoms with multifactorial etiologies have been reported as a risk factor in patients with mandibular asymmetry that had menton deviations in Indonesia based on postero-anterior radiography ^8, 20^. In the early detection of mandibular asymmetry related to TMJ disharmony, panoramic radiography is routinely used in the clinic for orthodontic purposes, compared to bilateral tomography of TMJ and postero-anterior radiography. This technique allows a bilateral view and adequate information on vertical and horizontal measurements as early diagnostic evaluation of mandibular asymmetry because it focus mainly on intercondylar asymmetries and gonial angle measurements ^24– 28^. Previous studies about panoramic radiography reported that horizontal measurements of anatomic landmarks in the panoramic radiograph tend to be particularly unreliable because of the nonlinear variation in magnification at different object depths, whereas vertical and angular measurements are acceptable, provided the patient’s head is positioned properly ^24, 25, 28, 29^. This study used Kjellberg’s technique because it is easier to identify the condylar height using this technique than Habets’ method in vertical mandibular symmetry assessment. Habets’ method is more complicated when making reference points of the most lateral point of the condyle due to variation in the condylar anatomy ^16, 17, 29^.

Early detection of TMD in malocclusion, especially related to mandibular asymmetry before establishing orthodontic therapy, is mandatory for interdisciplinary approaches for any dentofacial treatment nowadays ^2, 11, 30, 31^. Some questionnaires can be used as a tool to achieve early detection of TMD. Fonseca’s Anamnestic Index has frequently been used to classify individuals according to TMD severity category, from no TMD to mild, moderate and severe TMD, to screen TMDs in Brazilian women with regards to anxiety as a stress factor ^19^. The TMD-DI was developed by Himawan *et al*. in 2006, has been applied in the study the characteristics of TMD and other risk factors in the Indonesian society ^8, 18, 20, 21^. In our study, we modify the questions regarding anxiety as stress factor to detect the severity of TMD symptom. In validity and reliability analysis, There was a question regarding clenching and grinding habit was eliminated due to no significant difference in validity and reliability analysis, so this study executed to analyze the TMD symptom based on FAI data. Eventhough this study only used the TMD-DI, there were a higher prevalence of TMDs in both of symmetry and asymmetry vertical mandibular of these young adult orthodontic patients (mean age ± SD, 21.9 ± 2.0 years old). Based on the aforementioned goal of orthodontic treatment, the clinician should be aware of TMD symptoms in orthodontic treatment related to functional efficiency. However, the differences in pain threshold might be a distraction factor while answering the questions to assess TMD symptom. Then, the proper clinical examination of the temporomandibular joint should be considered in orthodontic patients.

Fundamentally, orthodontic treatment should create a balanced and stomatognathic system, especially the temporomandibular joint. One element of this balance is craniofacial symmetry, which is frequently subject to discussion between clinicians and is the subject of multiple different studies in the last decades ^2, 11, 12^. Although perfect craniofacial symmetry does not exist in nature, gross abnormalities in symmetry are considered as a major cause of non-dental pain in the orofacial pain region ^10, 12, 32^. The distribution of TMD symptoms is higher than that of non-TMD symptoms in orthodontic patients with and without vertical mandibular symmetry. However, there was no significant difference (p=0.502) in TMD symptoms based on the presence or absence of vertical mandibular symmetry ( Table 3) since TMD the etiology of TMD is multidimensional ^33^ and asymmetry of condylar width, height and length as common features in TMD based on 3D-computed tomography ^34, 35^. Indonesia, as a developing country, still uses panoramic digital radiography as initial evidence for planning early orthodontic intervention and avoiding the progression of asymmetries ^36^.

According to McNamara, orthodontic treatment performed during adolescence does not alter TMD risk, as TMD with mandibular asymmetry may increase with age, with no evidence originating during orthodontic treatment ^31^. This condition is due to the asymmetrical function and activity of the jaws, and the different development of the right and left sides of the mandible. The morphology of the condyle on the deviated side differs from the non-deviated side in mandibular asymmetry, indicating the association between asymmetrical jaw function and joint remodeling, based on 3D-cone beam computerized tomography ^34, 35^. The present cross-sectional study concerning mandibular symmetry and TMD in young adult orthodontic patients in North Sumatra indicates that asymmetry has been an adaptive response to functional demands because the mandible adapts to mandibular deviations. The modelling of condyle and glenoid fossae, as well as higher appositional growth in the gonion region during jaw function, will influence skeletal and dental pattern in later adulthood.

It is vital for any clinician who is involved in altering the patient’s dentofacial appearance and stomatognatic function to consider the mandibular symmetry and TMJ function, whether through orthodontics, facial growth modification, corrective jaw surgery or any aesthetic dentistry. In the future, although the result in Table 3 showed a non-significant correlation, the TMD-DI as early screening for TMD might require panoramic radiography with postero-anterior radiography or 3D-cone beam computerized tomography to analyze the complexity of TMD development and mandibular asymmetry. In this study, the orthodontic patients presented with complex stomatognati problems, such as missing posterior teeth which regardless the missing duration. This condition could affect the development of TMD symptoms and vertical mandibular asymmetry; this matches the study by Halicioglu *et al.*, which reported a slight difference in the vertical mandibular symmetry index was found in patients with early unilateral mandibular first molar extractions ^37^.

Mandibular asymmetry and TMD are two common features associated with increased bilateral asymmetry in morphological traits which might involve environmental and/or genetic stresses as etiologies in breakdown in developmental homeostasis. The etiopathogenesis of TMD, which is a common feature in mandibular asymmetry, is poorly understood, because the complexity of biomechanical, neuromuscular, bio-psychosocial and biological factors has contributed to this disorder ^6, 33^. Clinicians should note that the complexity of dentofacial variation in orthodontic patients indicates in part why most treatment approaches for malocclusions with TMD are directed to the symptoms rather than to etiology. However, a combination of questionnaires (as diagnostic indexes) and radiography analysis of predominantly vertical or horizontal mandibular asymmetries indicates that susceptibility to fluctuating asymmetry is increasing ^32^. In the future, some translational approaches with the identification of molecular regulators of cell proliferation in the condylar cartilage, coupled with these phenomena, might carry this finding into the clinical setting. Expanding the fields of phenomics and genomic medicine to understand why asymmetric function occurs is required to achieve personalized orthodontic treatment in young orthodontic patients ^38^. Stress might also have a role in the appearance of developmental disorders and required comprehensive diagnostic tools ^11, 19, 33^.

## Conclusions

TMD symptoms appear common in the studied young adult orthodontic patients from North Sumatra, but no significant association was observed between vertical mandibular asymmetry and symptoms of TMD. Further studies on the development of TMD, mandibular asymmetry and treatment planning for growing patients are suggested, using longitudinal and transitional approaches.

## Data availability

The data referenced by this article are under copyright with the following copyright statement: Copyright: © 2018 Sofyanti E et al.

Data associated with the article are available under the terms of the Creative Commons Zero "No rights reserved" data waiver (CC0 1.0 Public domain dedication). 

**Dataset 1. All radiographic images taken of the patients.** Answers to the original Indonesian language questionnaire are also present. DOI: 10.5256/f1000research.14522.d205359 ^39^.

**Dataset 2. Vertical mandibular symmetry measurements using Kjellberg’s technique, alongside responses to each questionnaire.** A key is present in the “Questionnaires” sheet. DOI: 10.5256/f1000research.14522.d205360 ^40^.
