# Supplementary material for: TMD symptoms and vertical mandibular symmetry in young adult orthodontic patients in North Sumatra, Indonesia: a cross-sectional study
Source: F1000Res. 2018 Jul 16;7:697. Originally published 2018 Jun 4. [Version 2] doi: 10.12688/f1000research.14522.2 (PMC5998004; doi:10.12688/f1000research.14522.2)
Supplement: All radiographic images taken of the patients — Answers to the original Indonesian language questionnaire are also present. [file f1000research-7-16886-s0000.tgz › 8e66cae7-dfdb-4212-acee-ec1f321623b7_Dataset_1.pptx]

## Slide 1
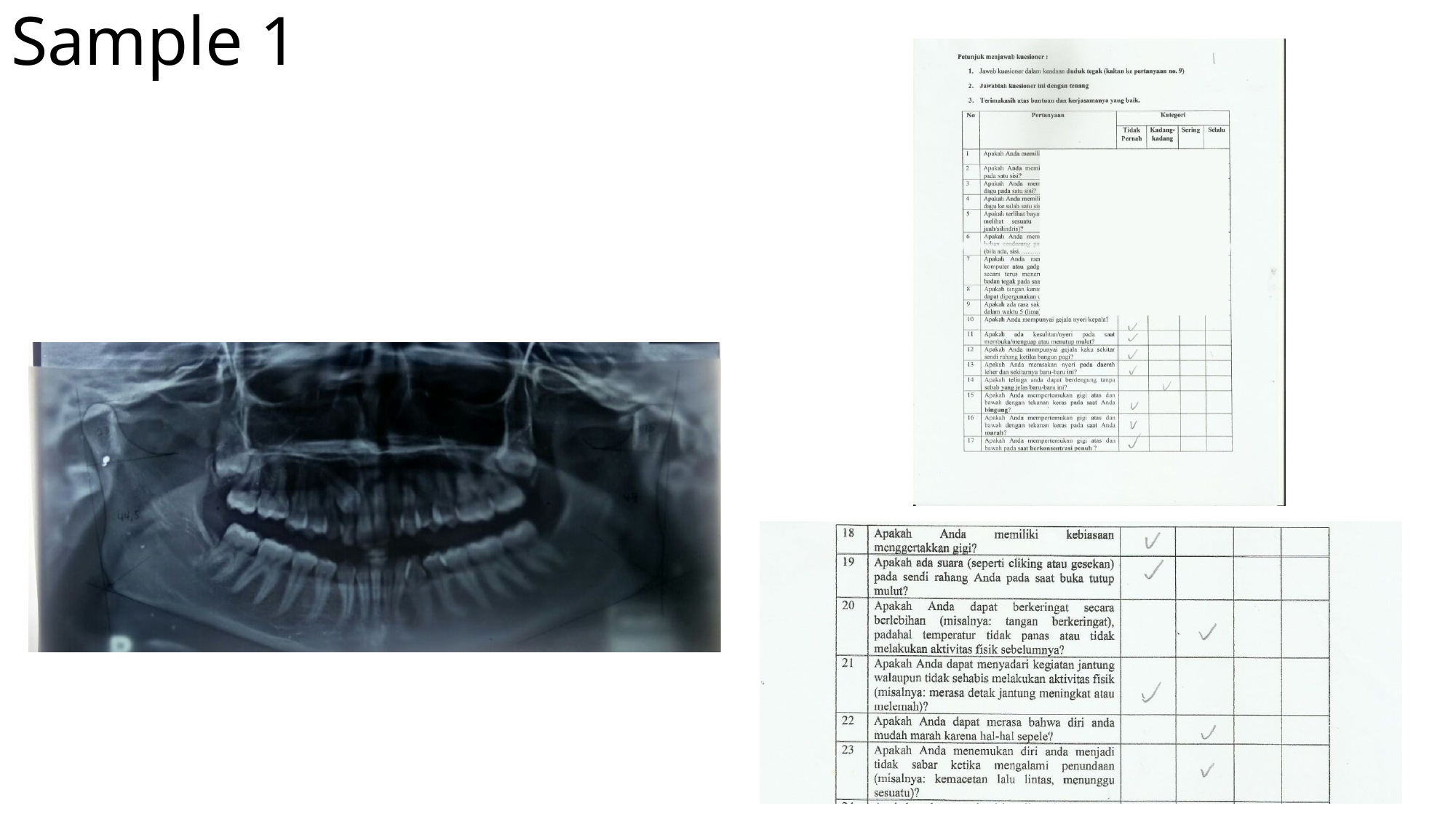

# Sample 1

## Slide 2
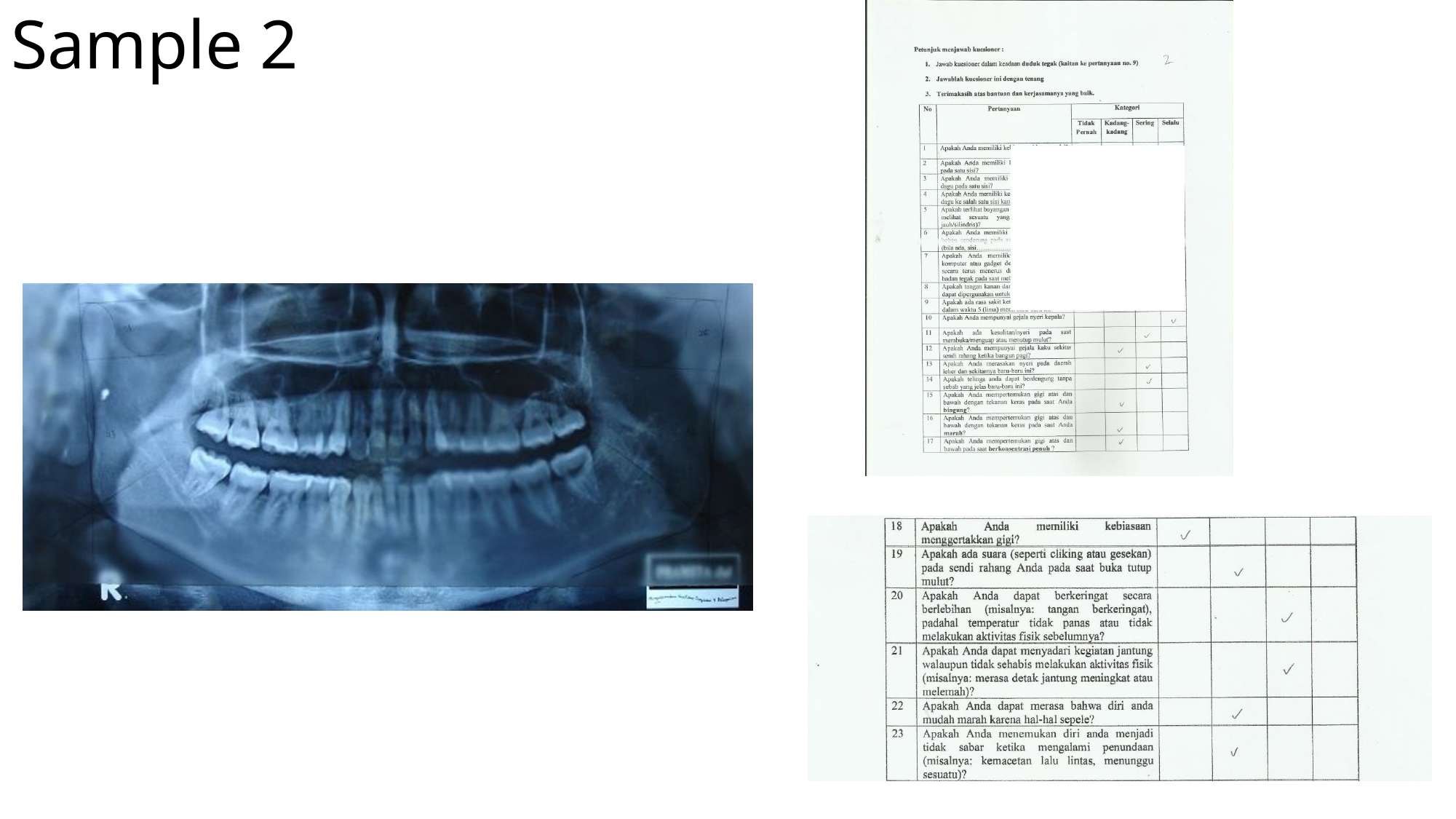

# Sample 2

## Slide 3
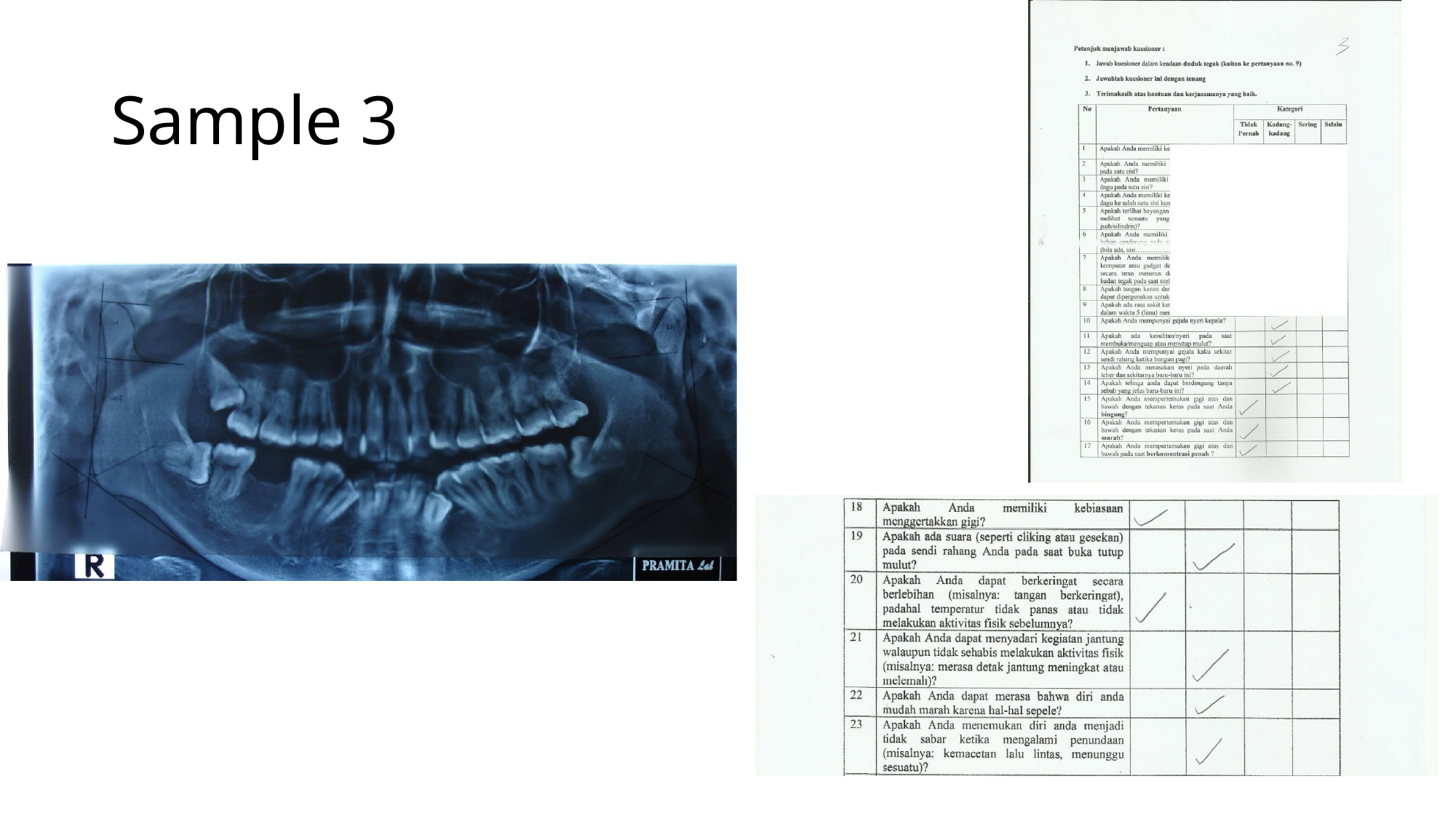

# Sample 3

## Slide 4
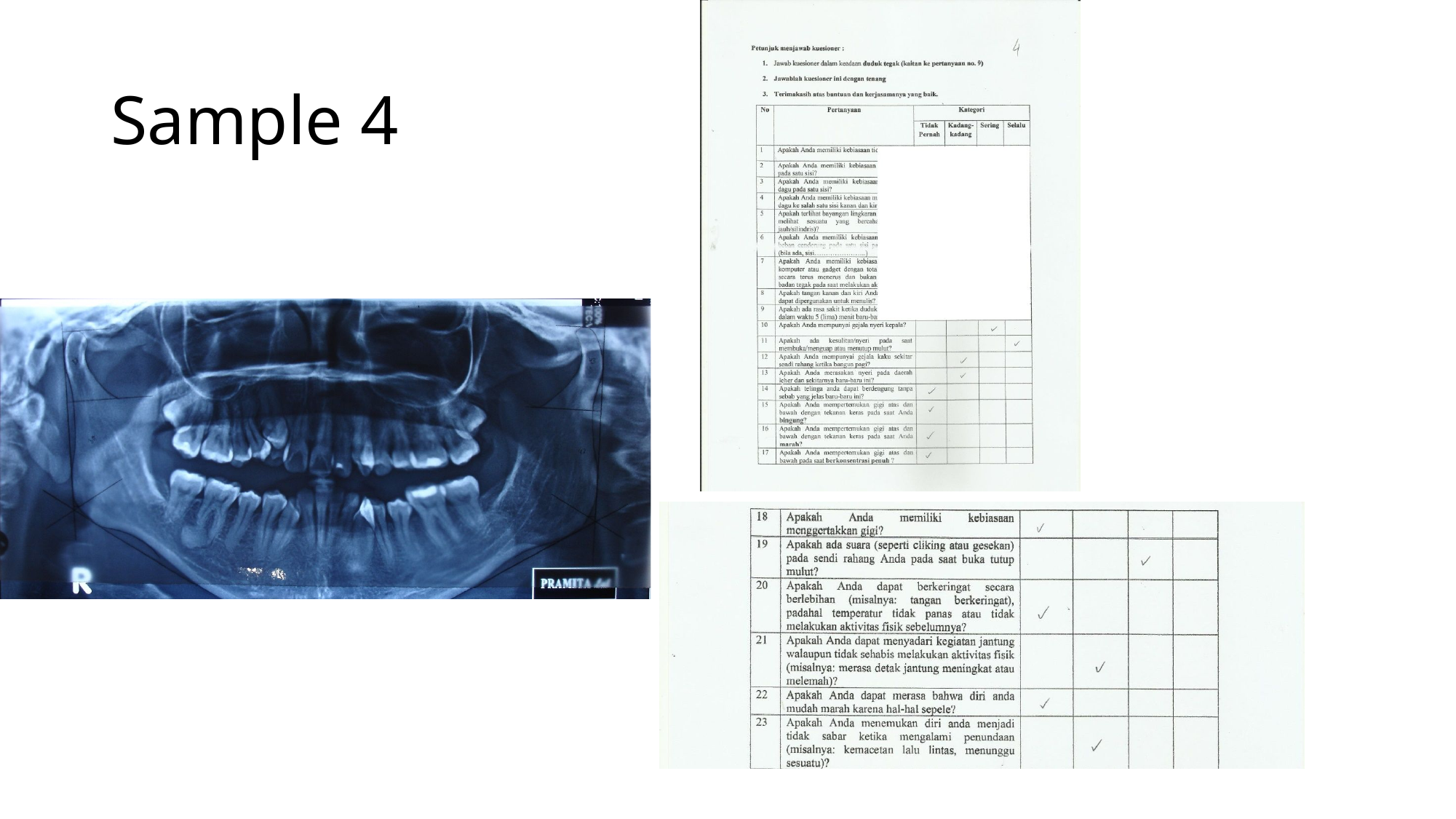

# Sample 4

## Slide 5
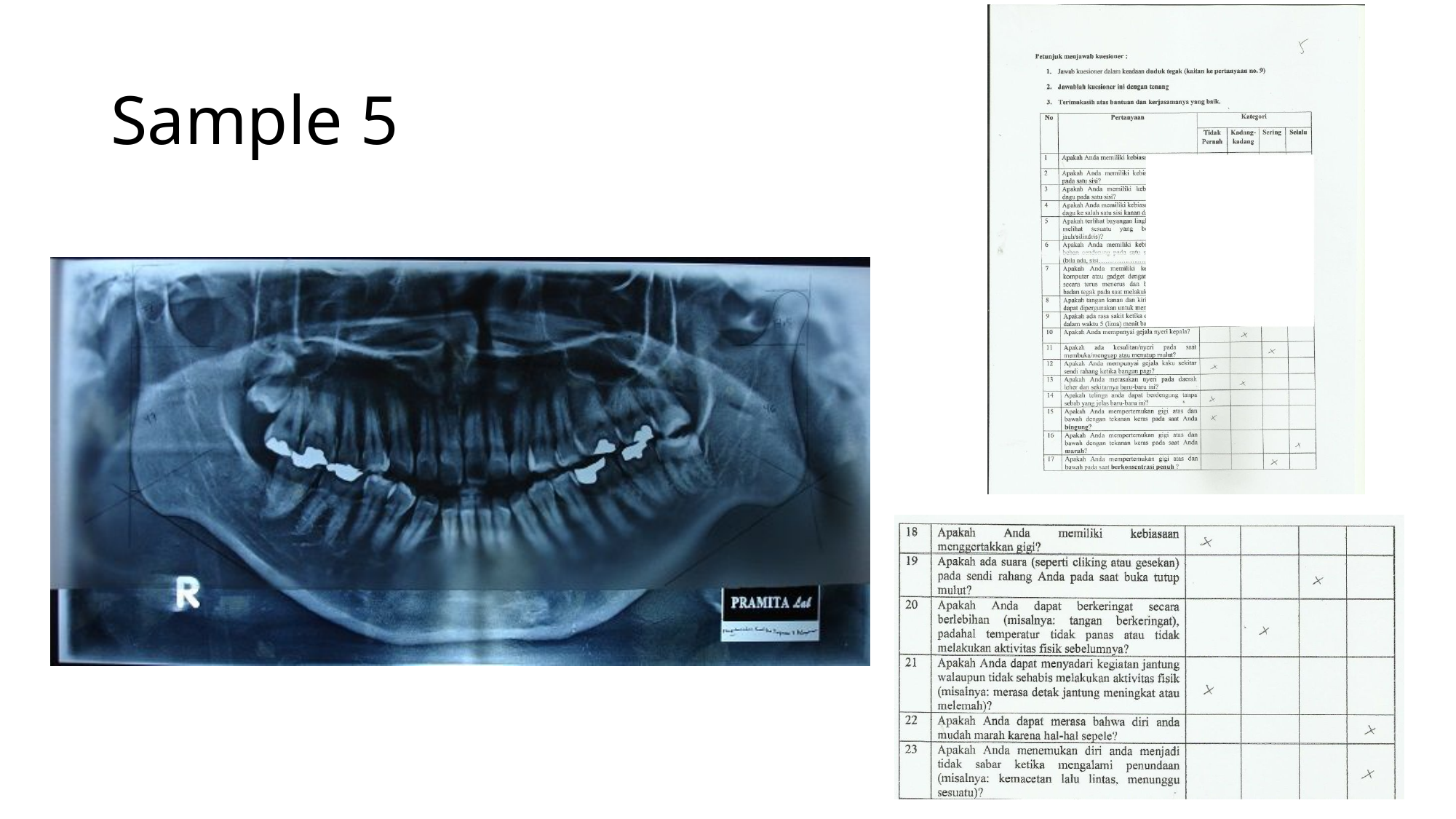

# Sample 5

## Slide 6
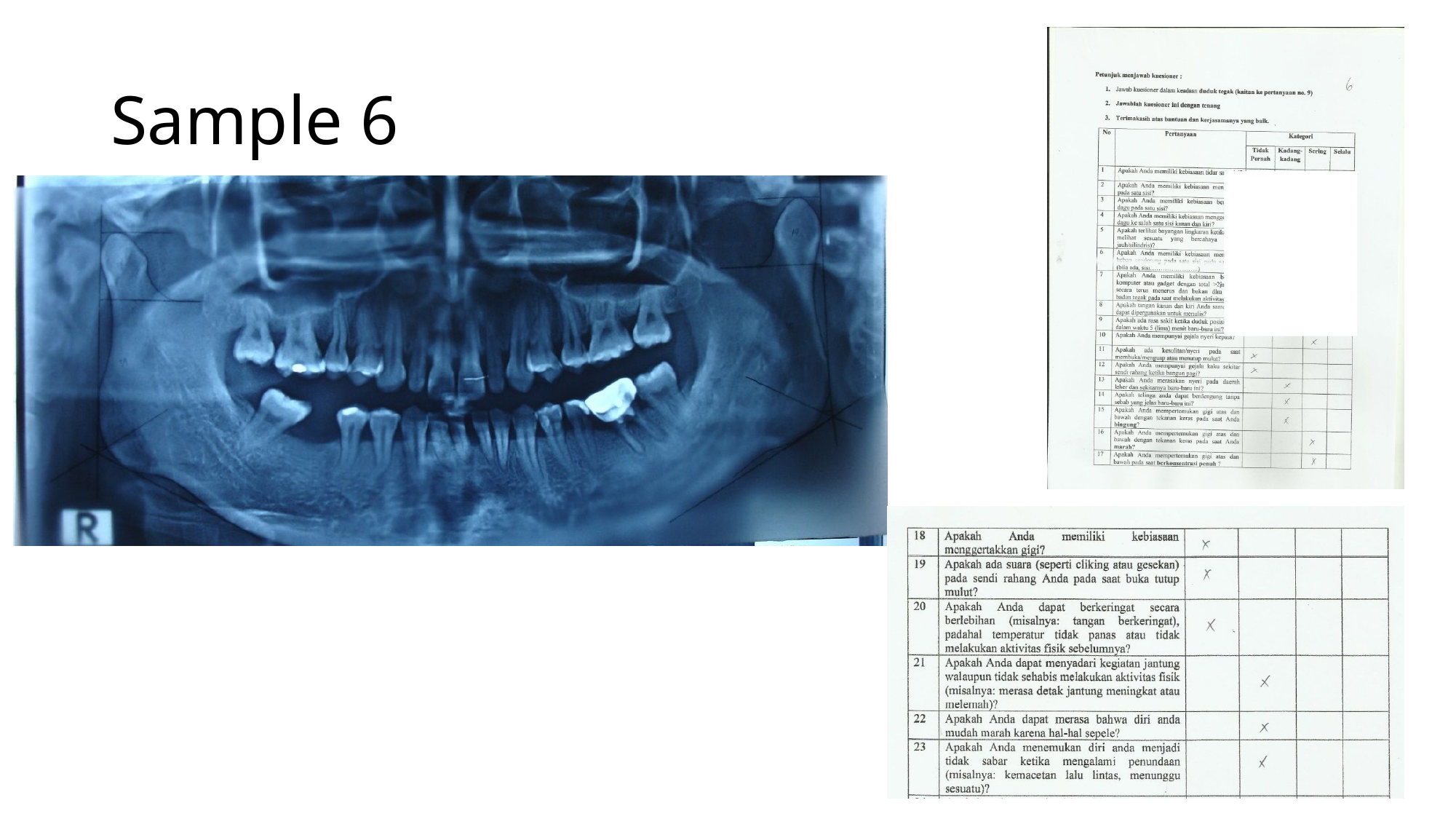

# Sample 6

## Slide 7
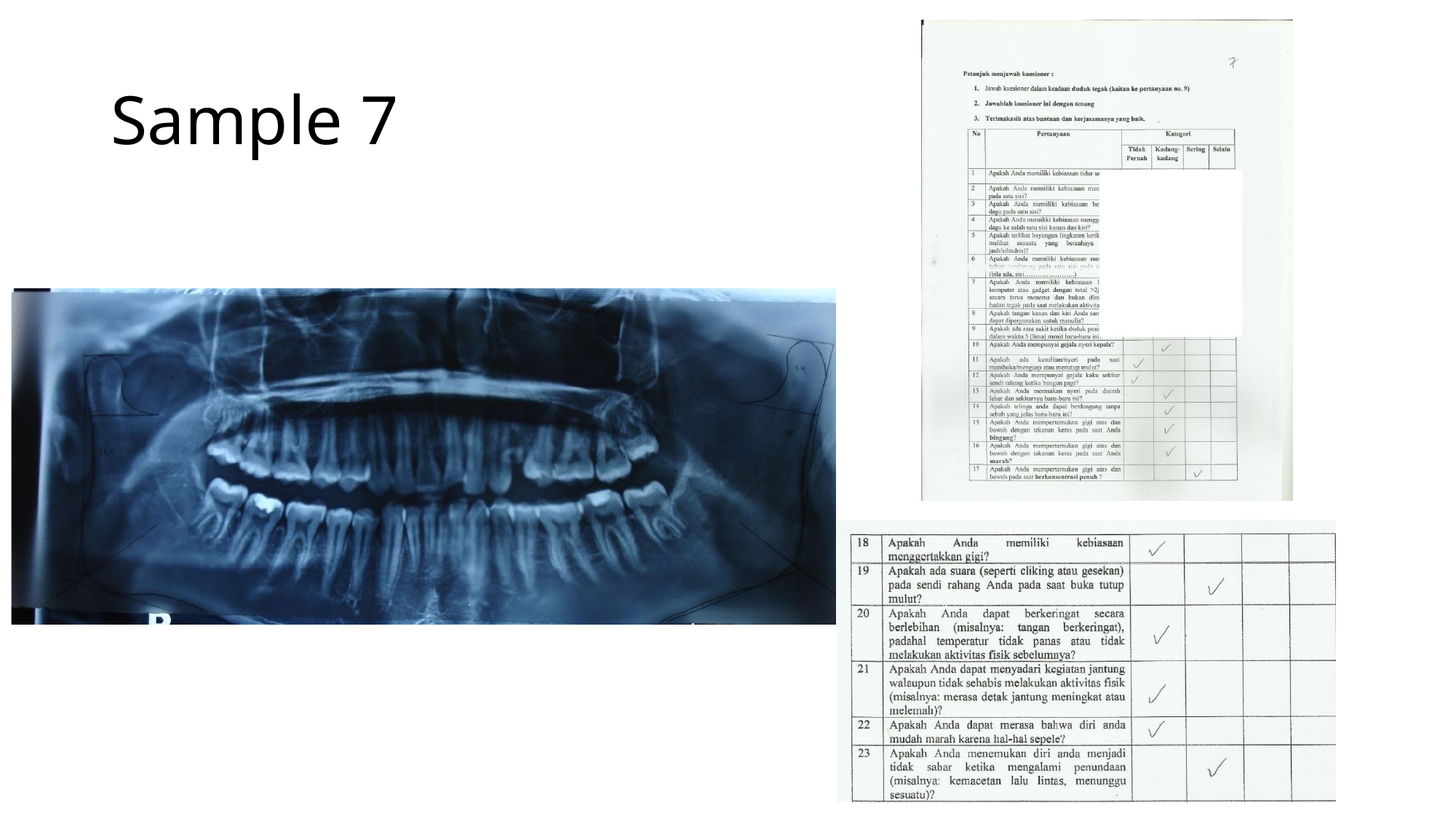

# Sample 7

## Slide 8
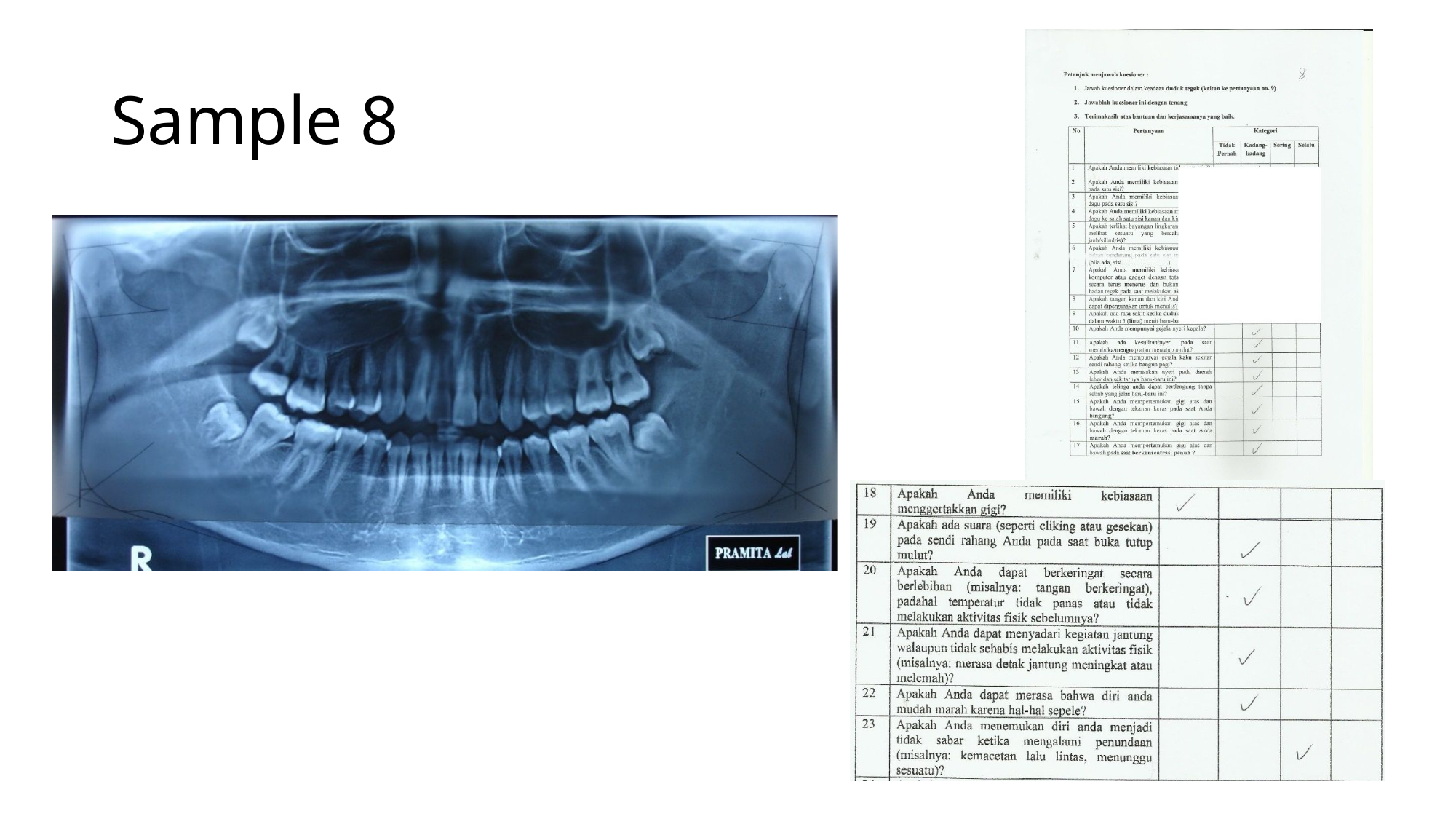

# Sample 8

## Slide 9
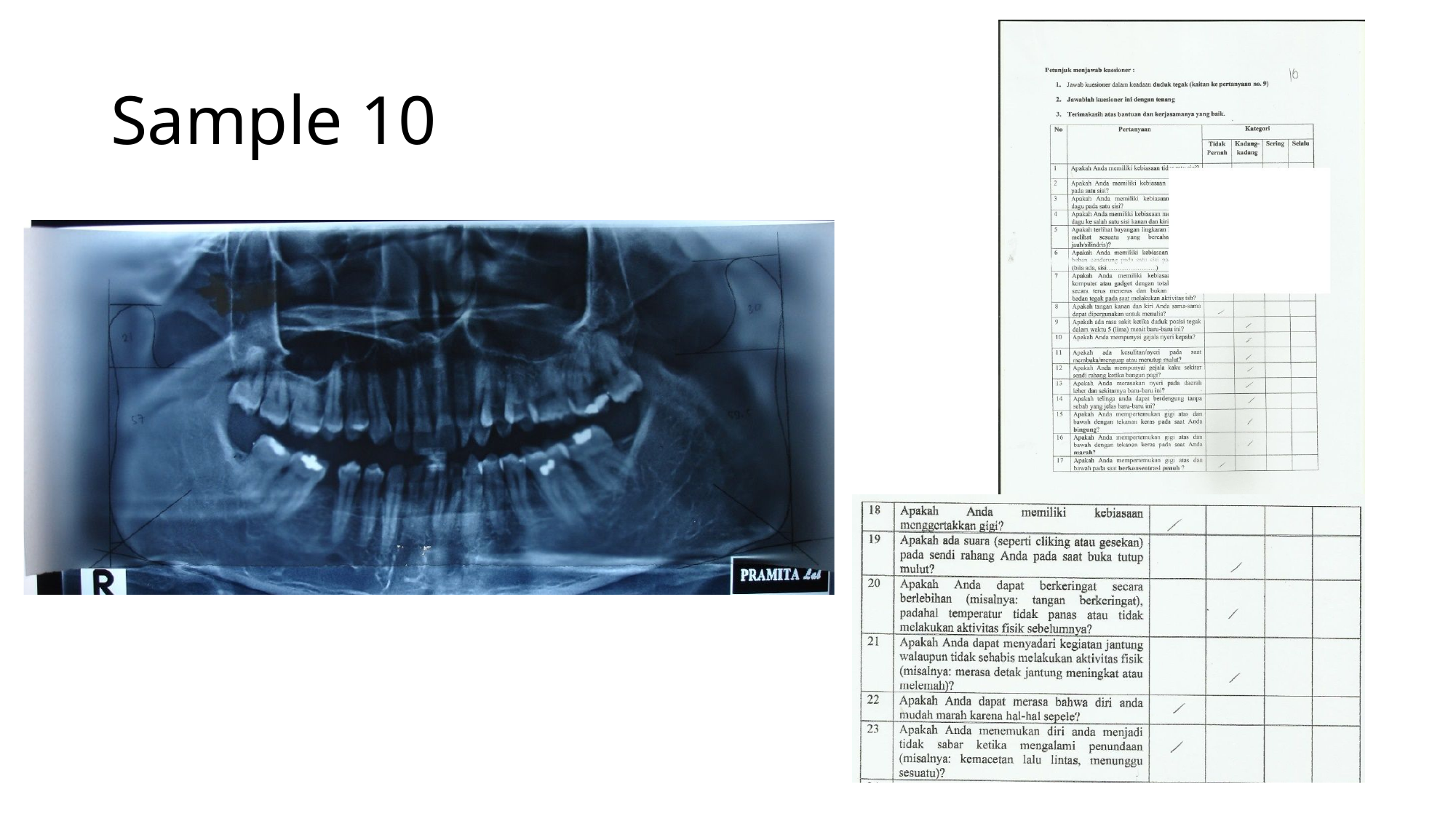

# Sample 10

## Slide 10
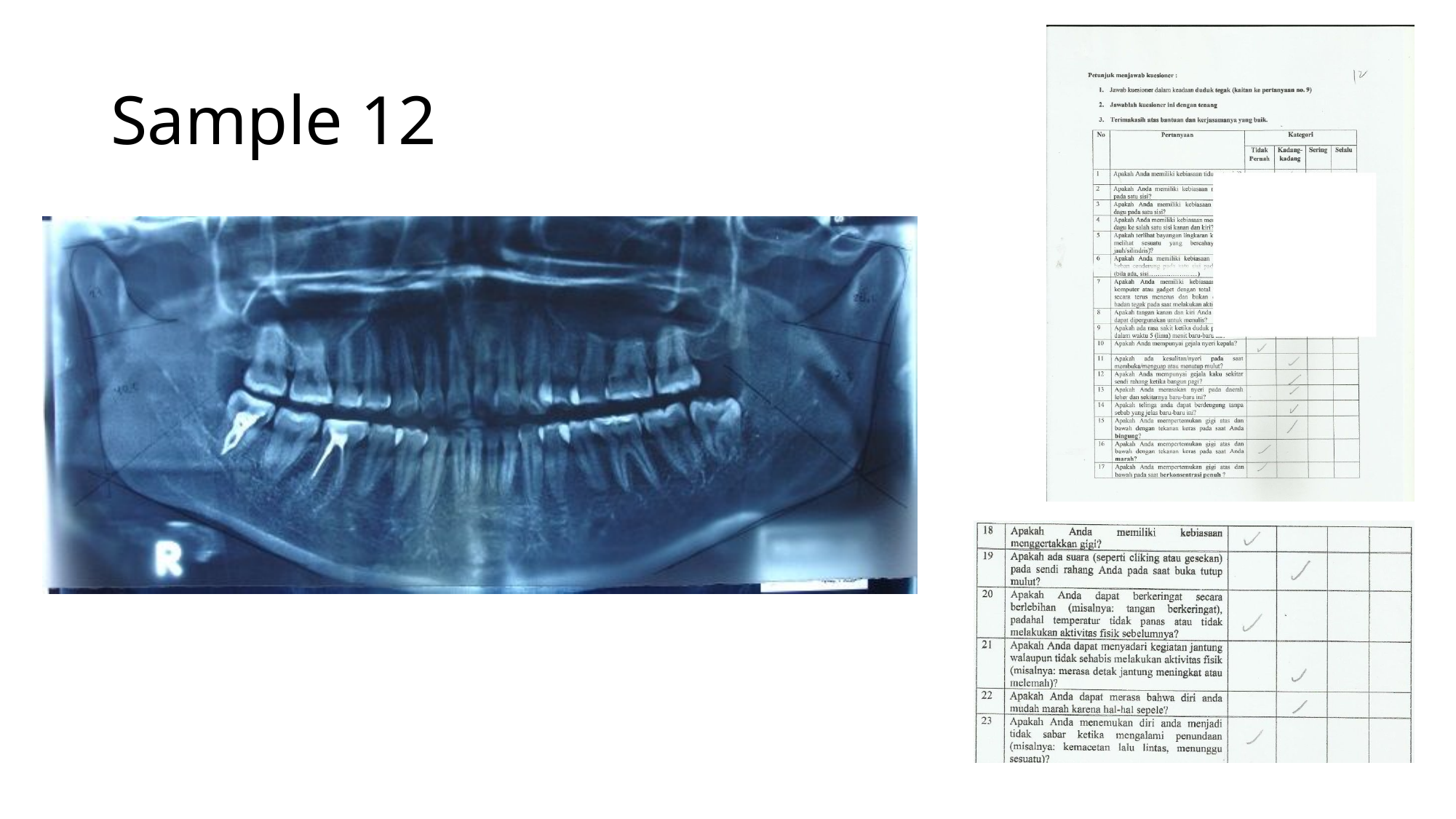

# Sample 12

## Slide 11
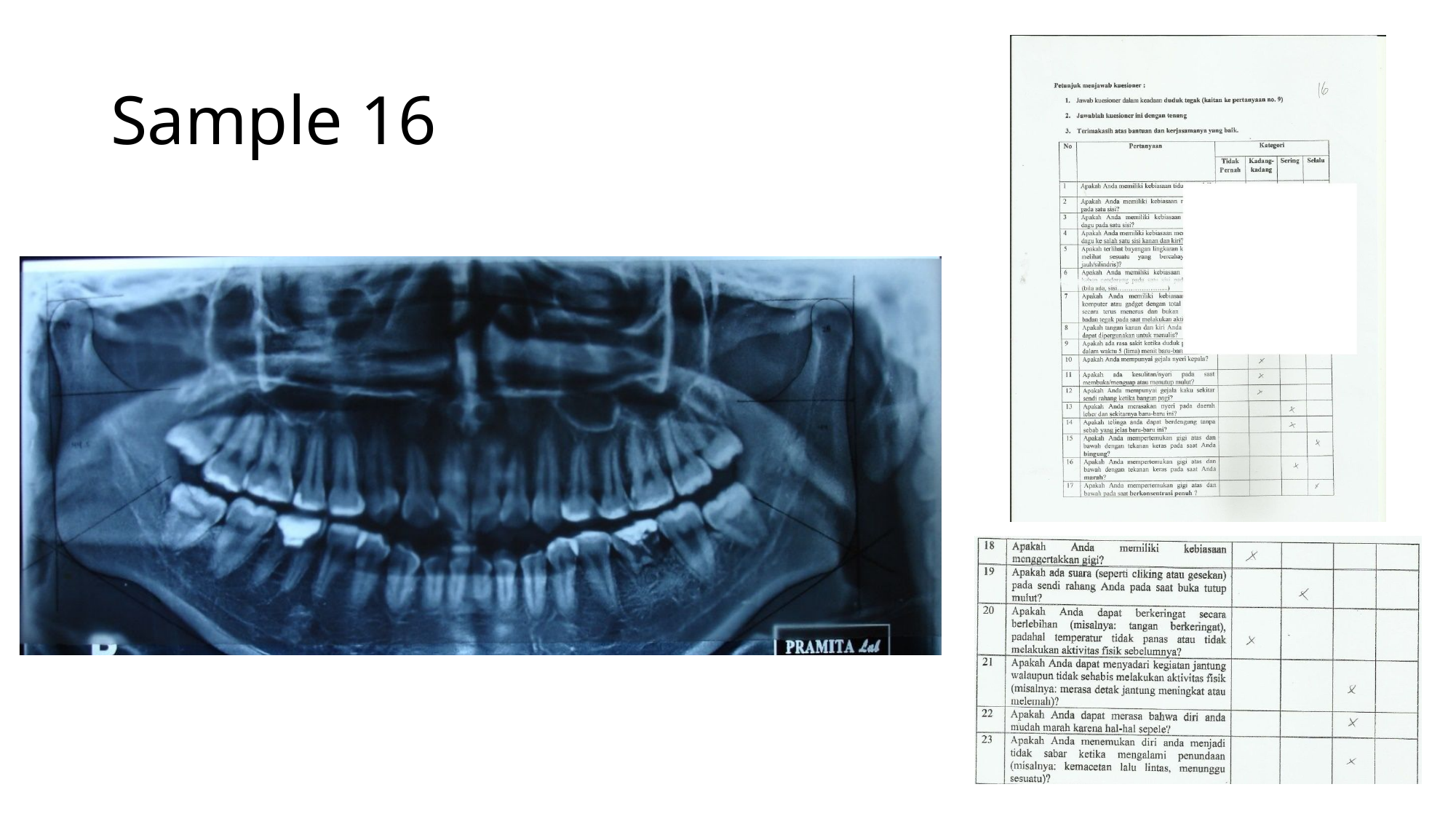

# Sample 16

## Slide 12
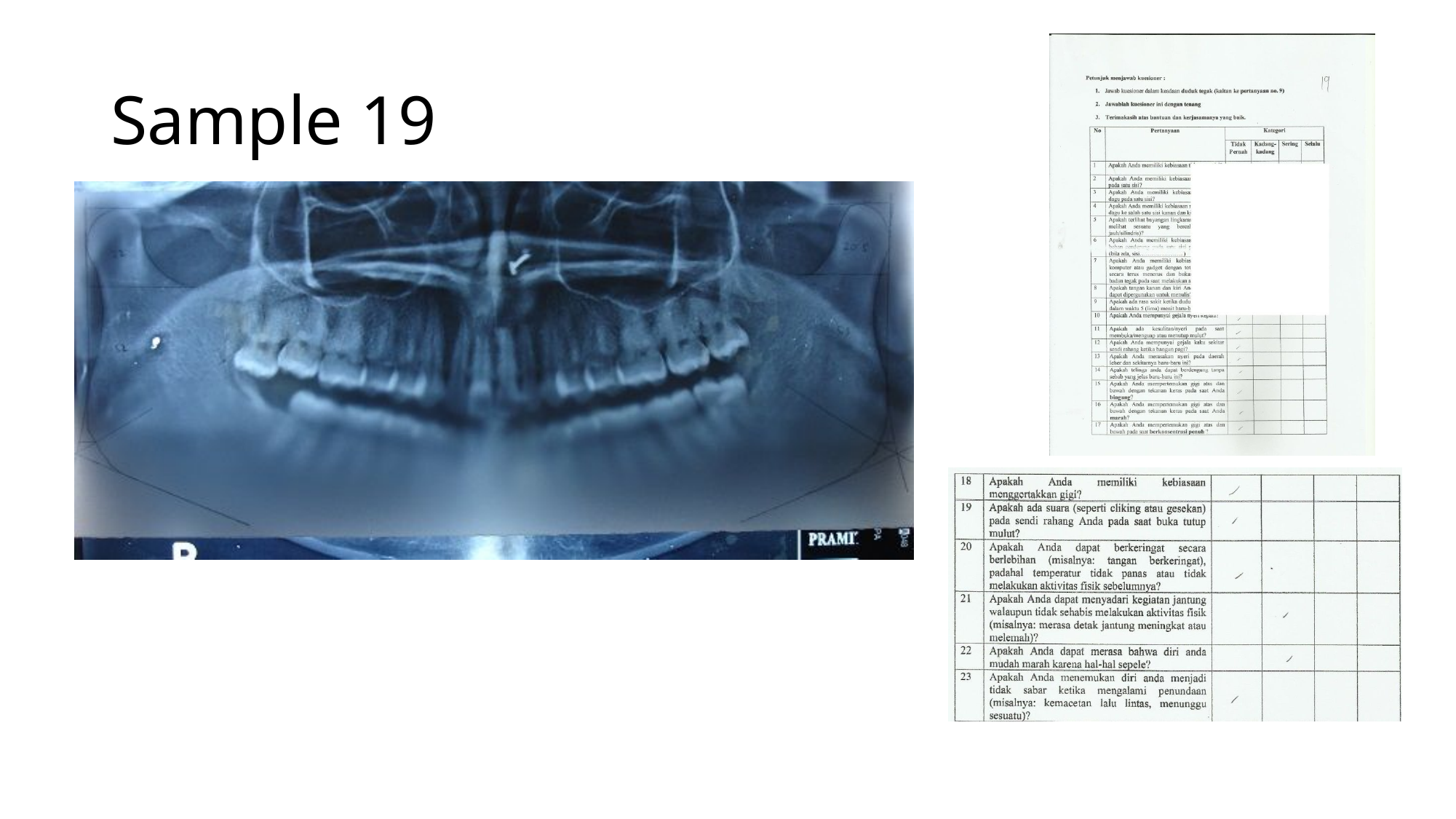

# Sample 19

## Slide 13
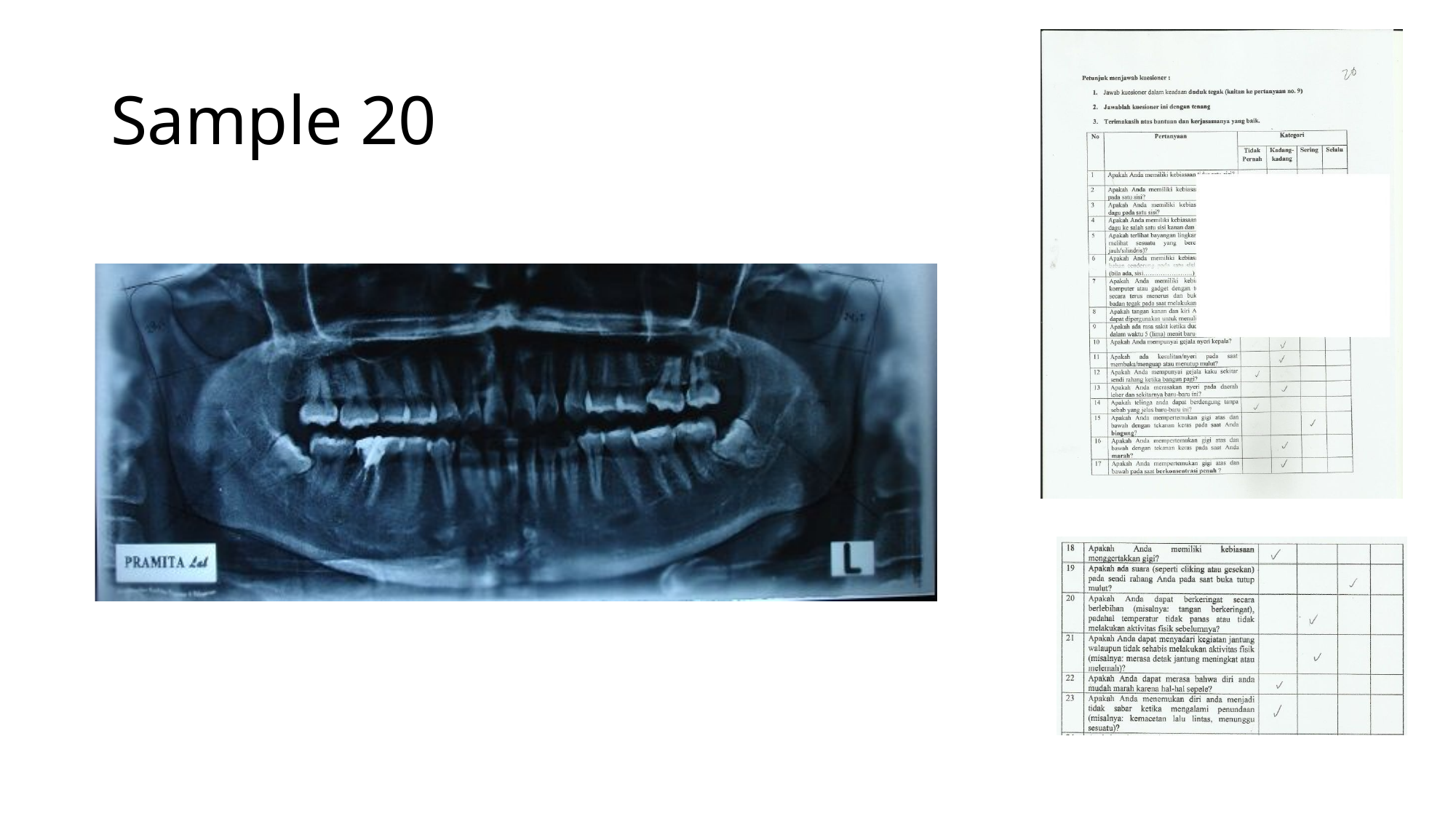

# Sample 20

## Slide 14
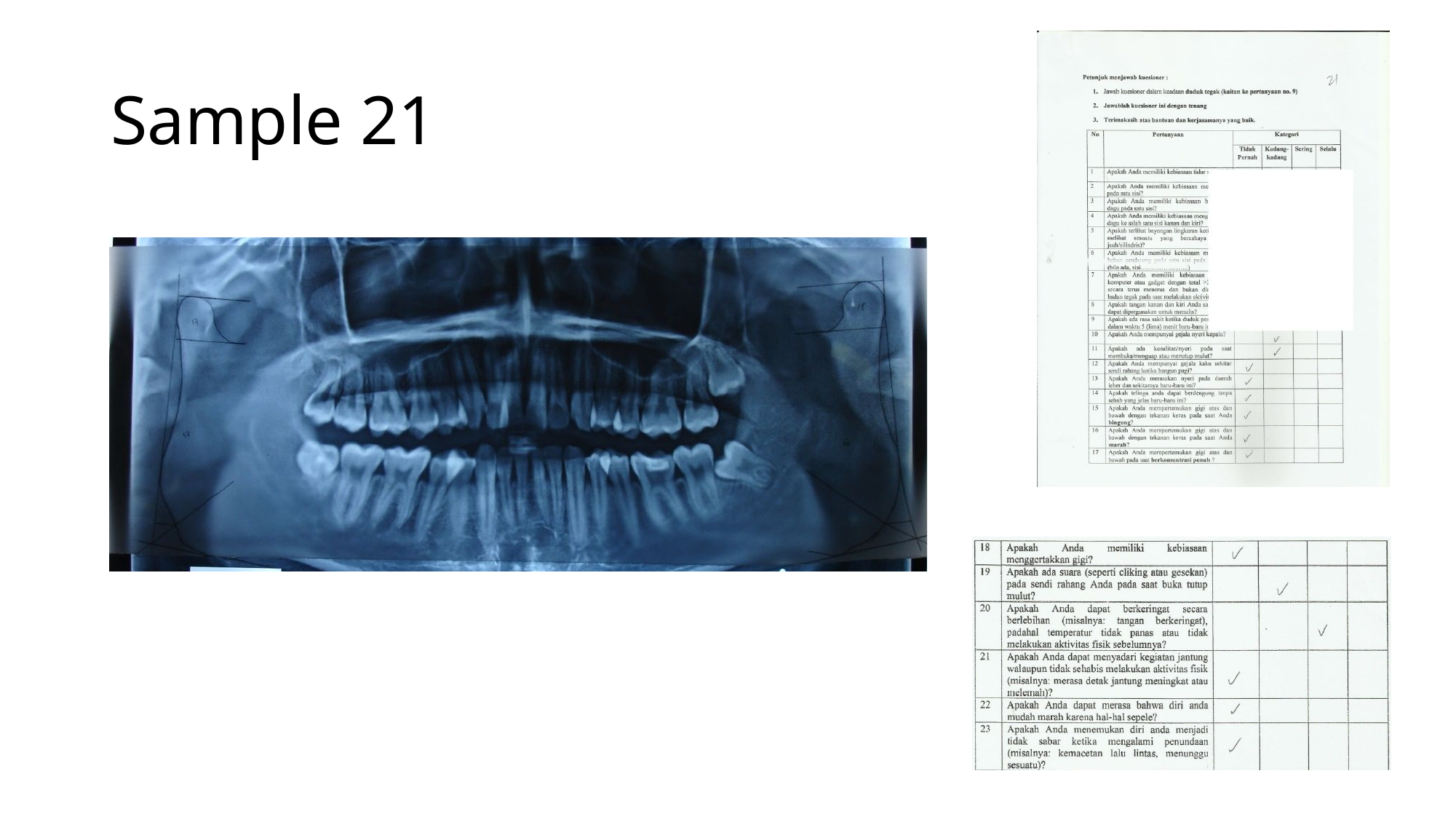

# Sample 21

## Slide 15
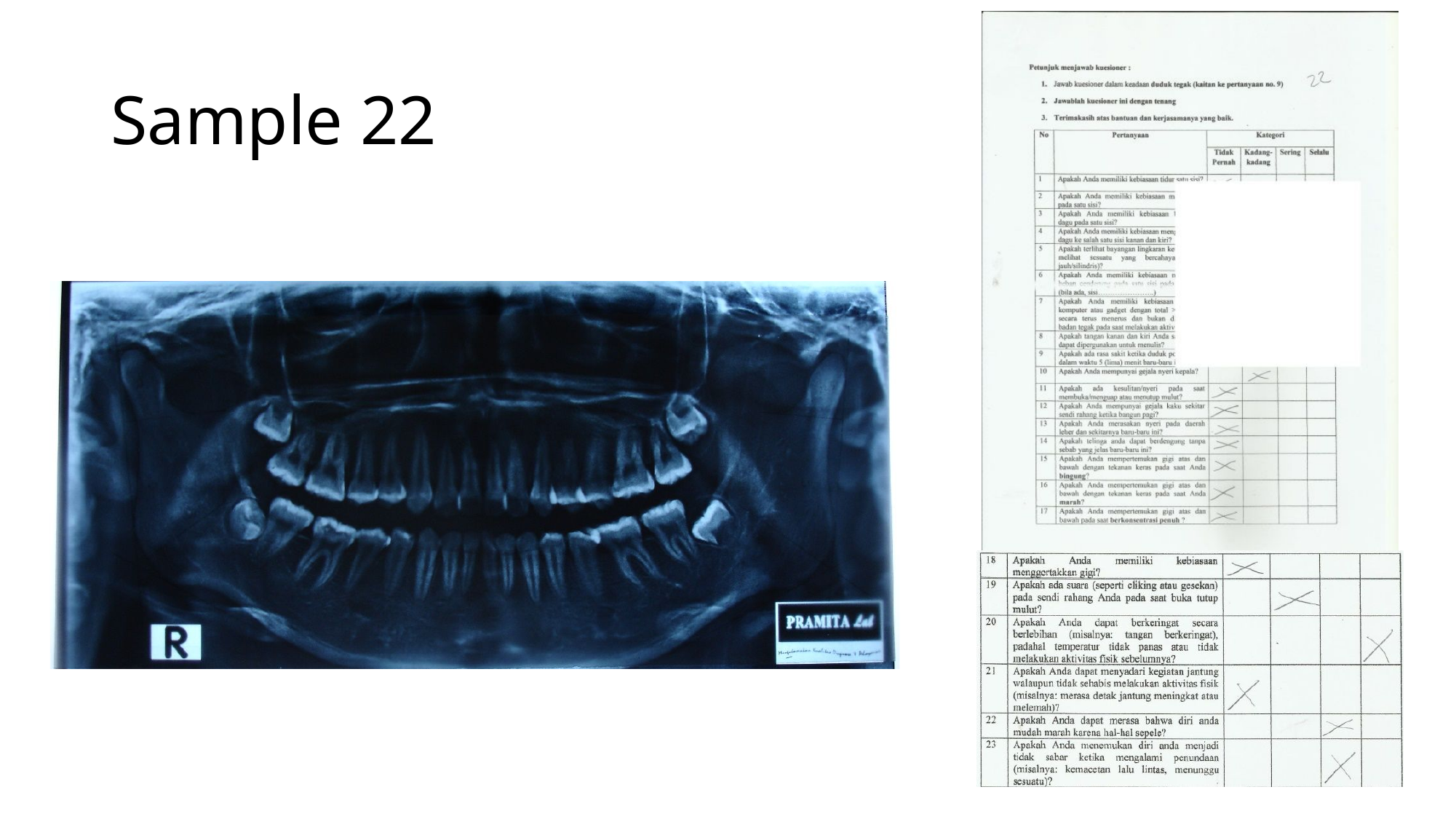

# Sample 22

## Slide 16
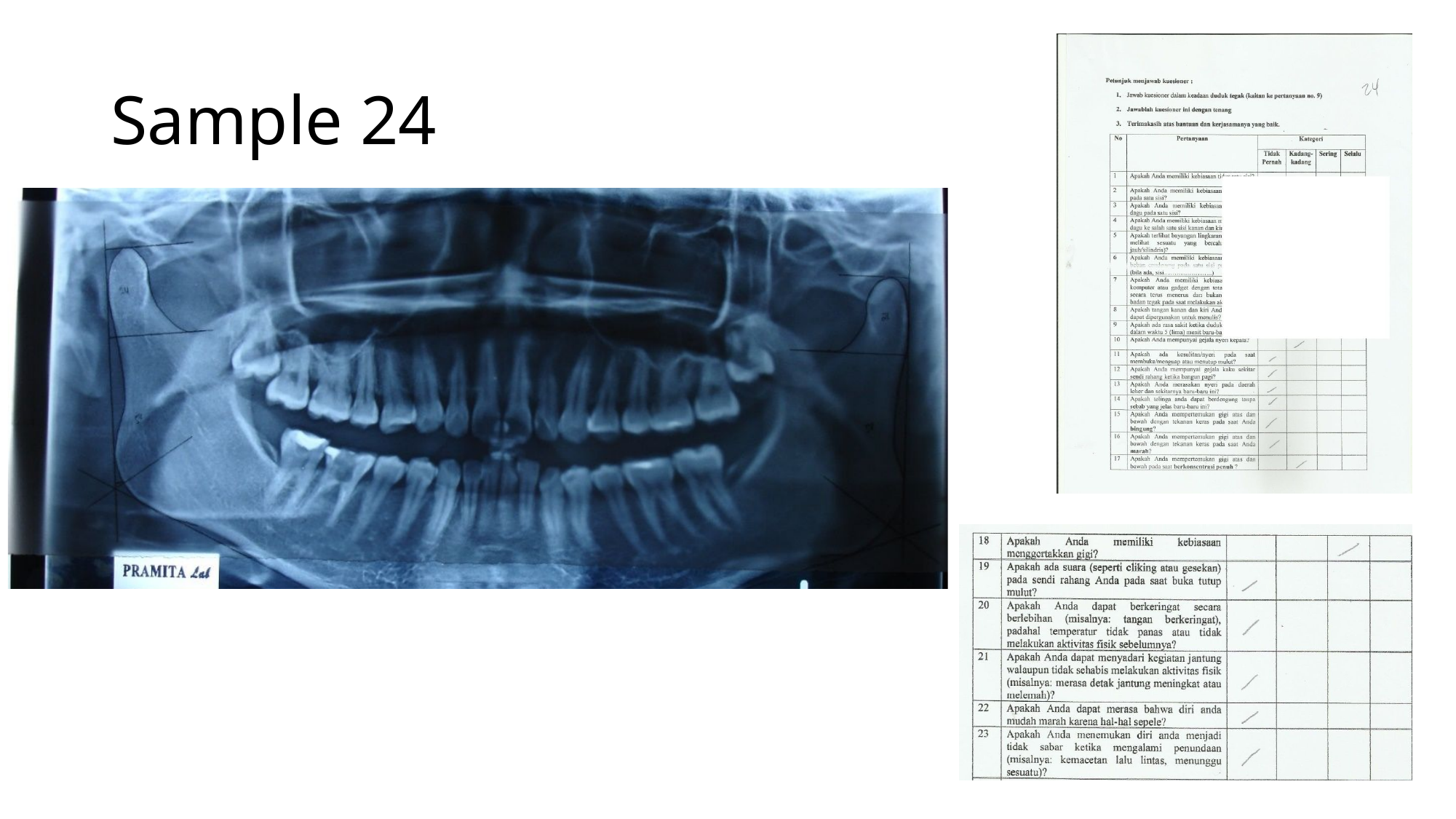

# Sample 24

## Slide 17
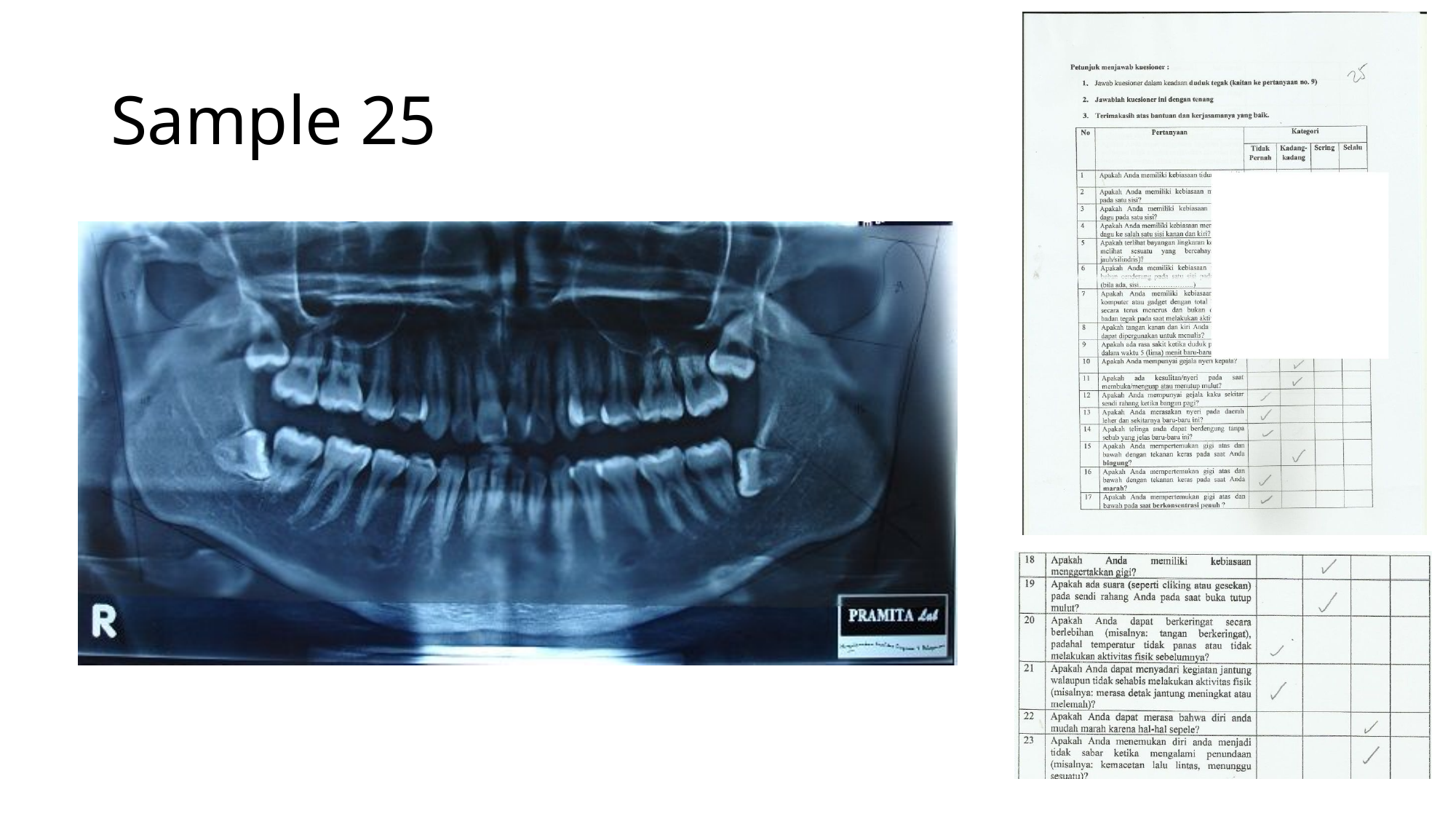

# Sample 25

## Slide 18
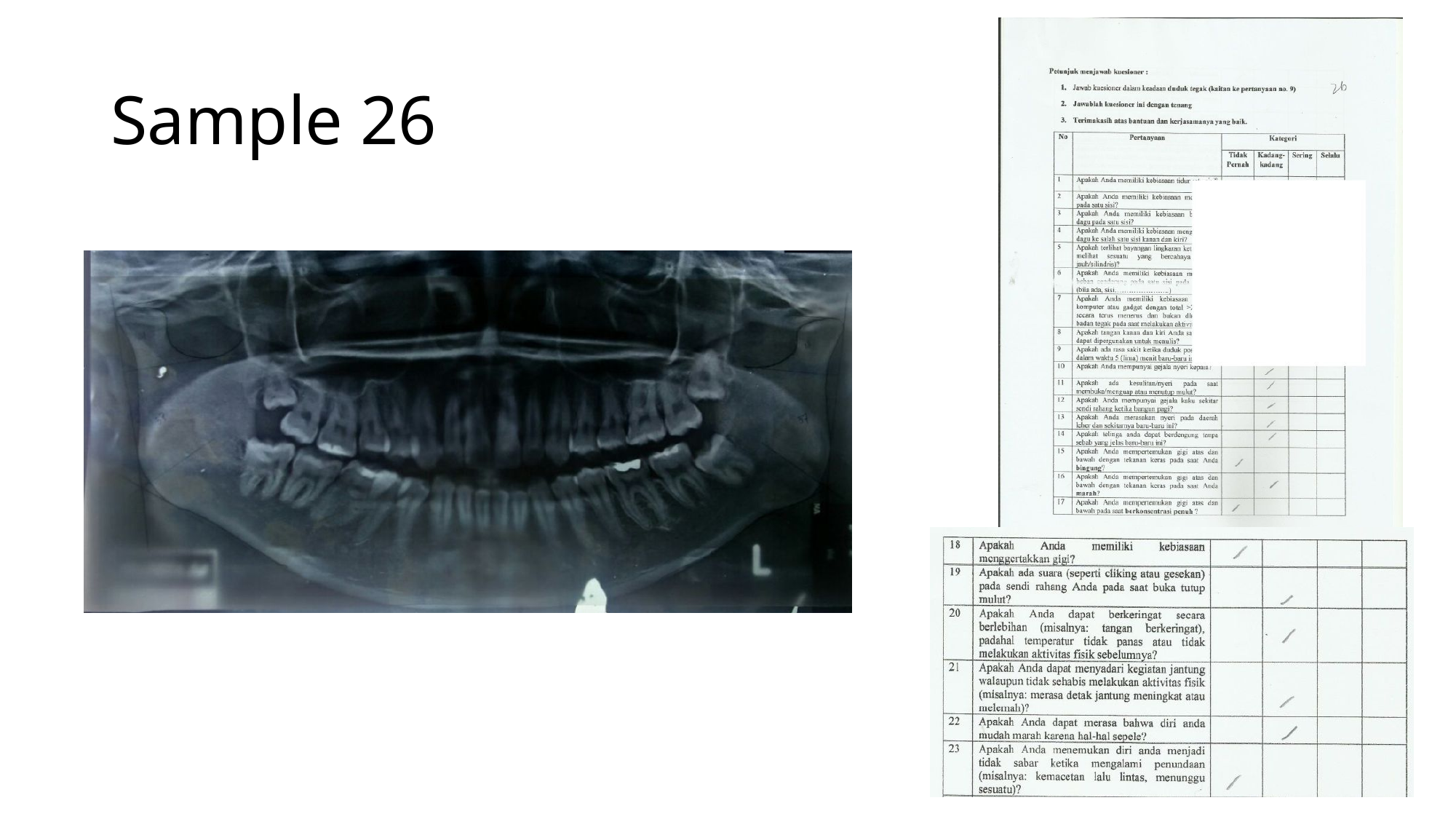

# Sample 26

## Slide 19
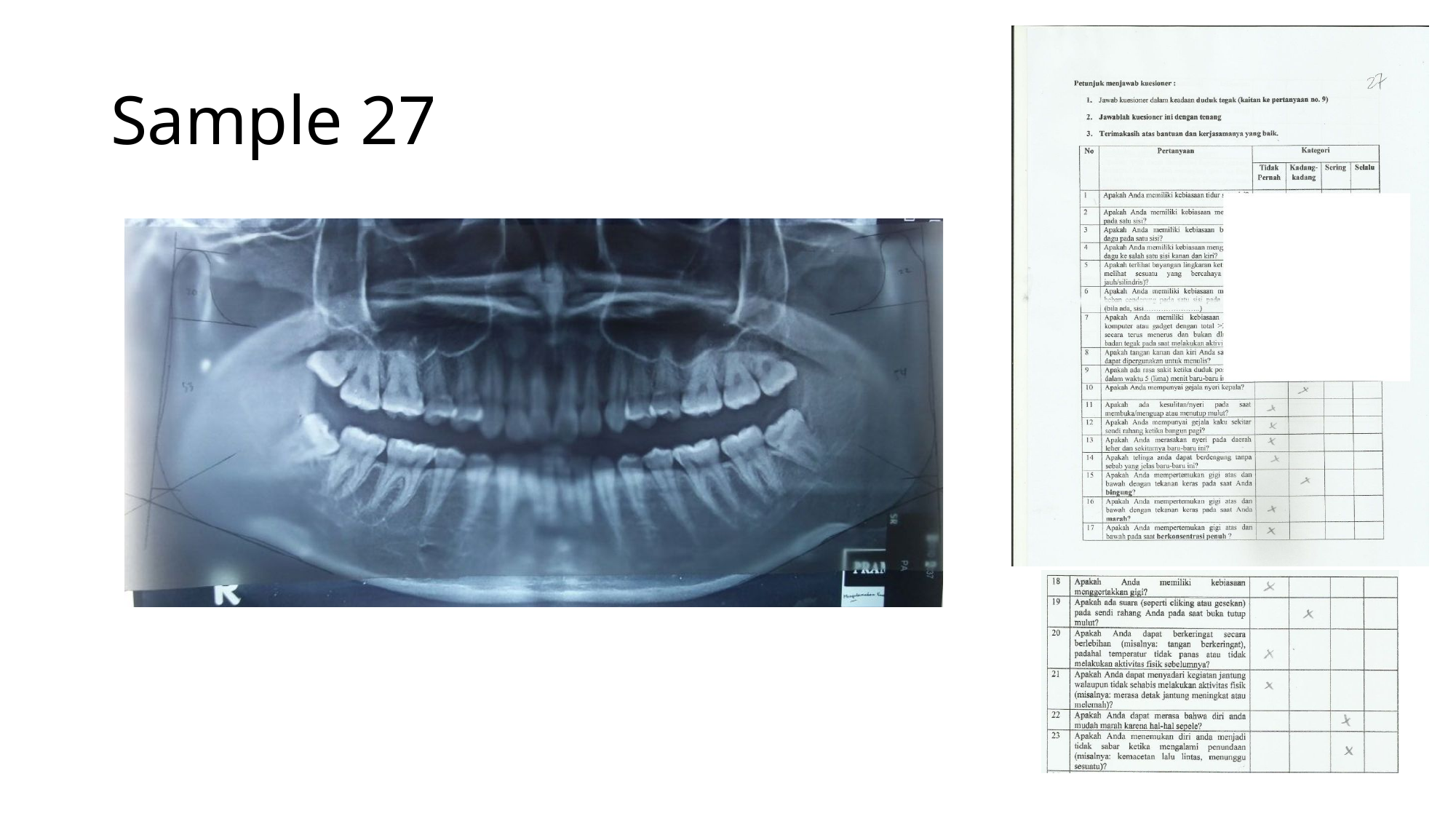

# Sample 27

## Slide 20
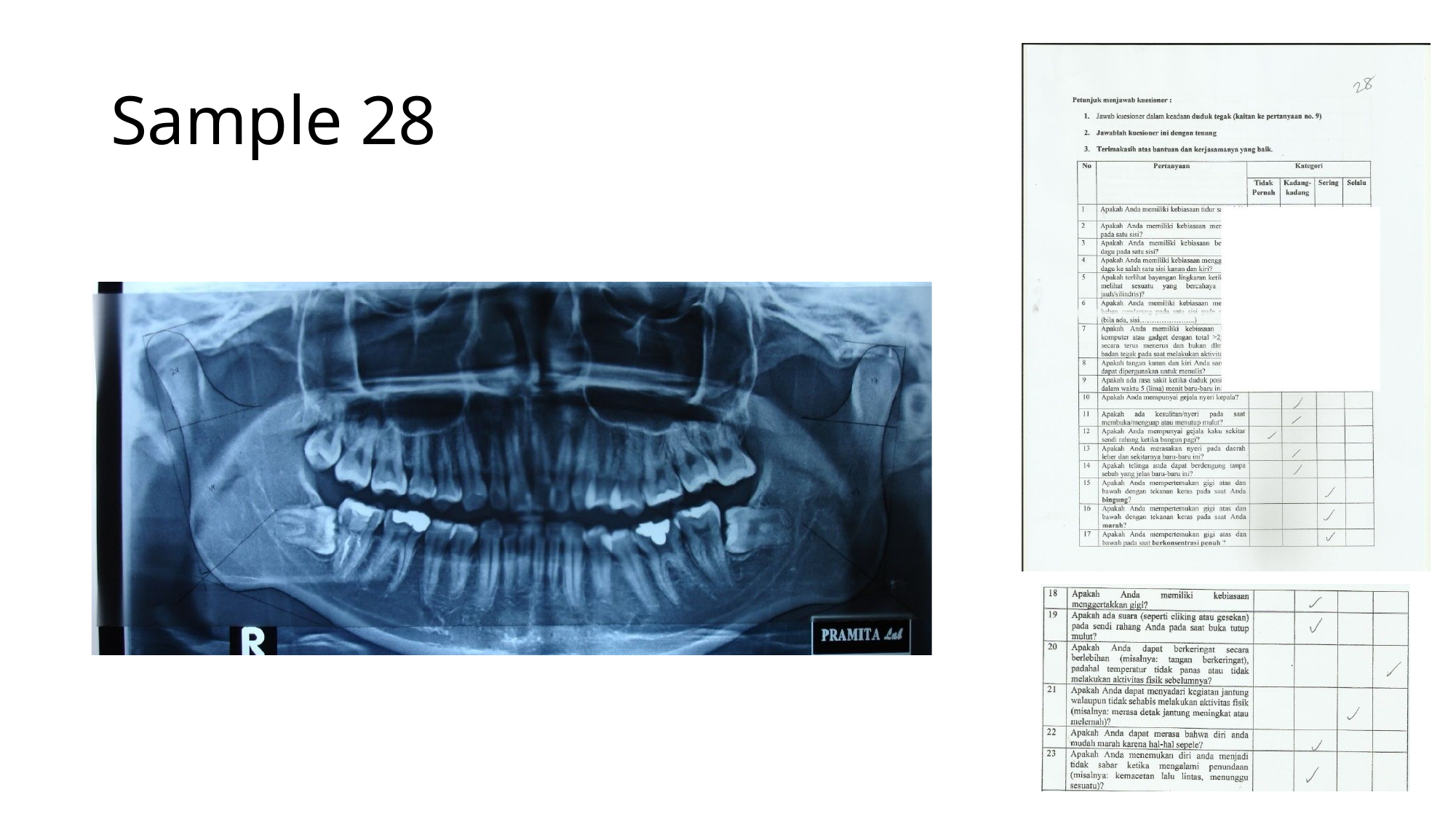

# Sample 28

## Slide 21
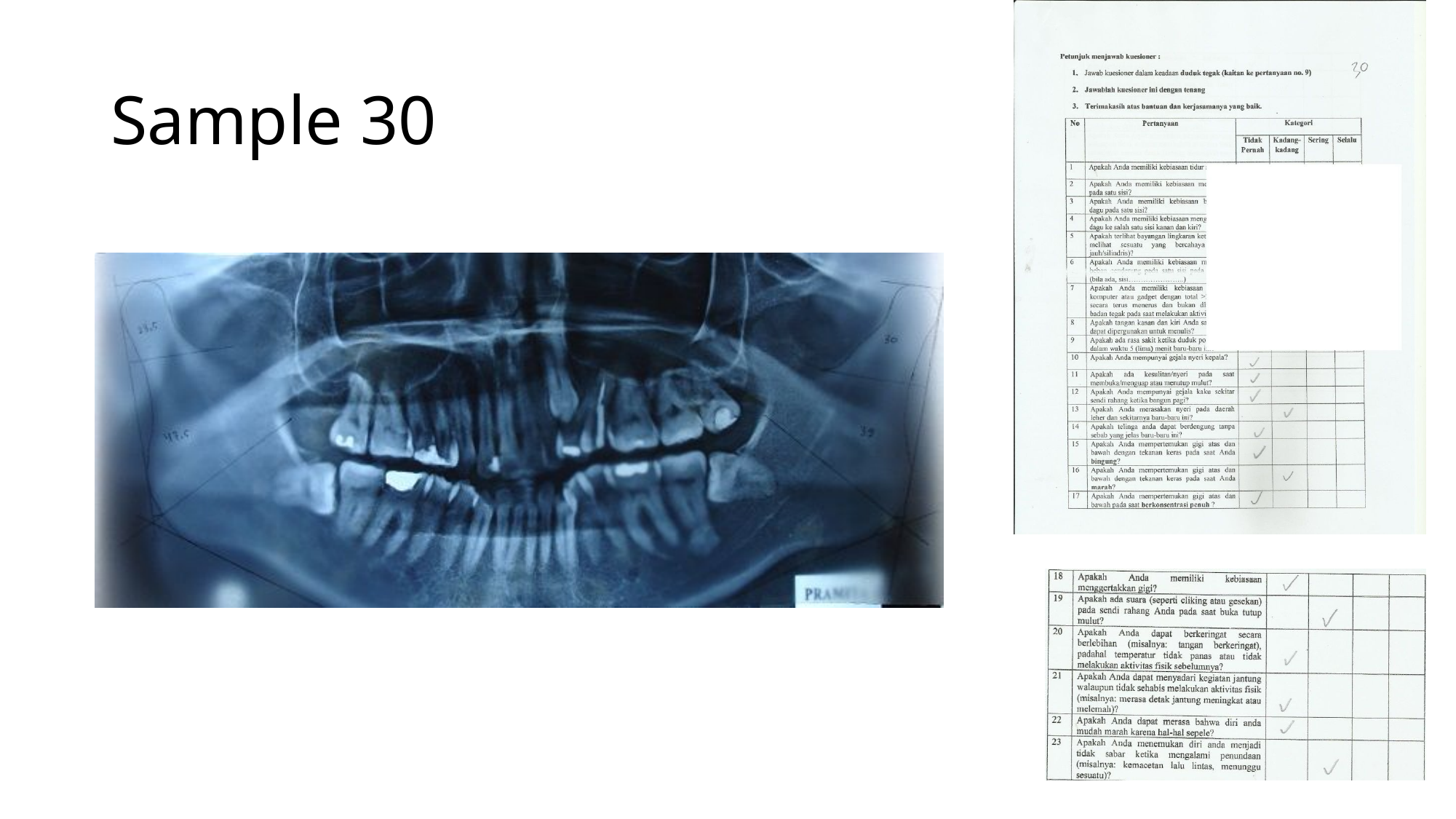

# Sample 30

## Slide 22
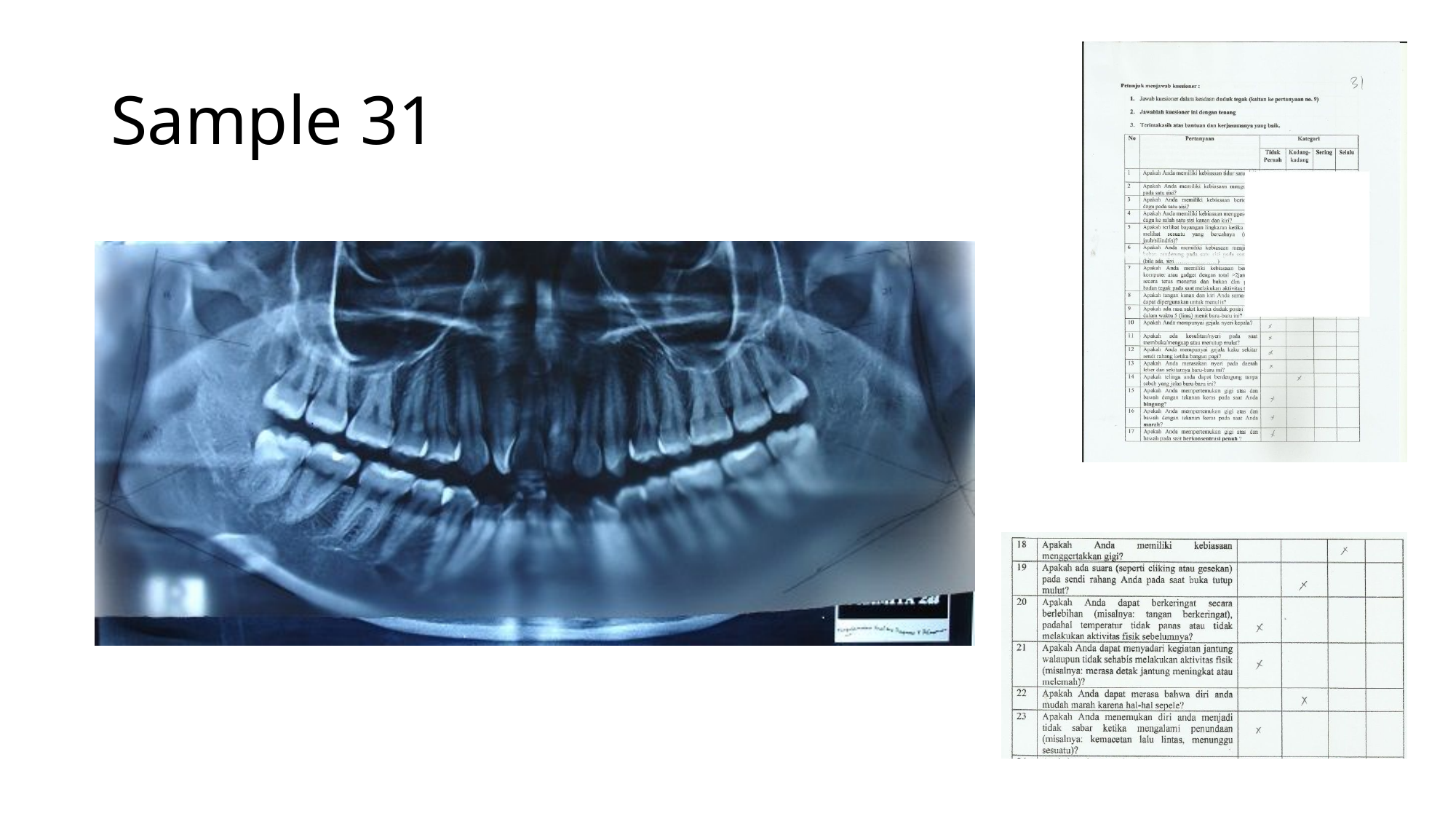

# Sample 31

## Slide 23
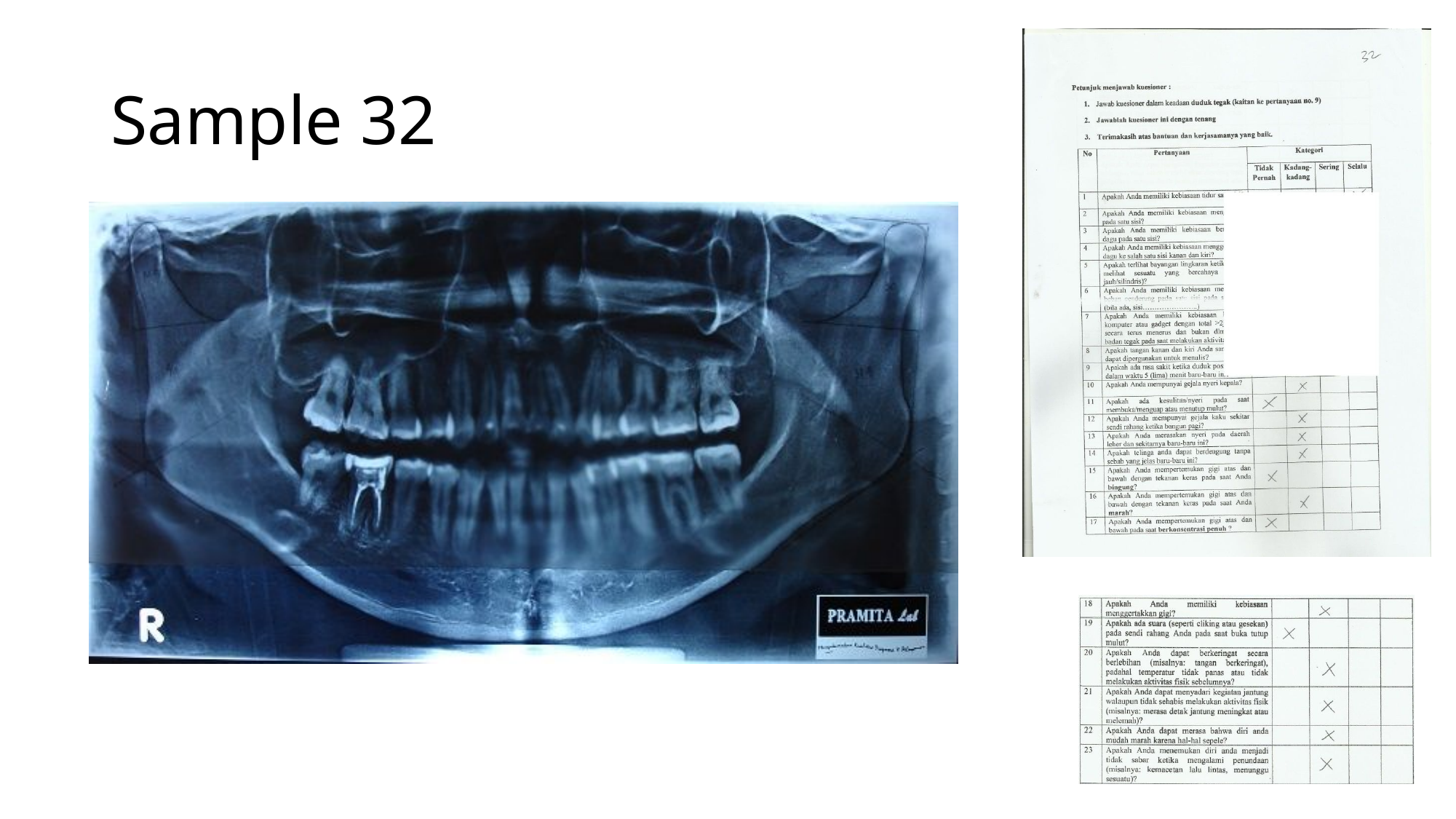

# Sample 32

## Slide 24
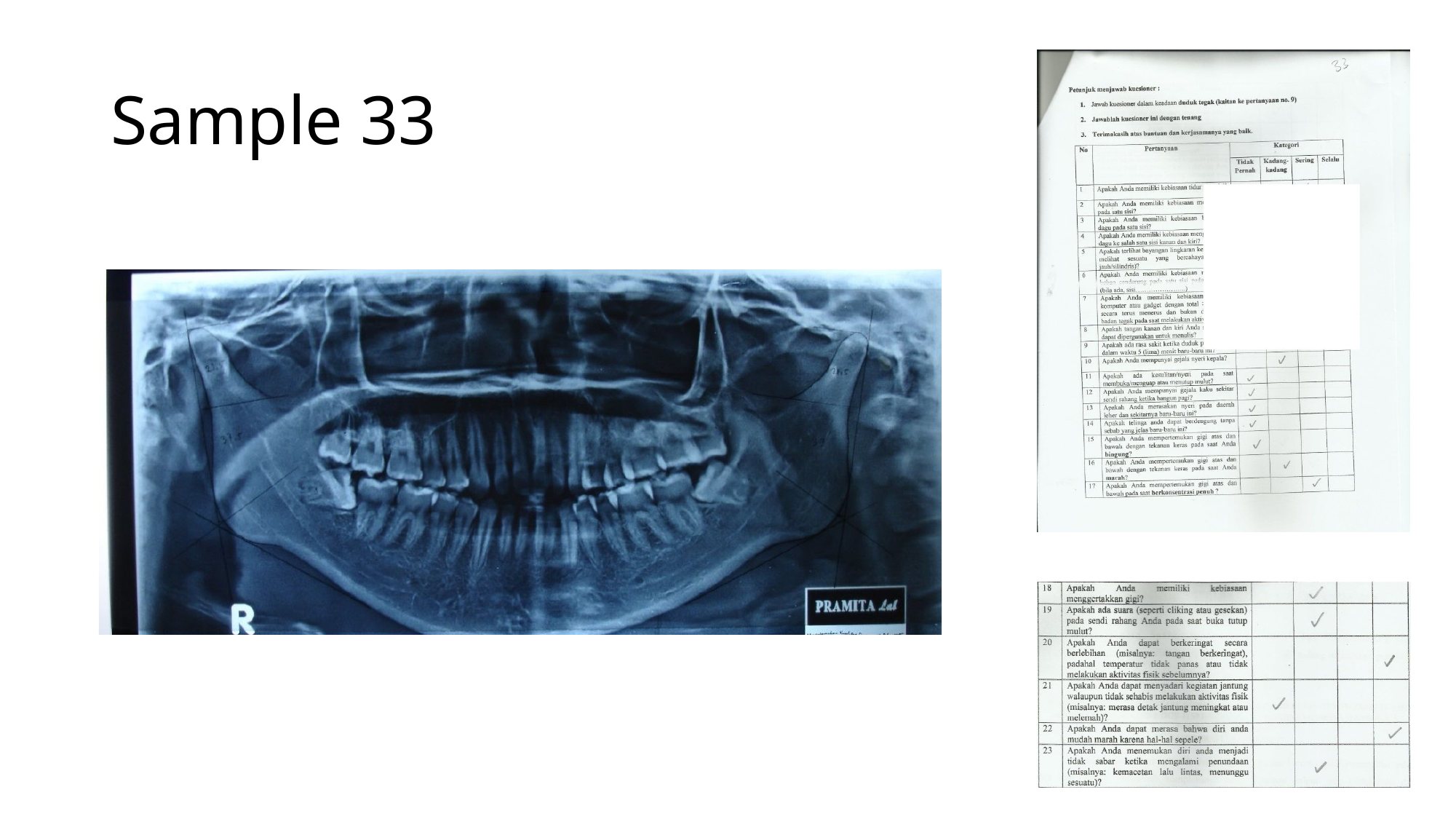

# Sample 33

## Slide 25
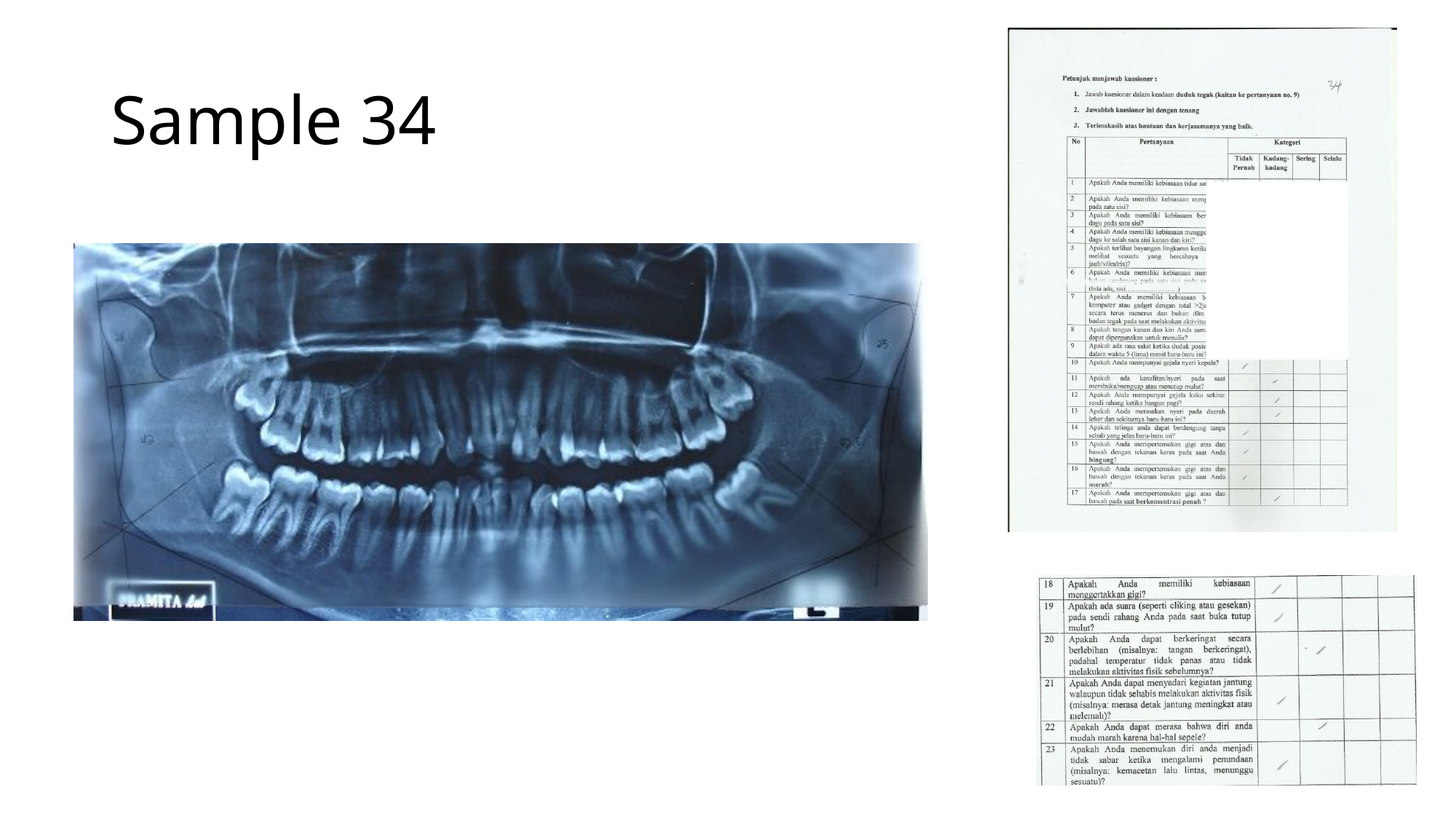

# Sample 34

## Slide 26
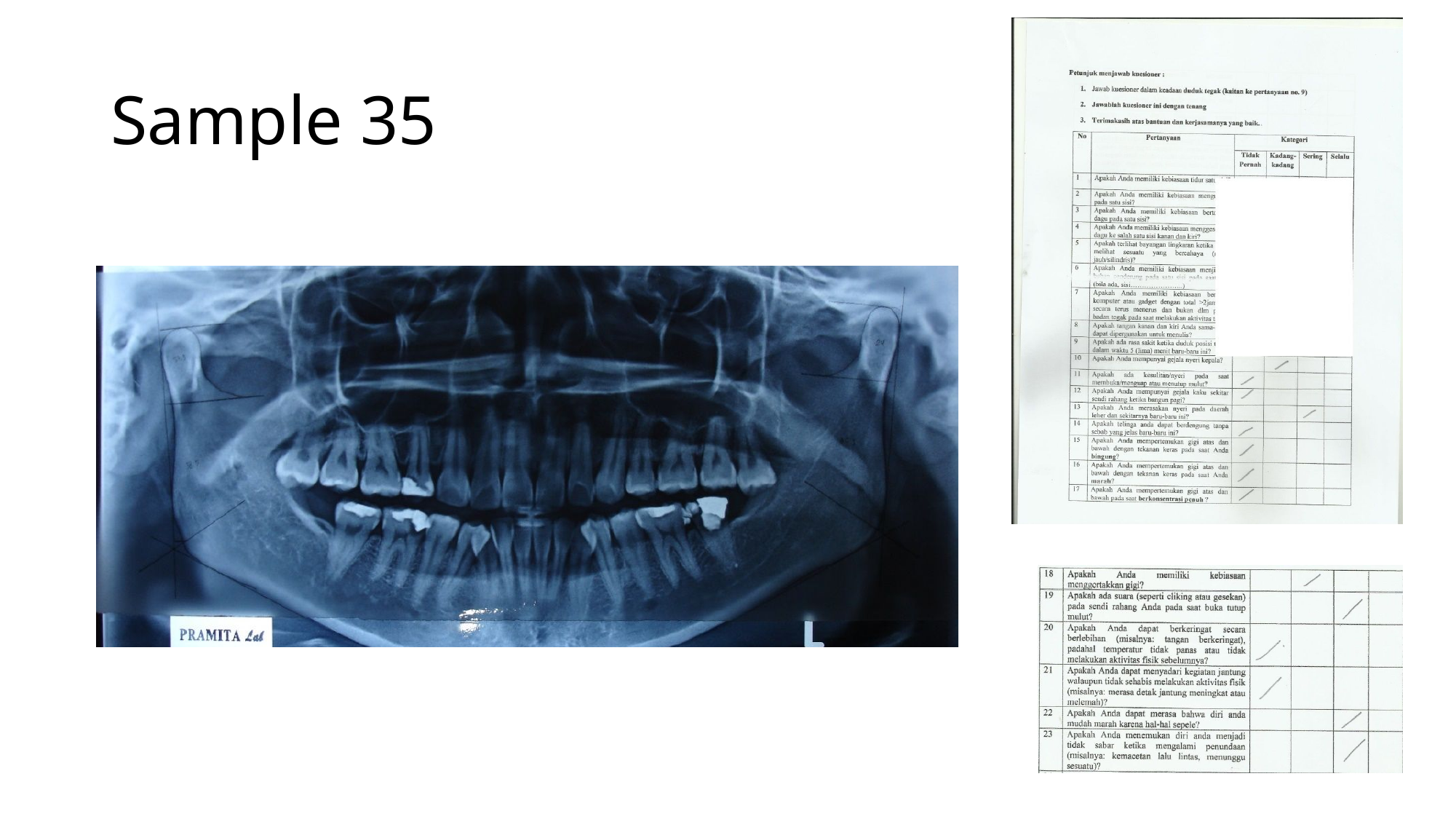

# Sample 35

## Slide 27
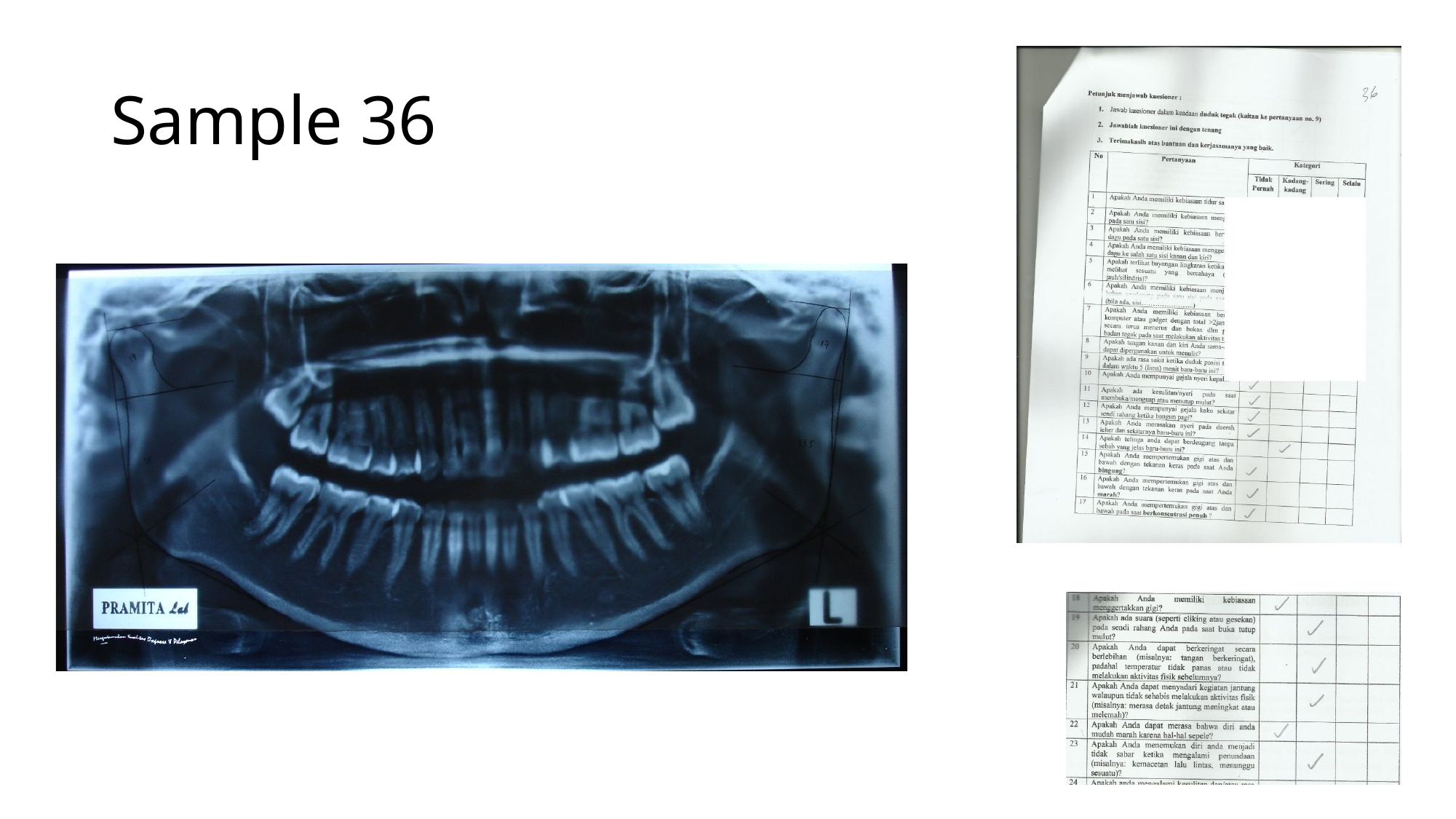

# Sample 36

## Slide 28
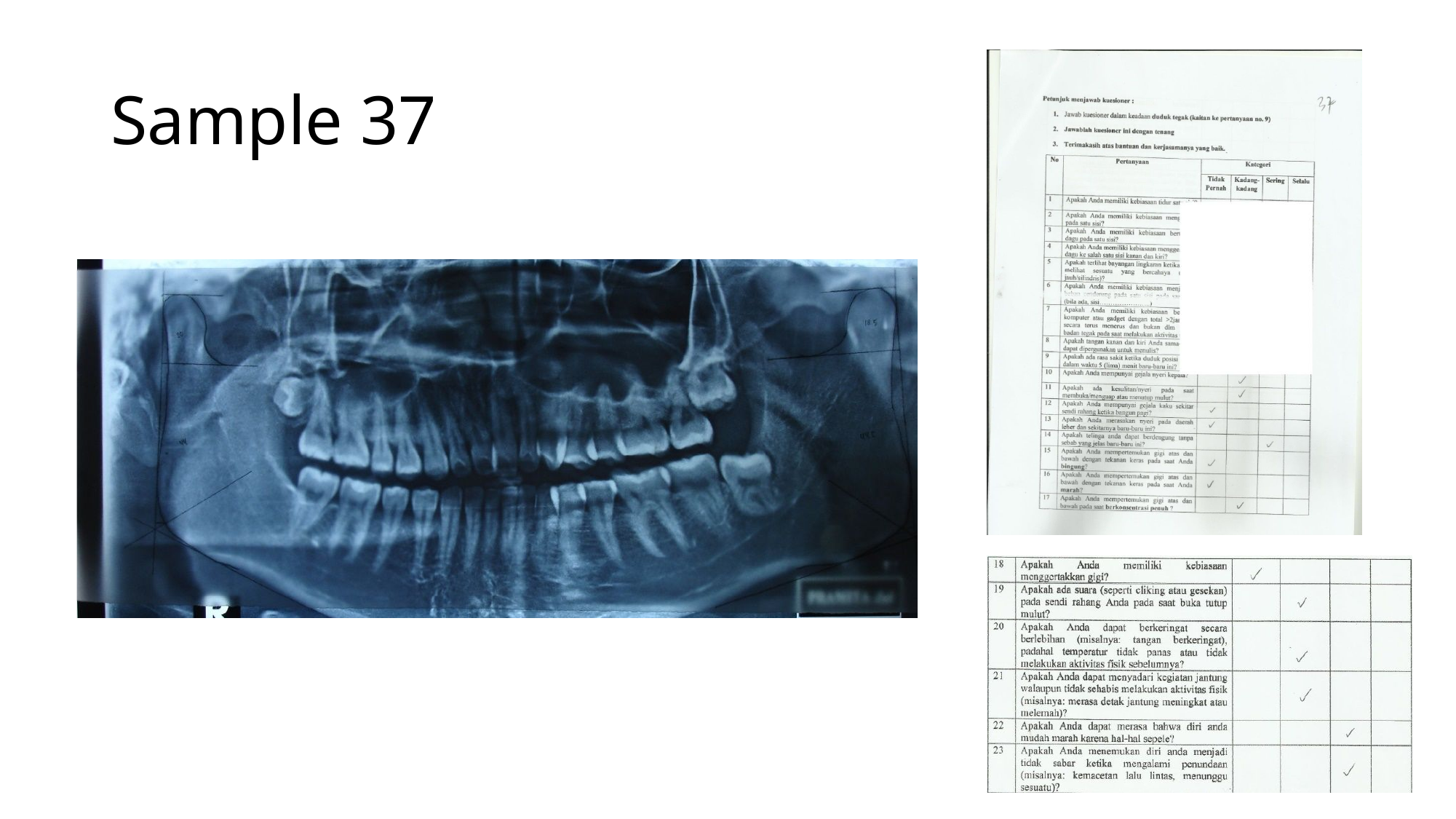

# Sample 37

## Slide 29
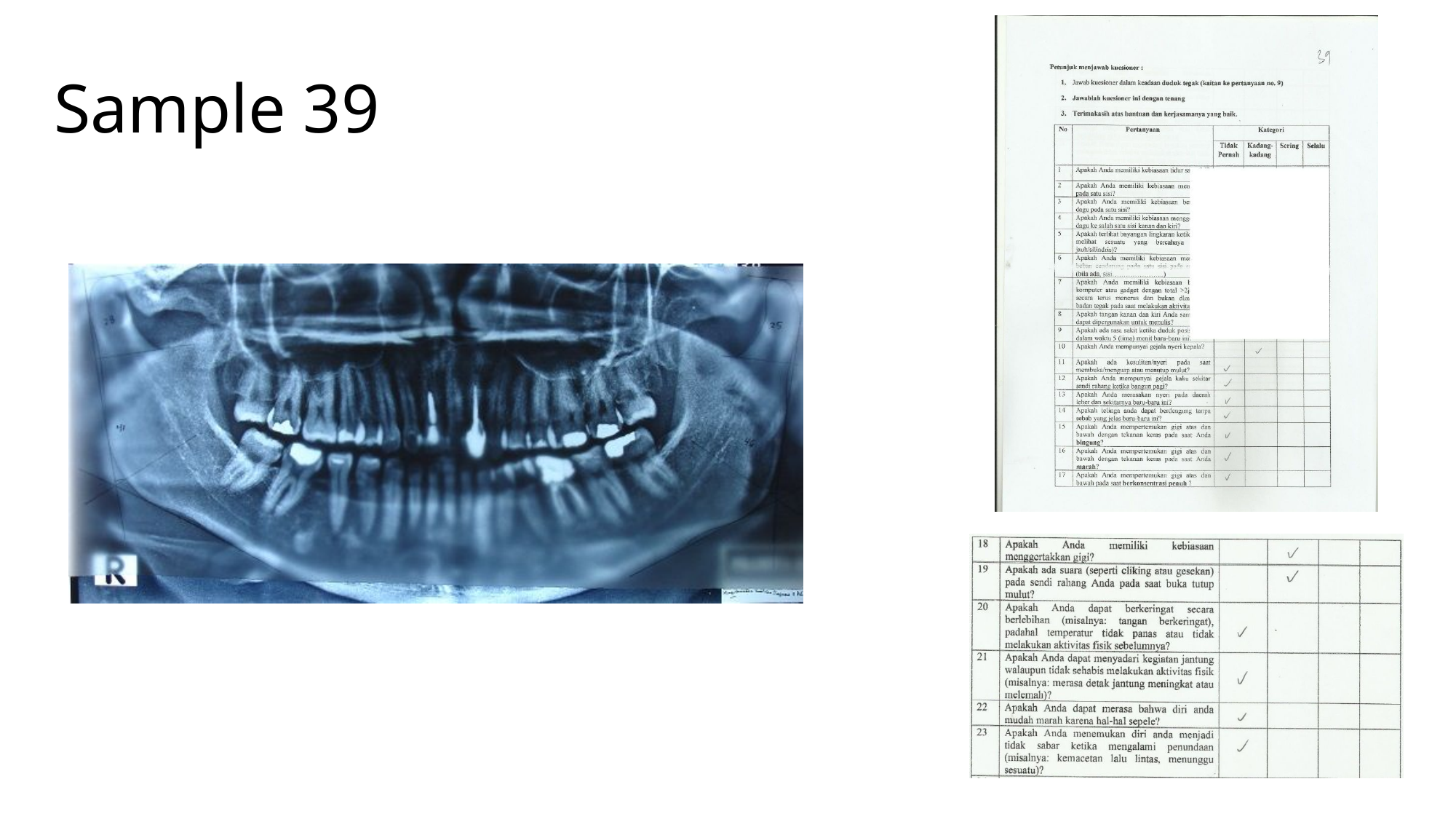

# Sample 39

## Slide 30
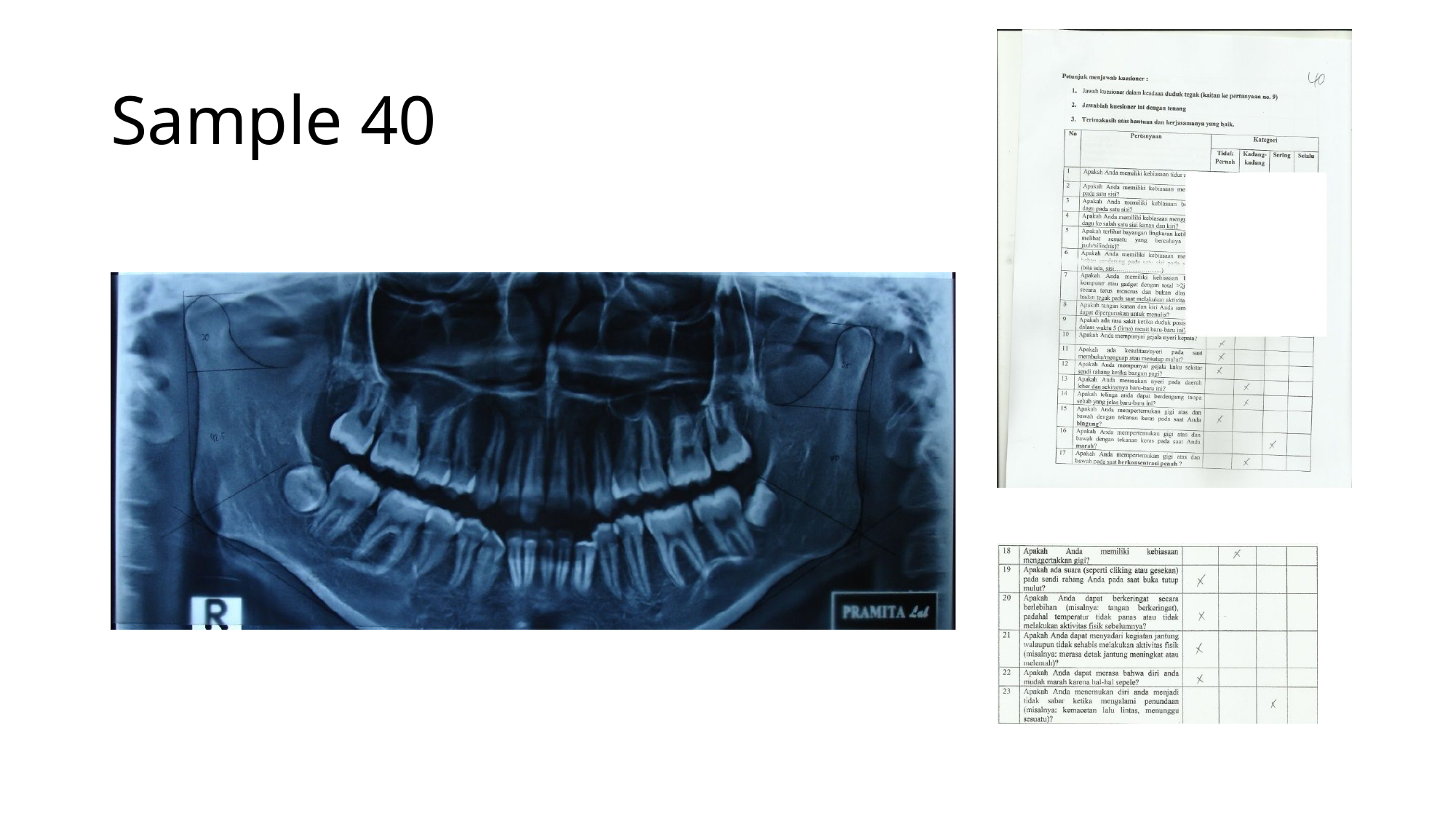

# Sample 40

## Slide 31
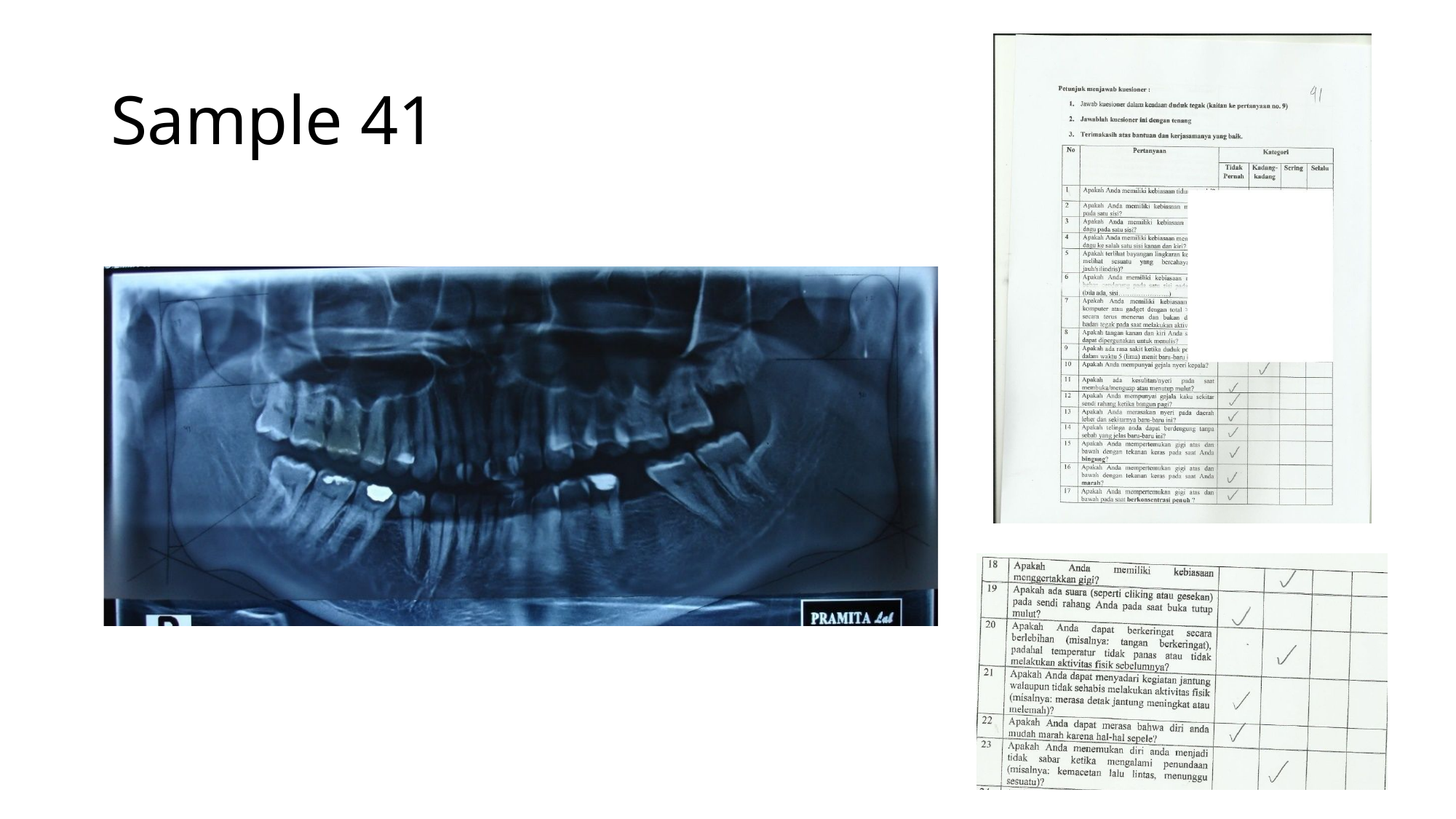

# Sample 41

## Slide 32
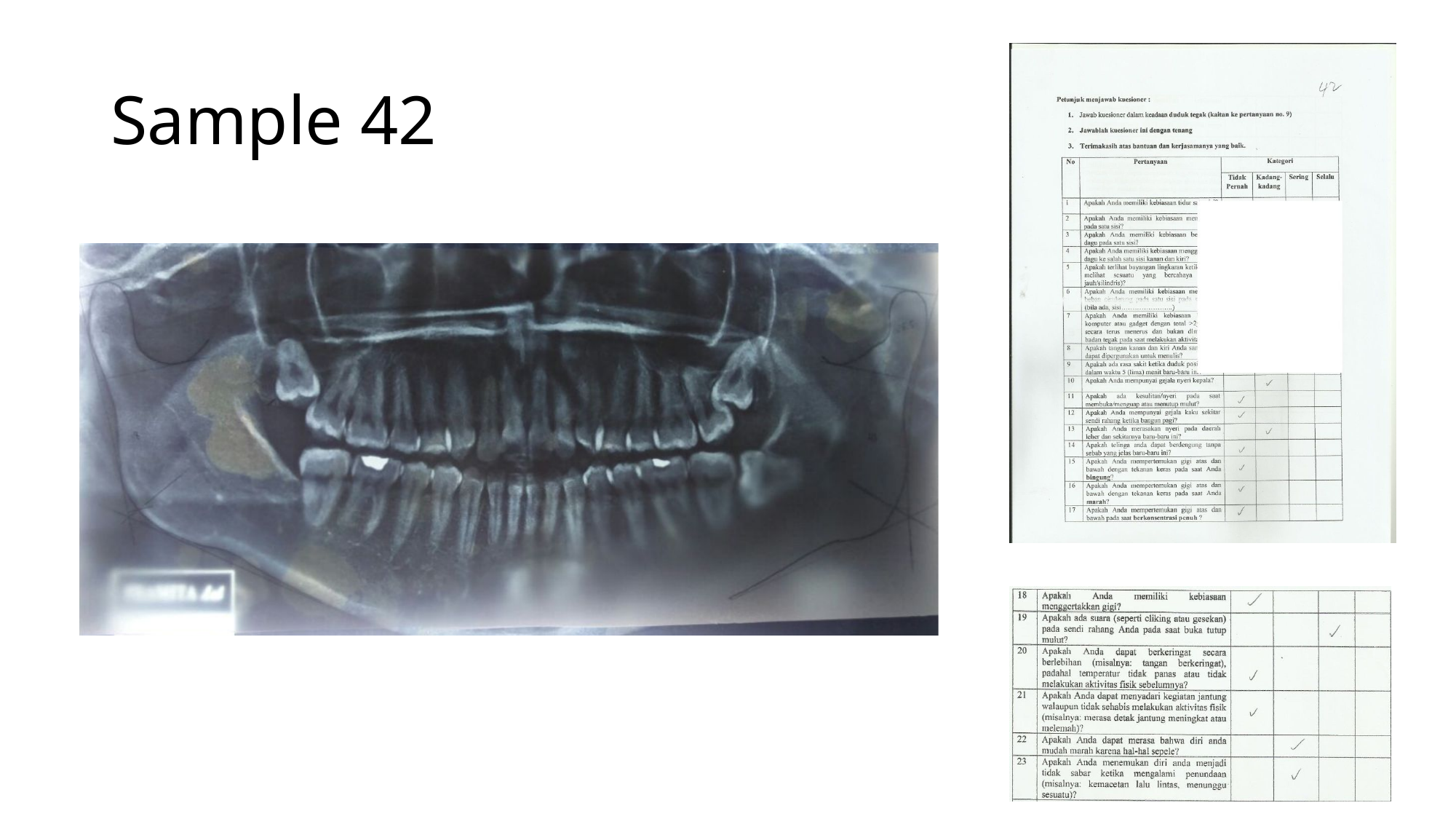

# Sample 42

## Slide 33
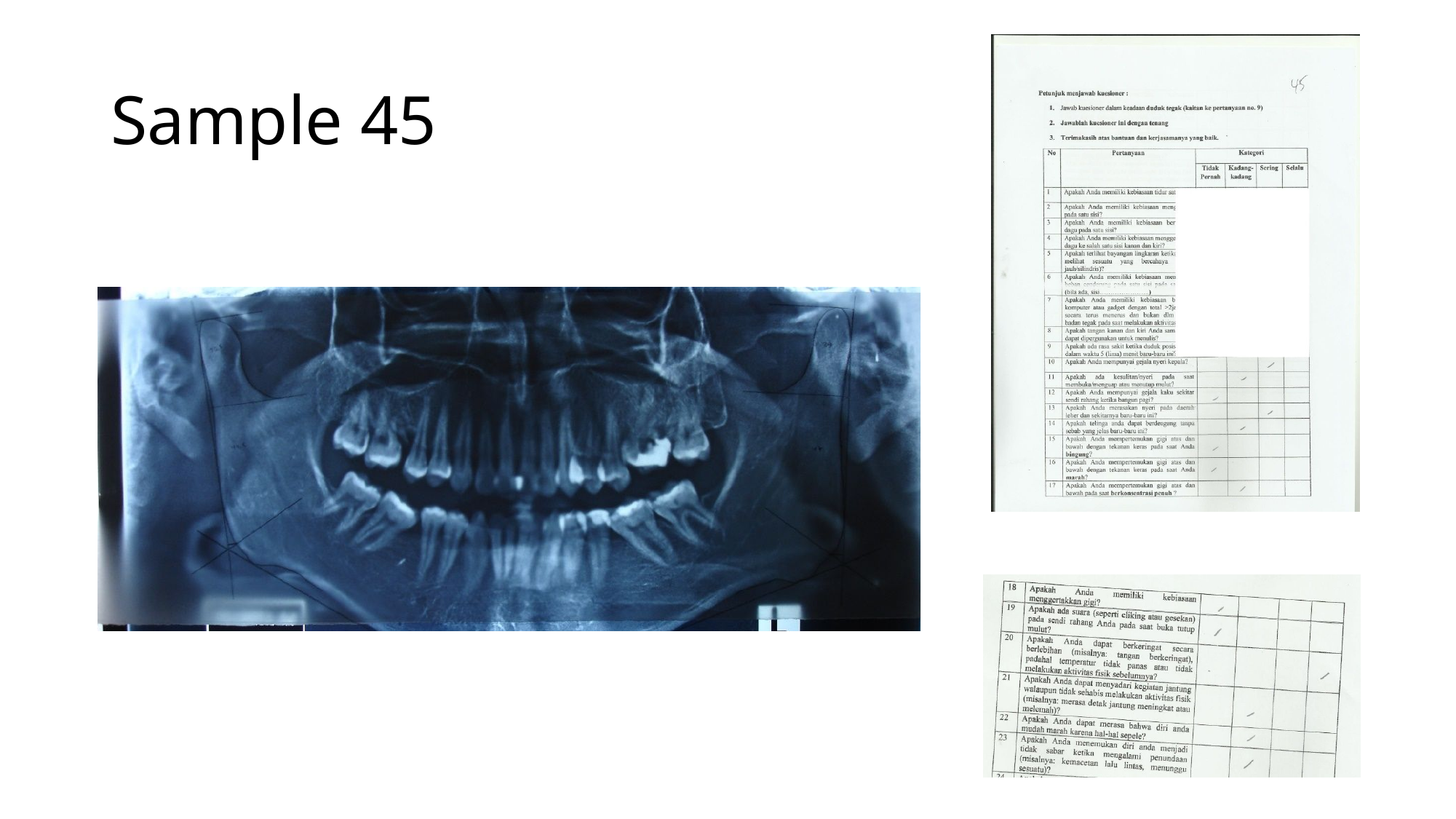

# Sample 45

## Slide 34
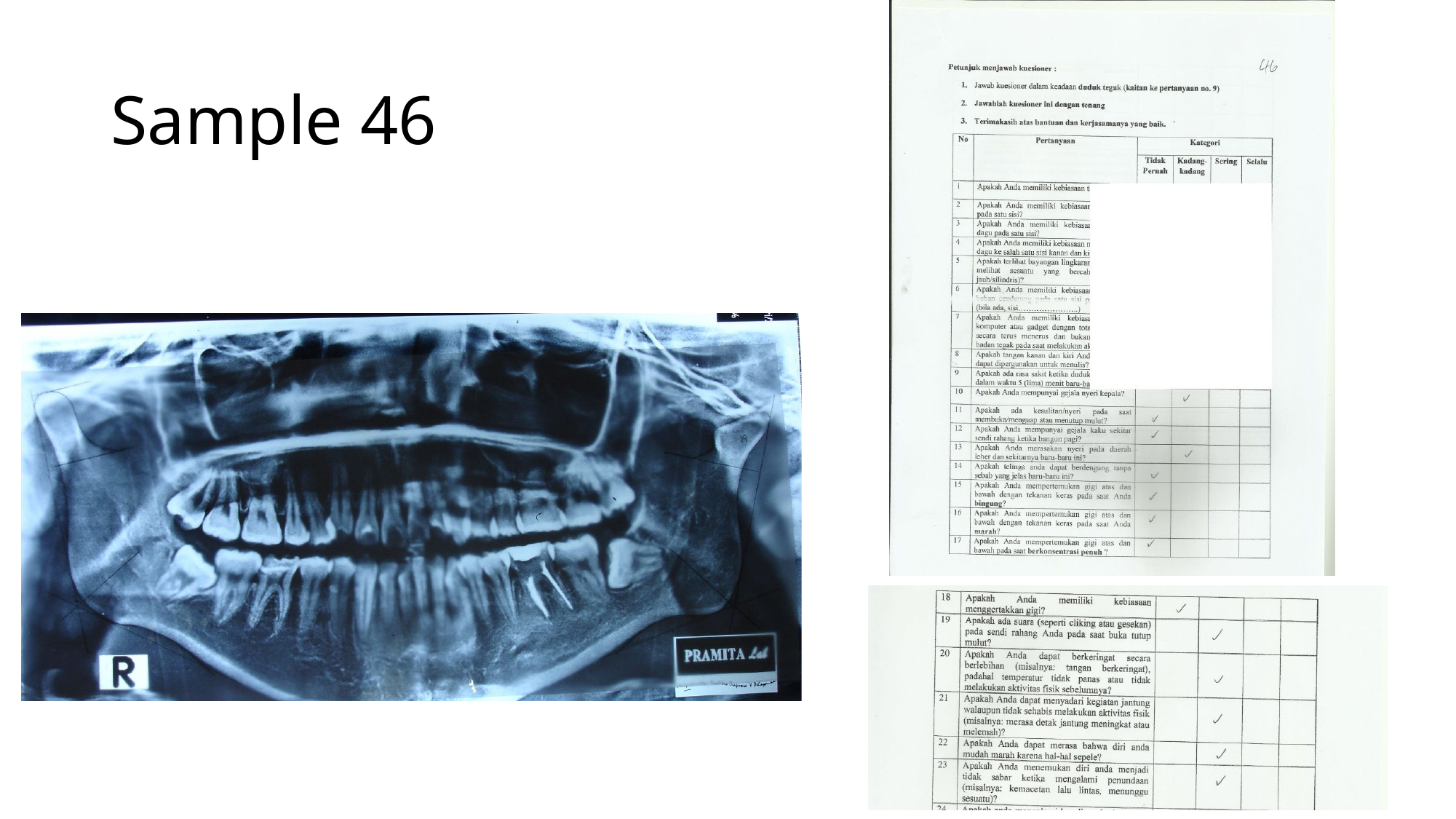

# Sample 46

## Slide 35
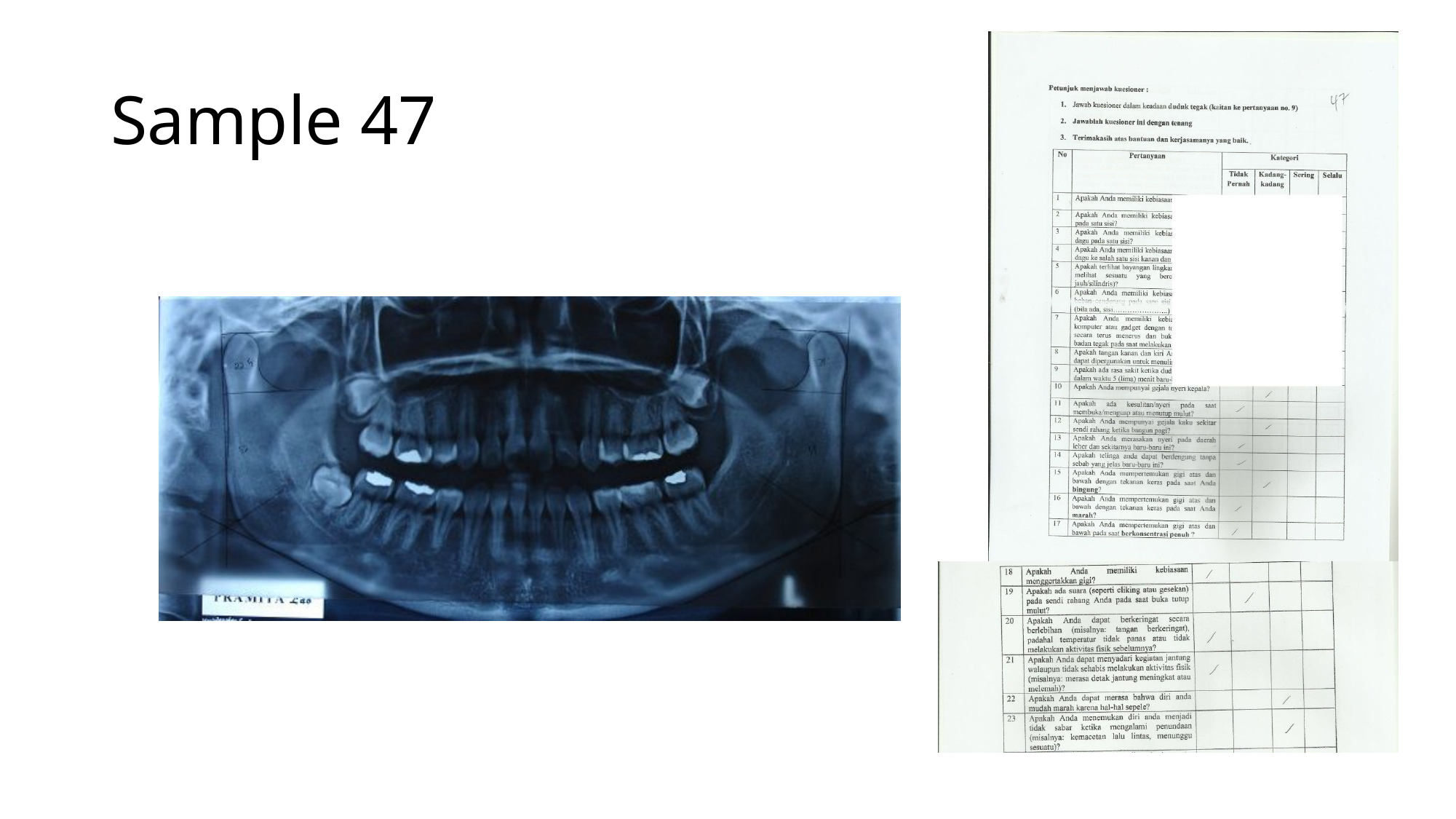

# Sample 47

## Slide 36
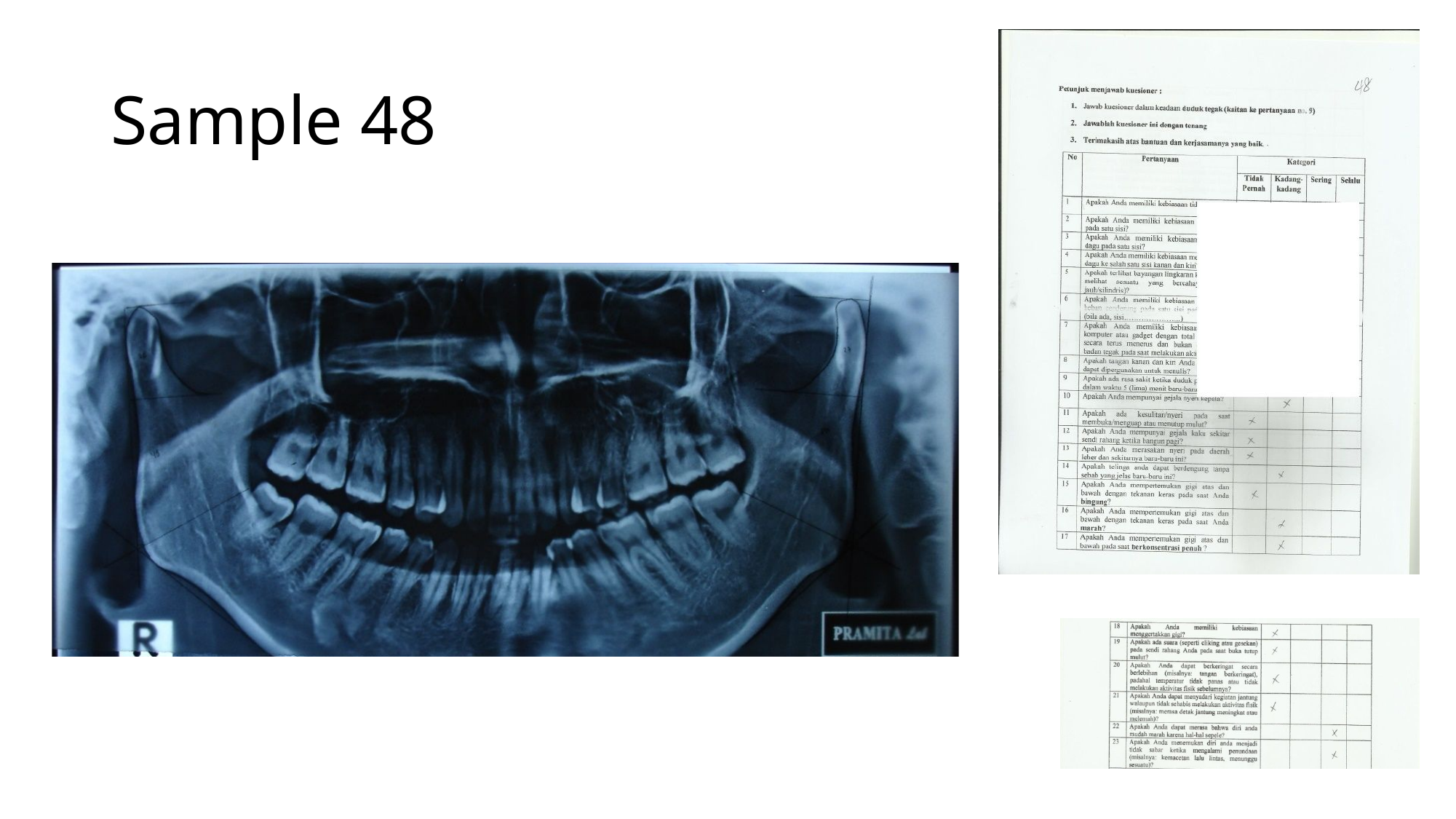

# Sample 48

## Slide 37
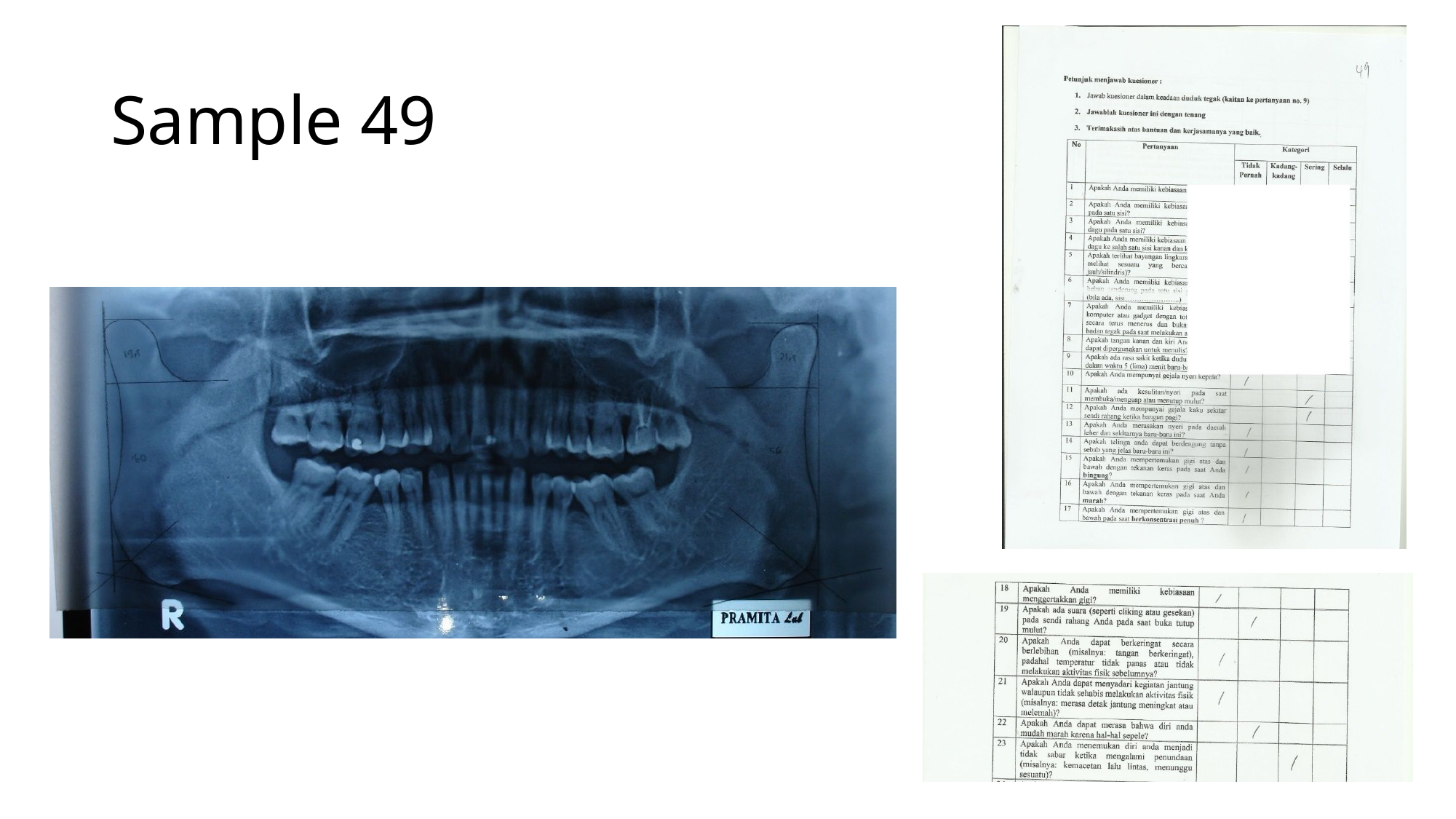

# Sample 49

## Slide 38
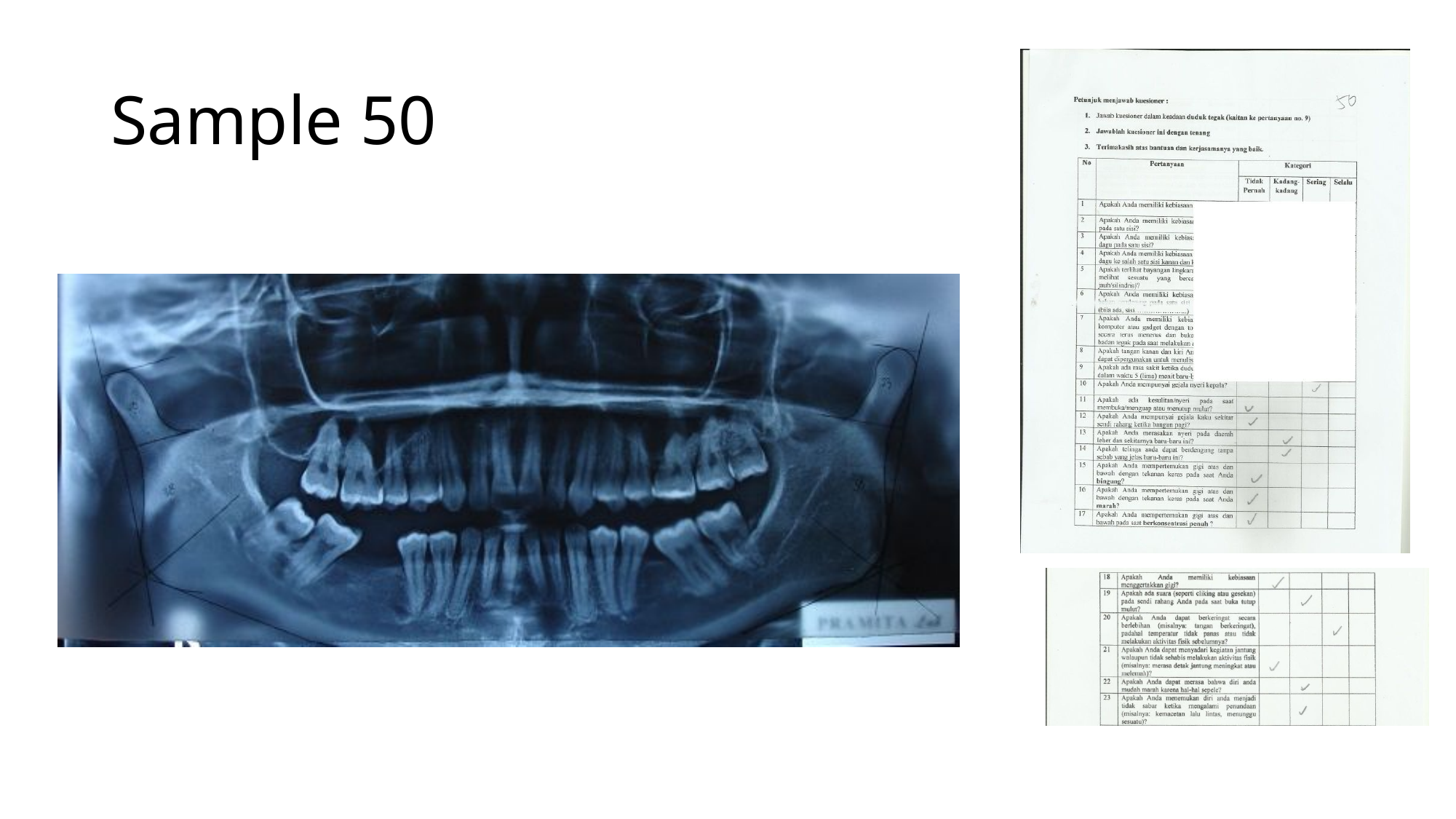

# Sample 50

## Slide 39
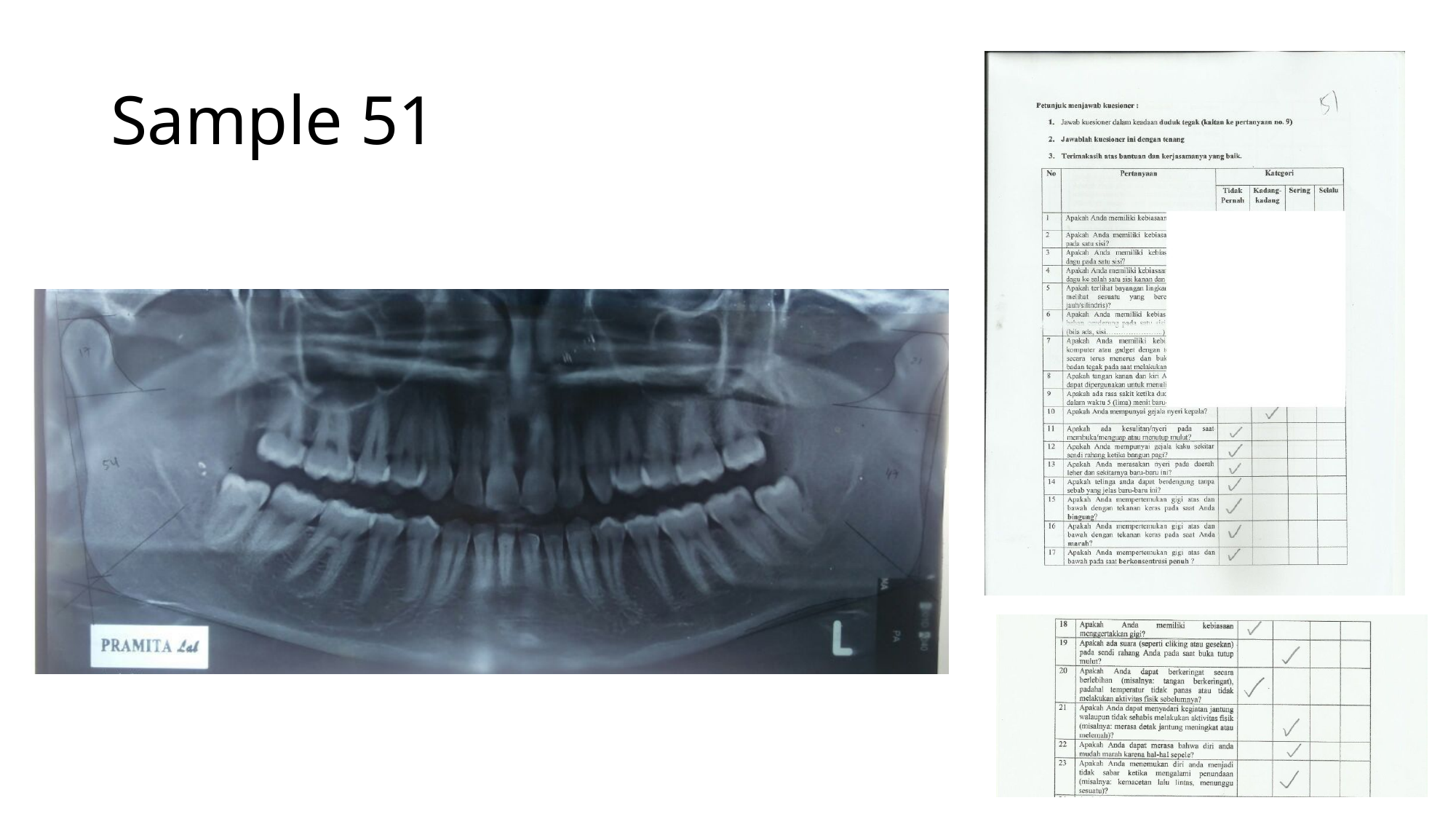

# Sample 51

## Slide 40
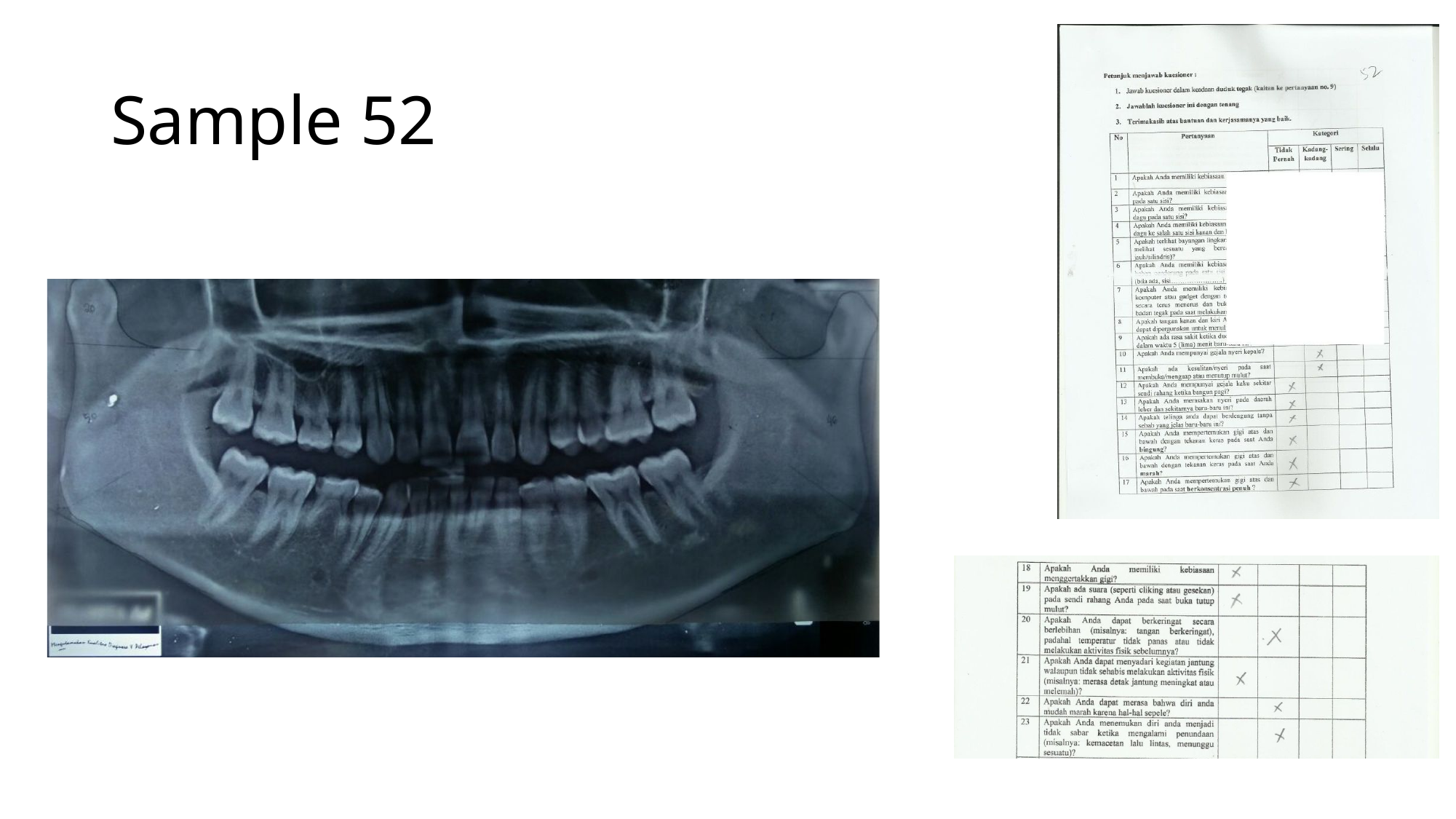

# Sample 52

## Slide 41
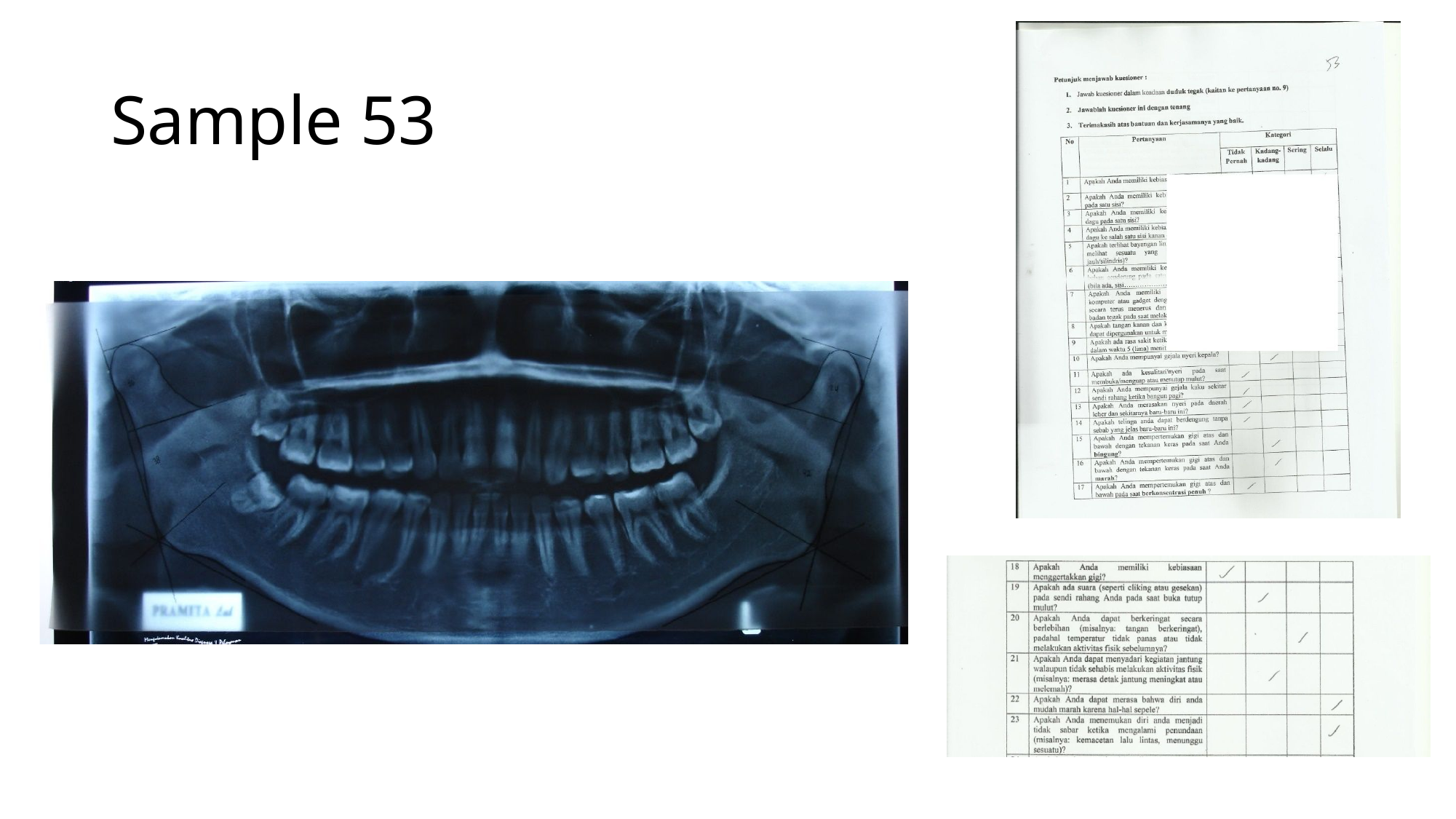

# Sample 53

## Slide 42
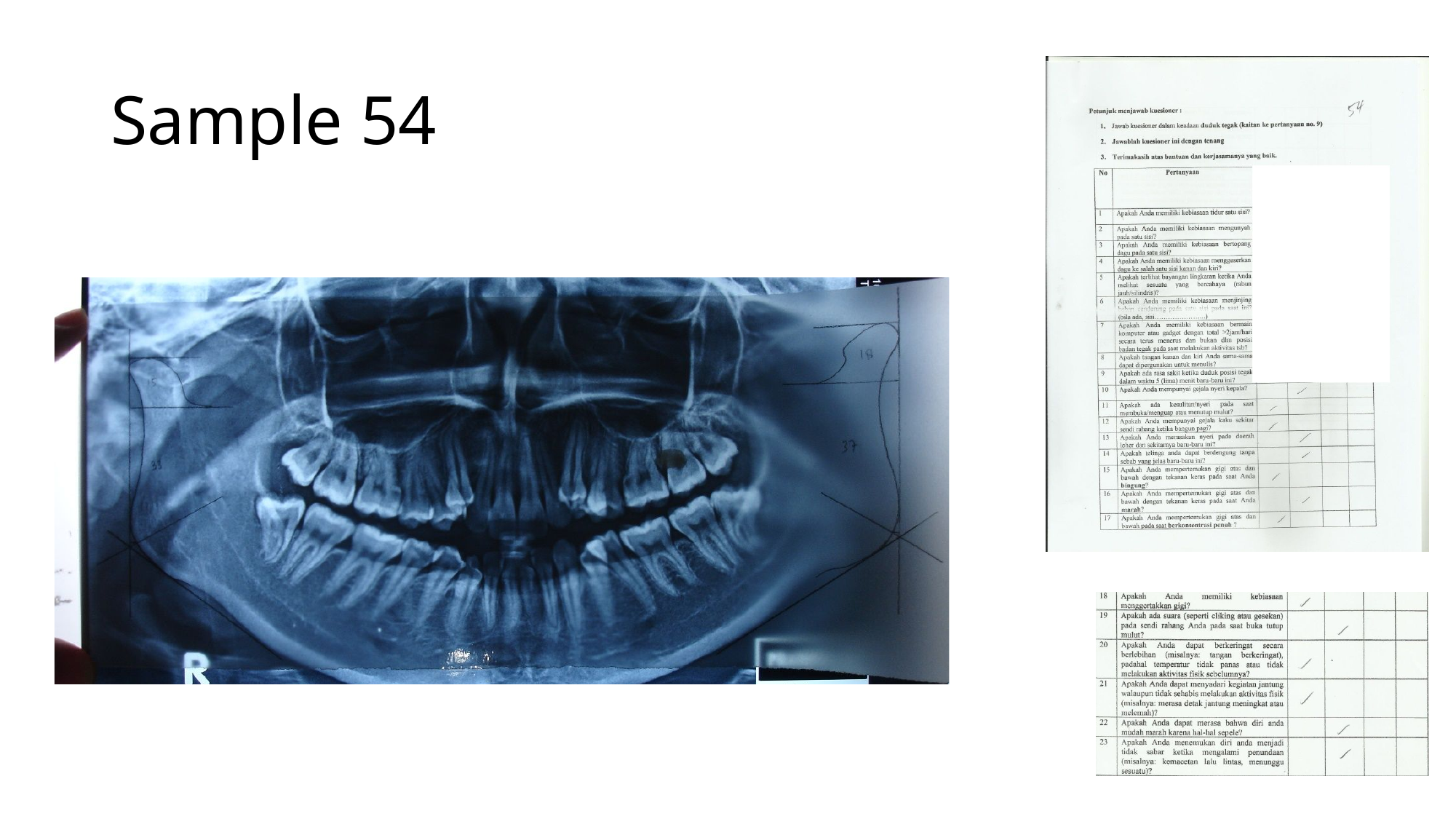

# Sample 54

## Slide 43
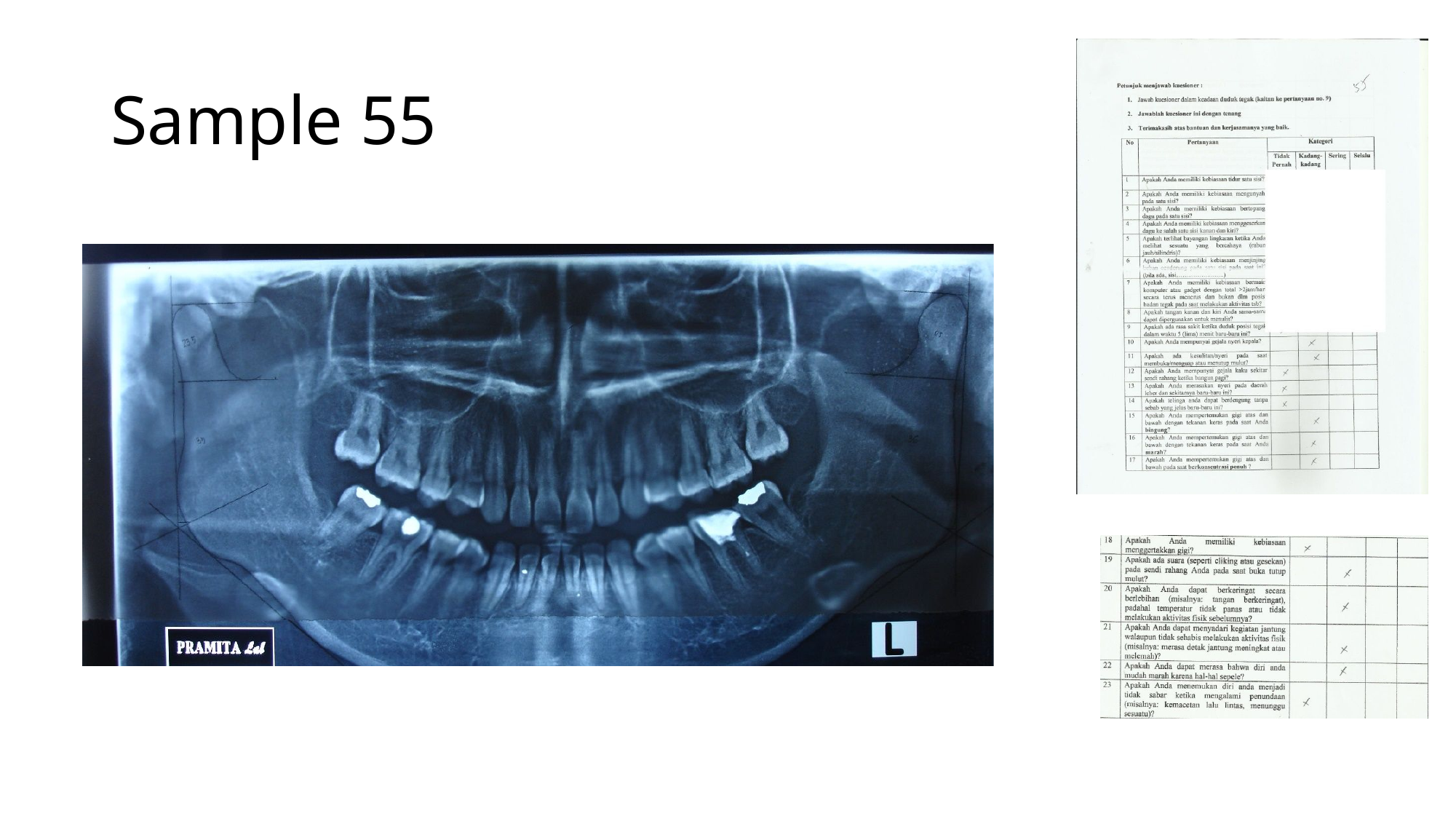

# Sample 55

## Slide 44
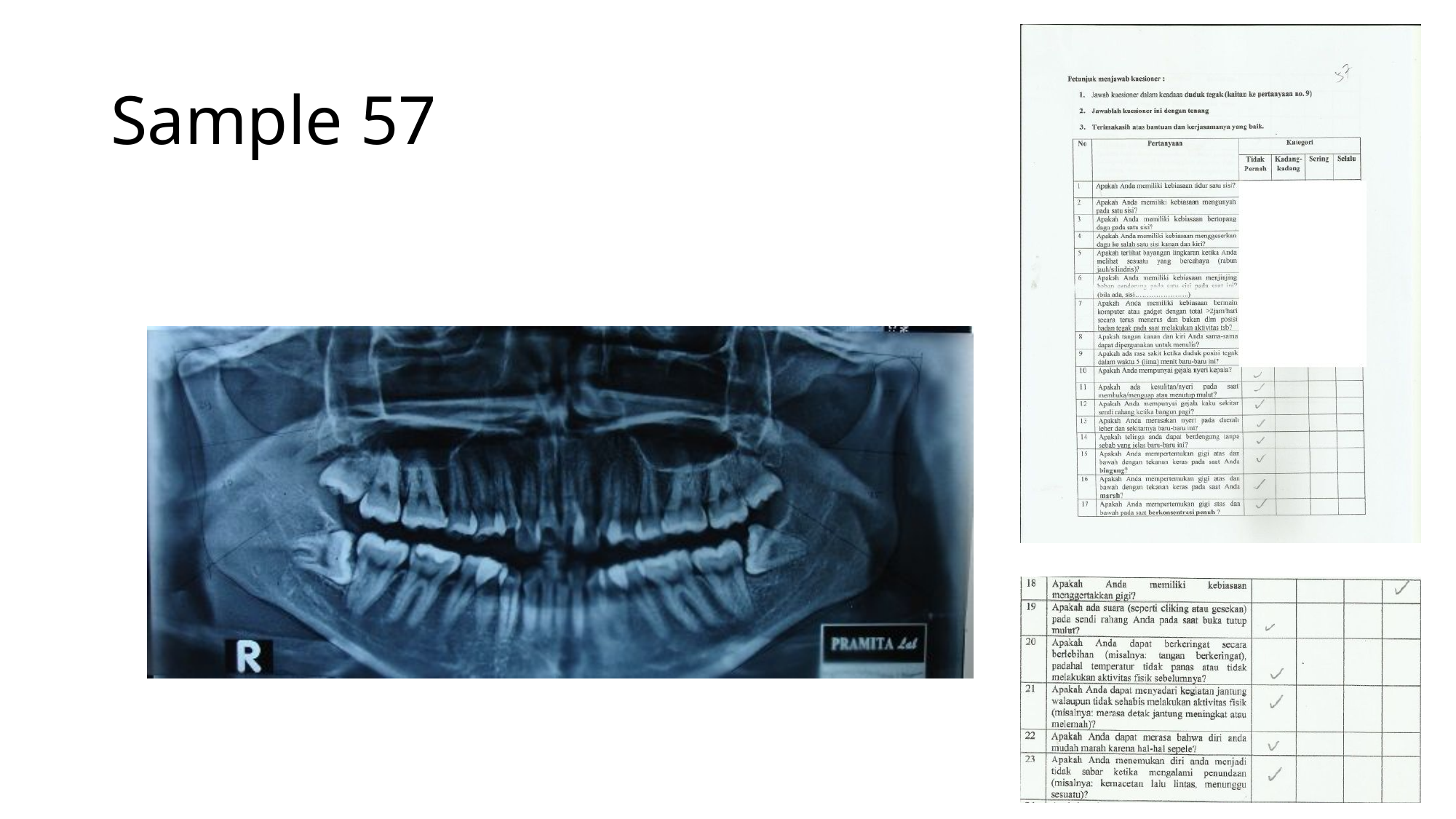

# Sample 57

## Slide 45
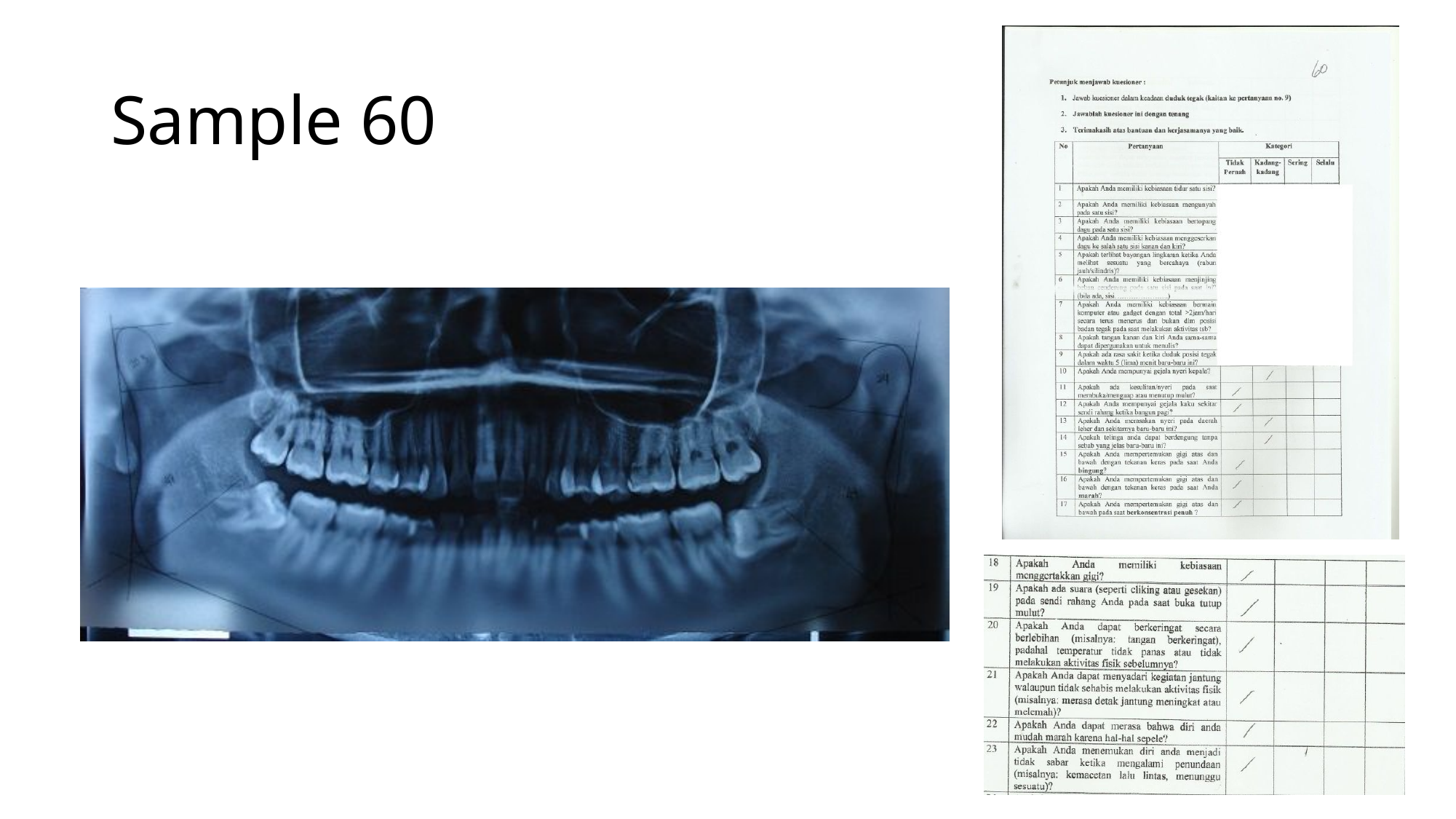

# Sample 60

## Slide 46
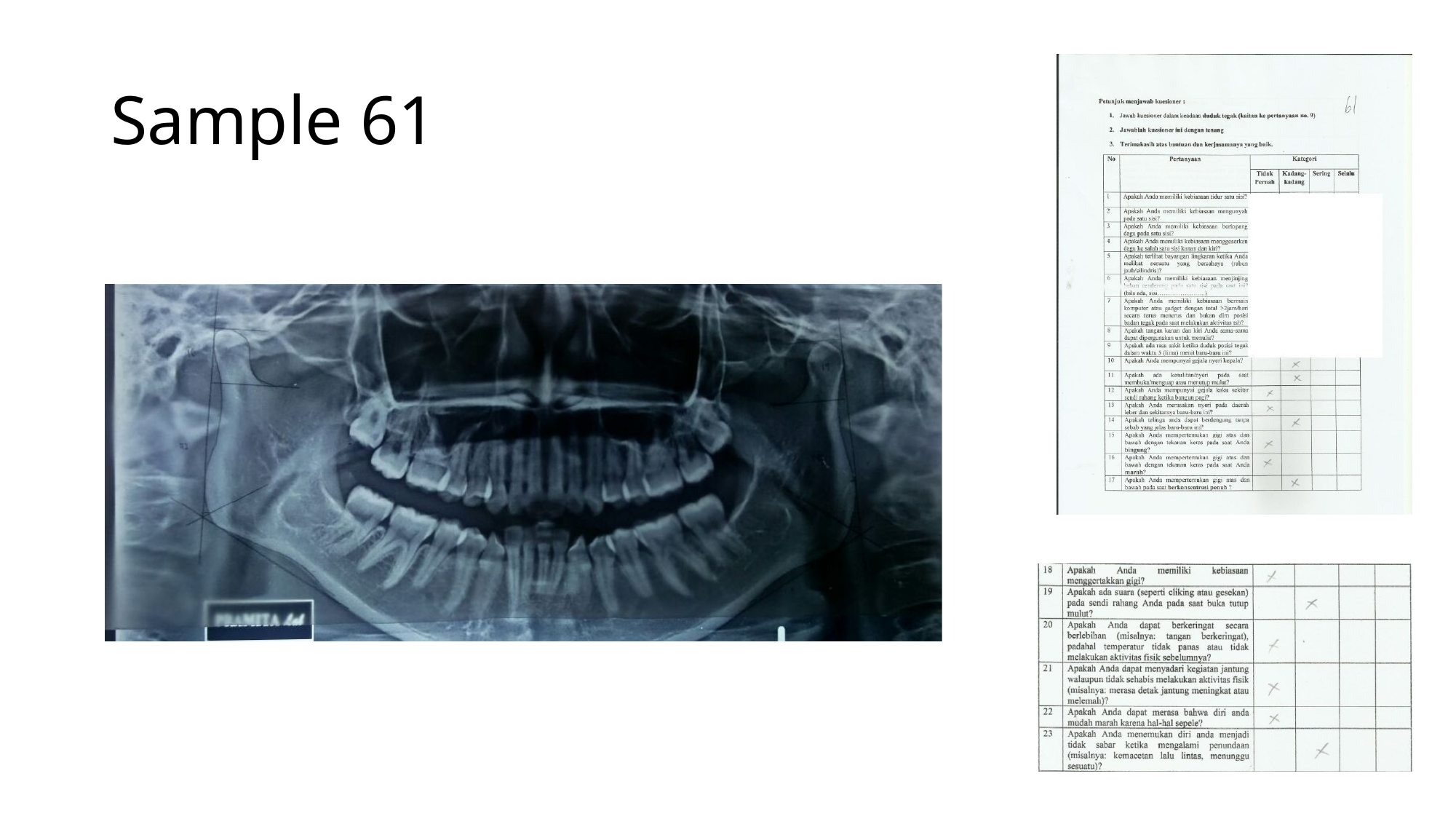

# Sample 61

## Slide 47
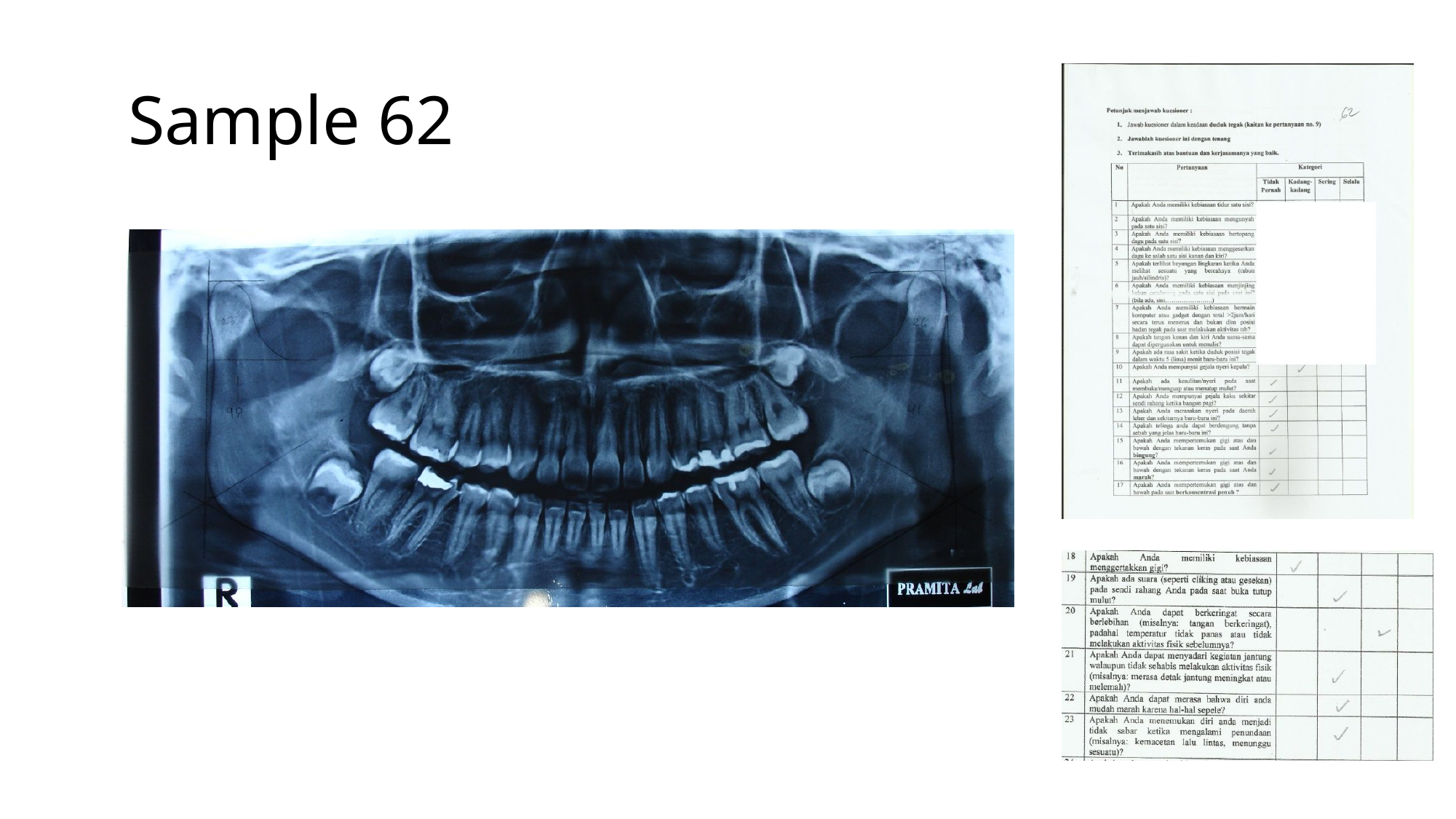

# Sample 62

## Slide 48
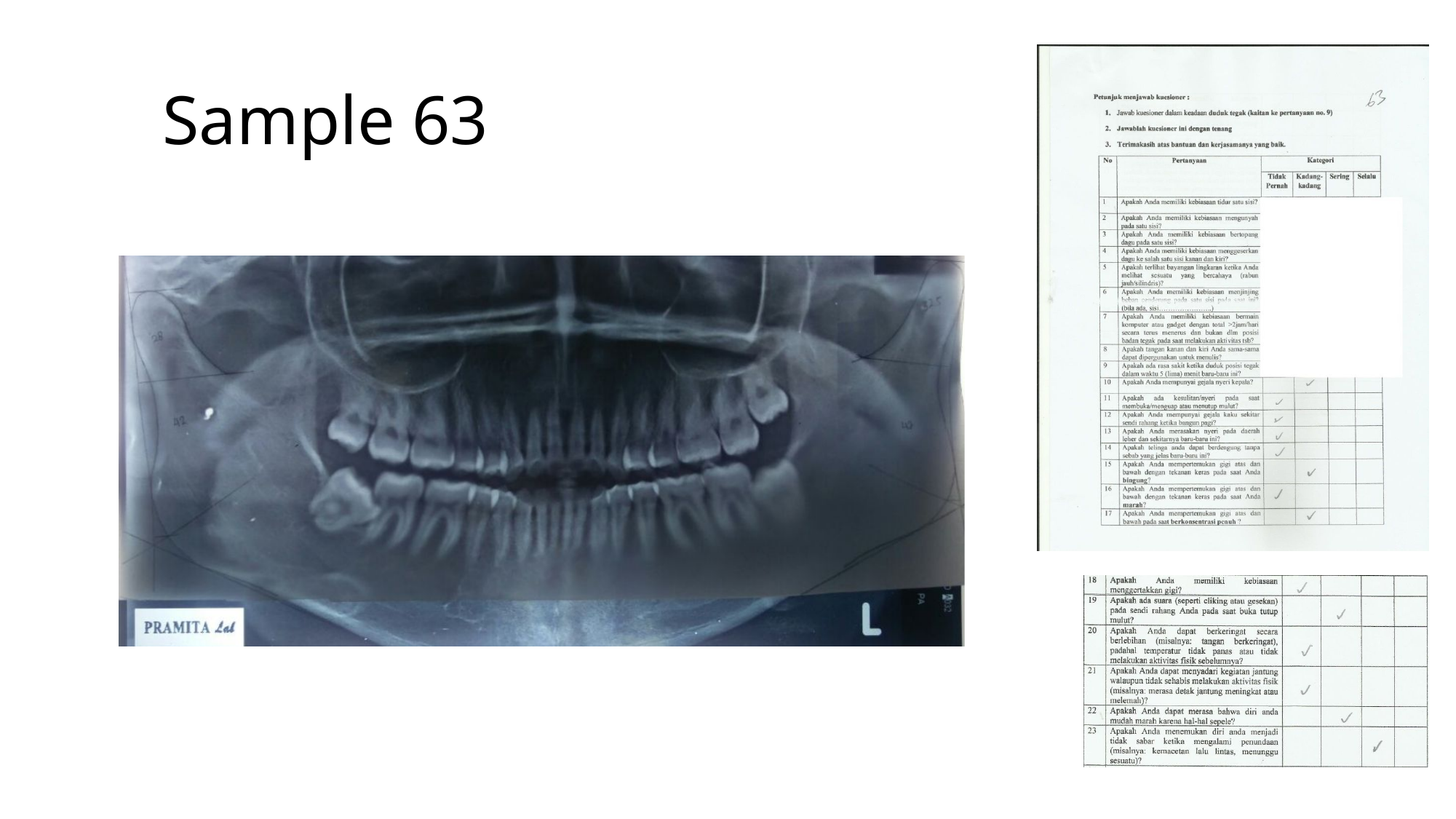

# Sample 63

## Slide 49
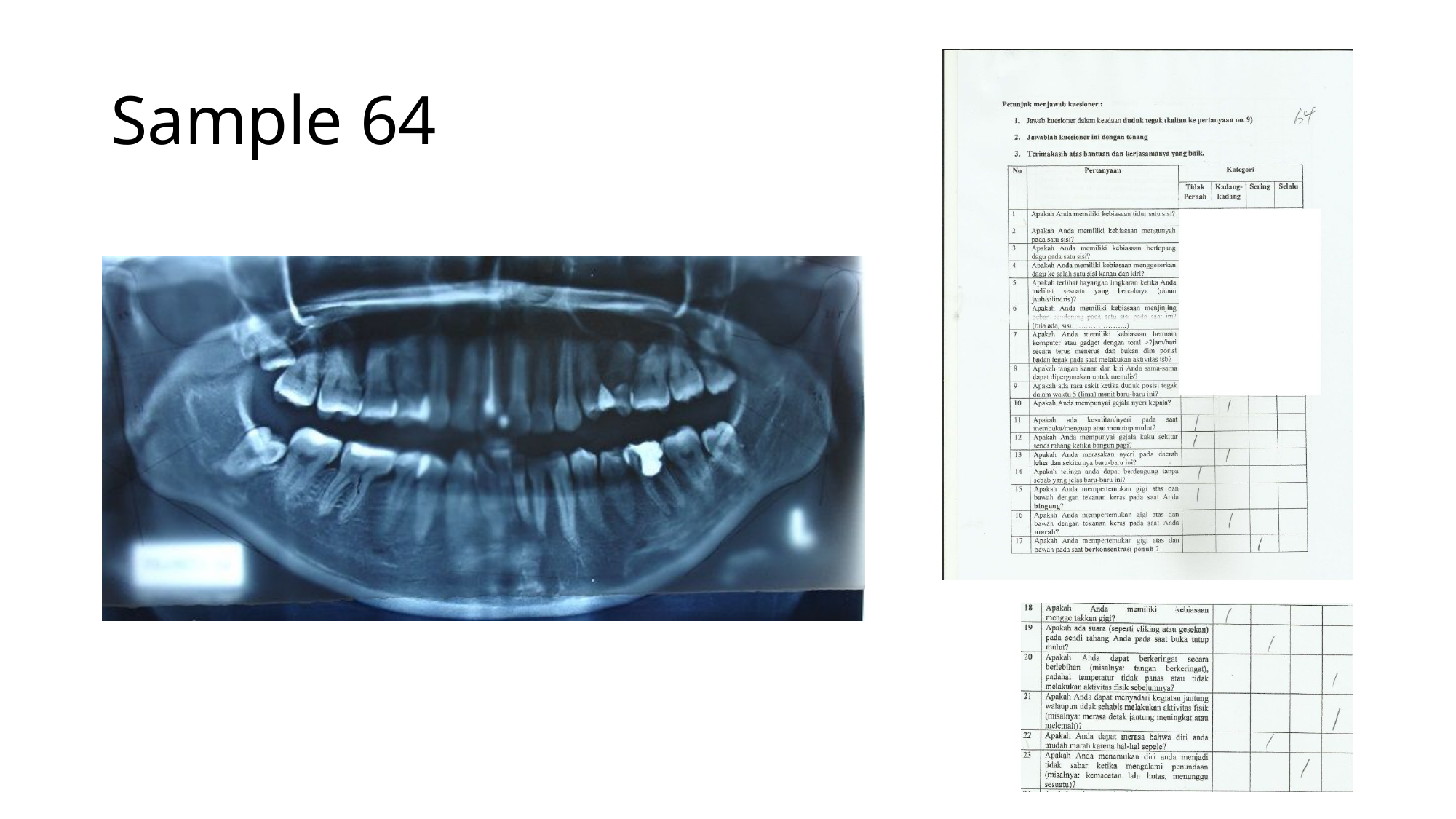

# Sample 64

## Slide 50
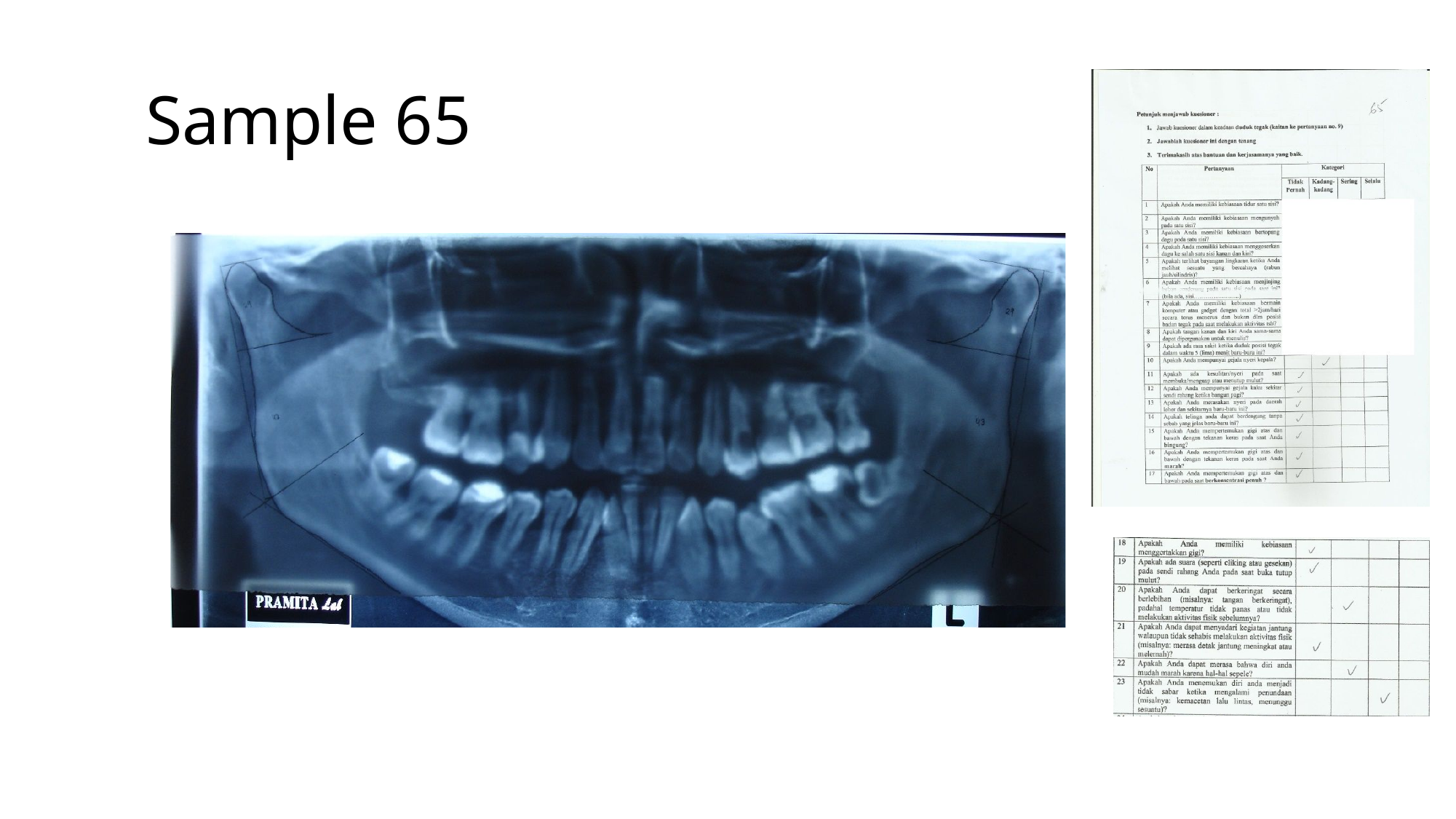

# Sample 65

## Slide 51
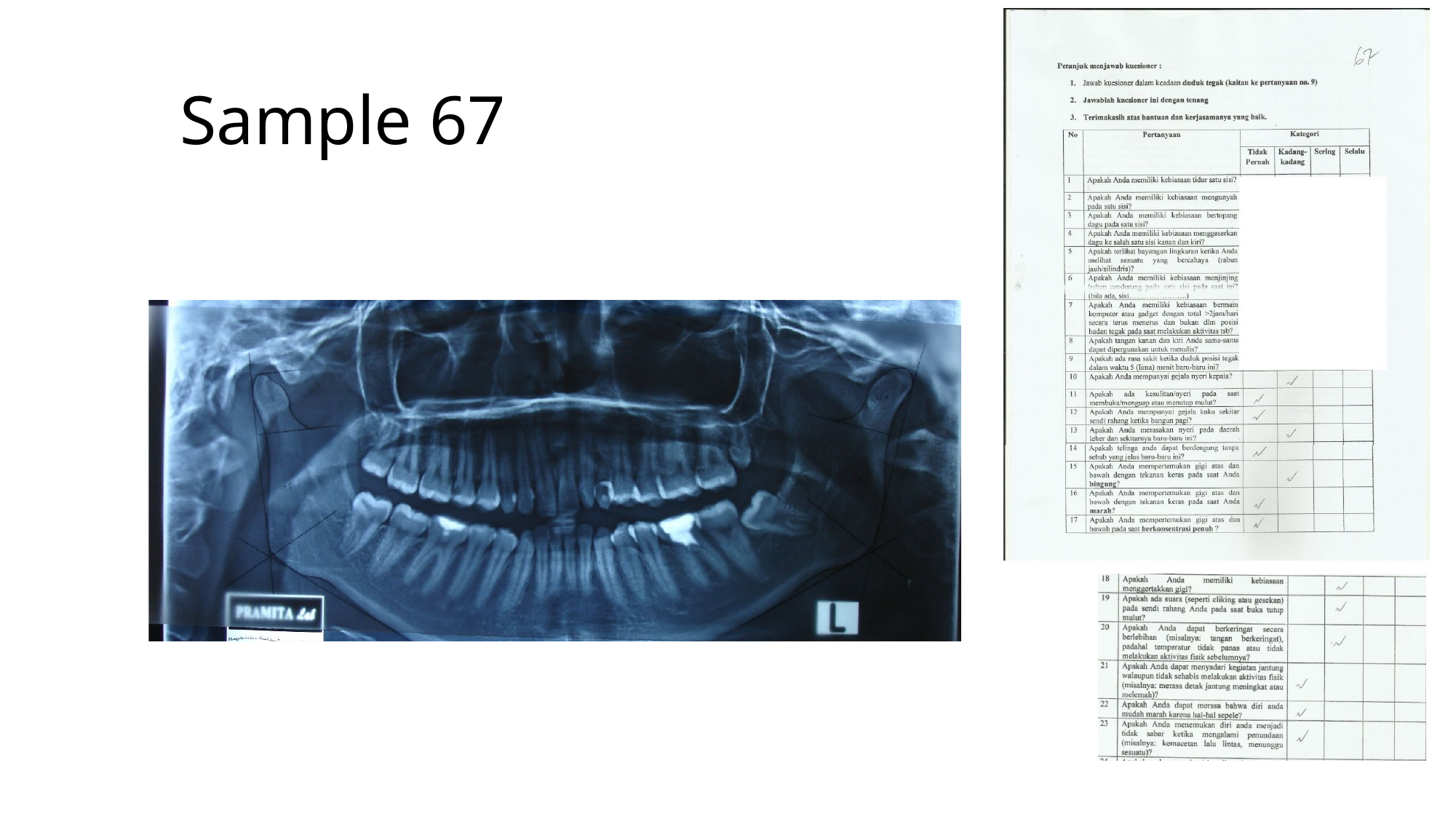

# Sample 67

## Slide 52
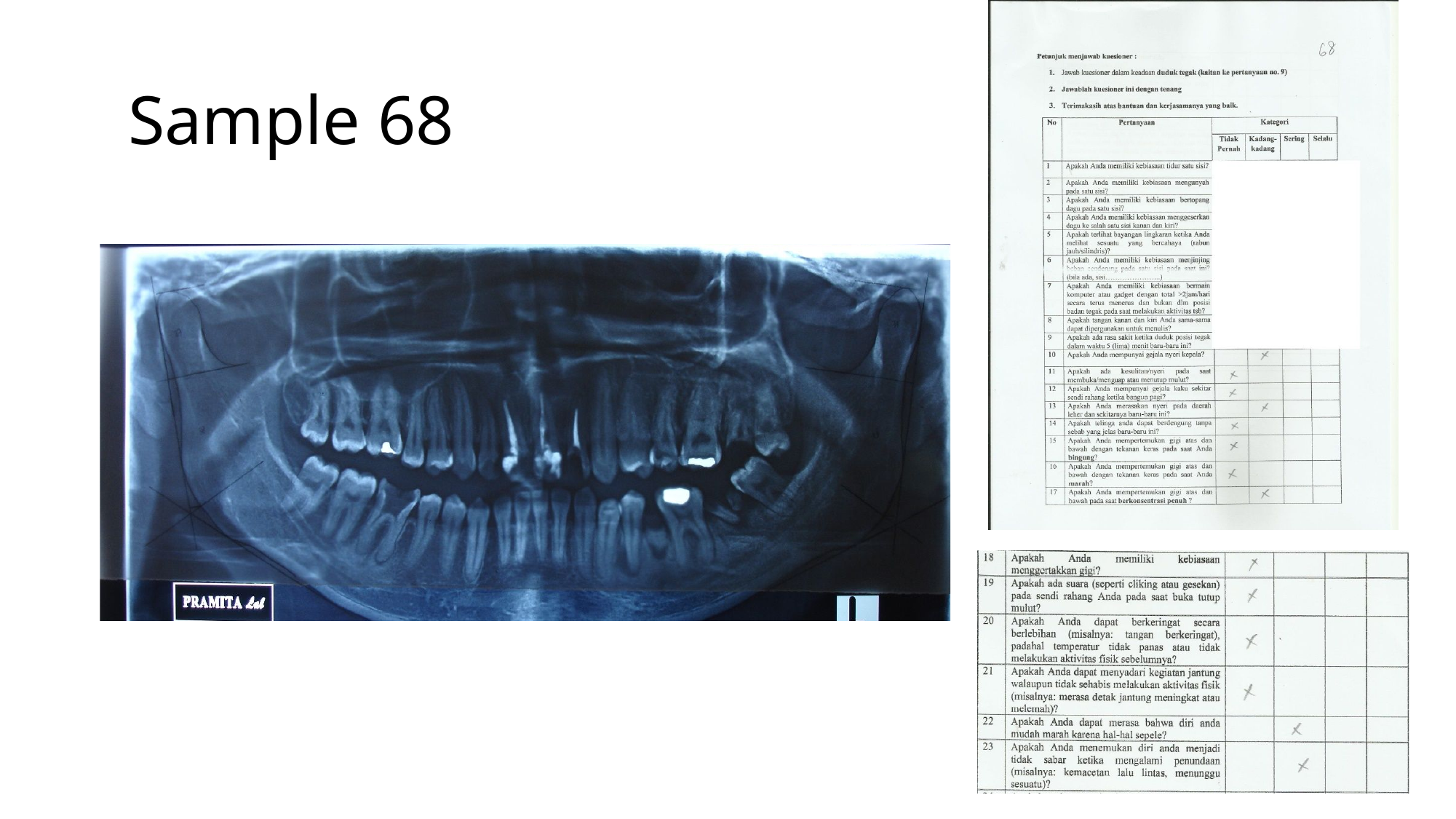

# Sample 68

## Slide 53
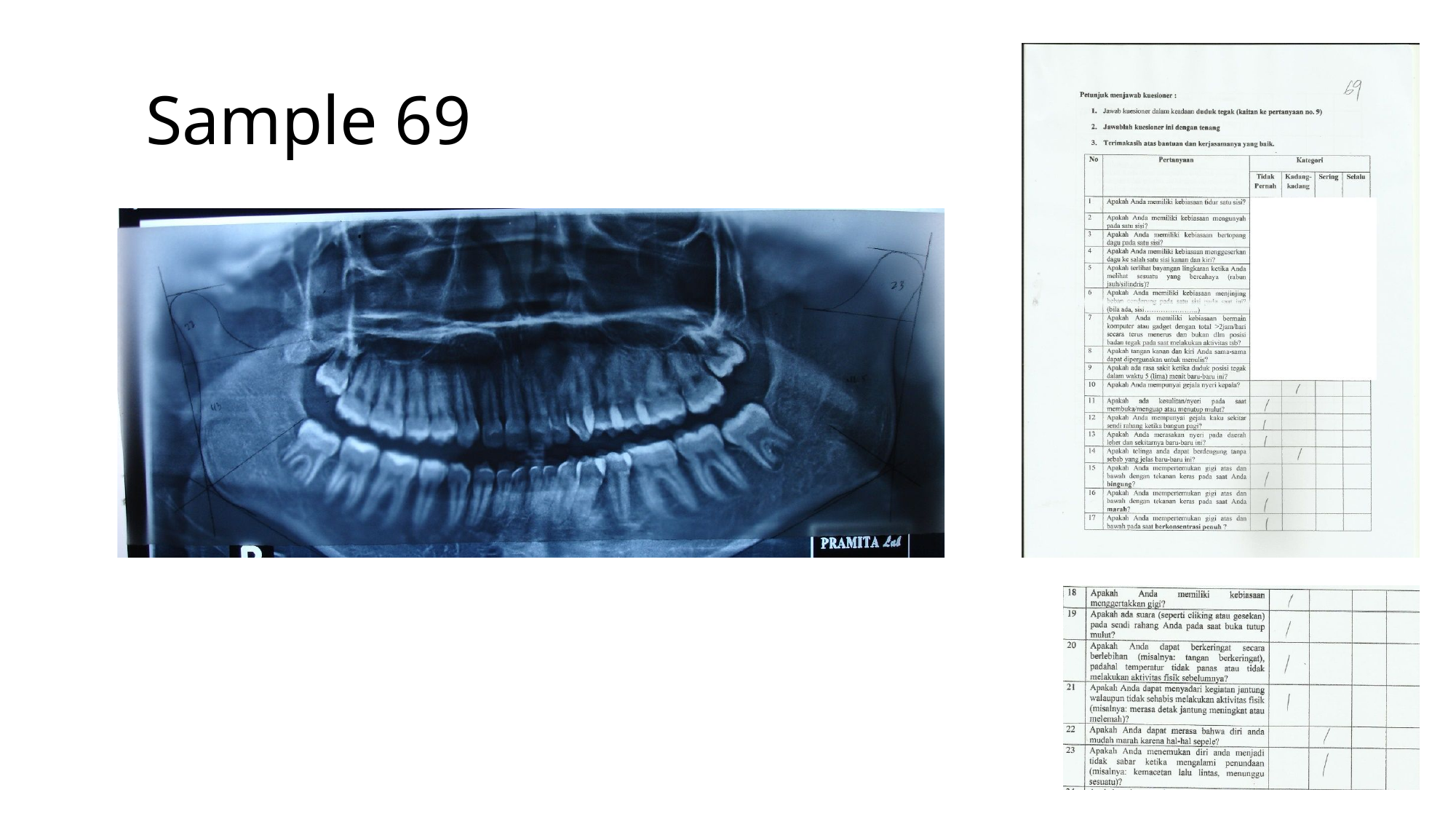

# Sample 69

## Slide 54
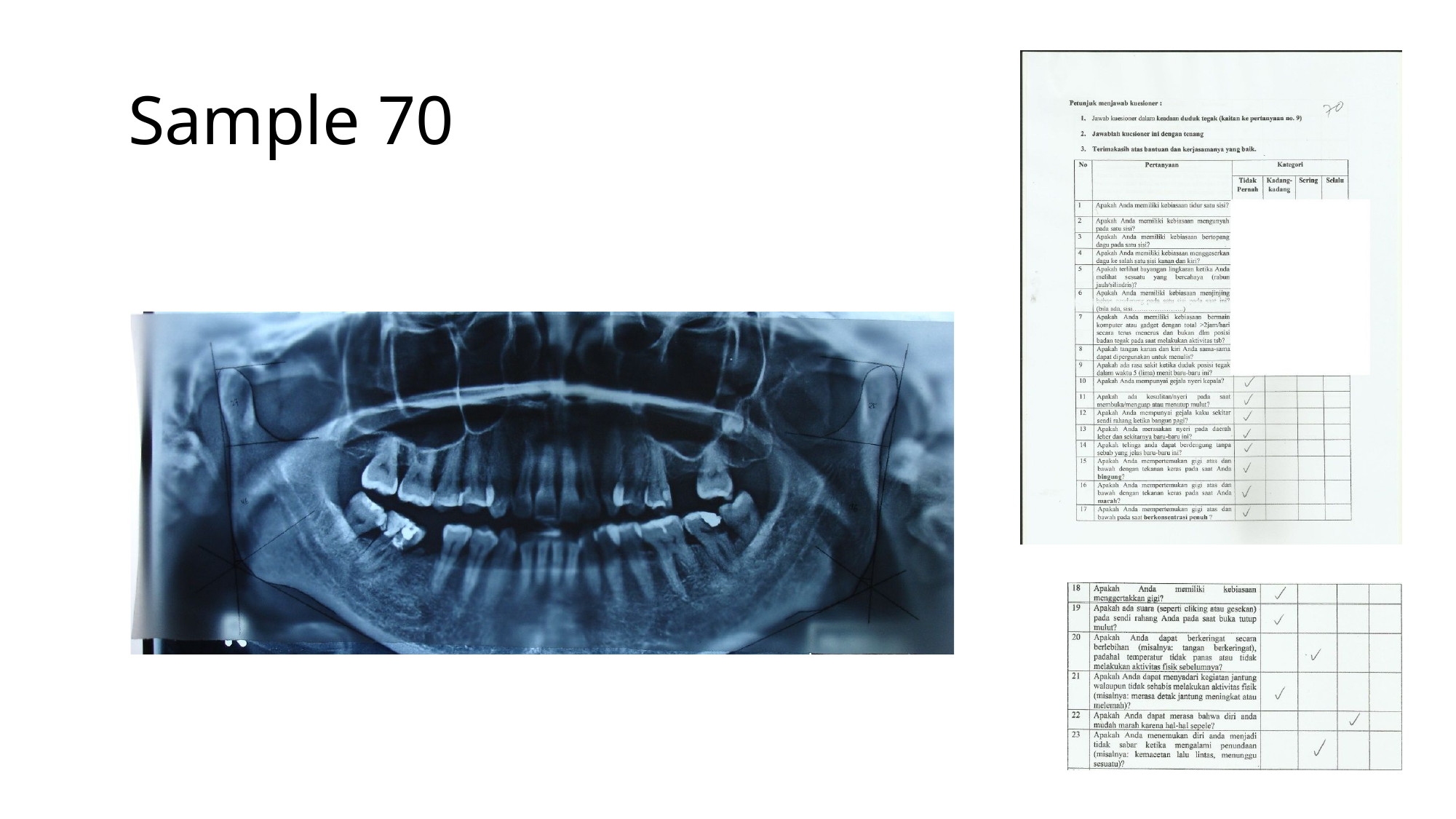

# Sample 70

## Slide 55
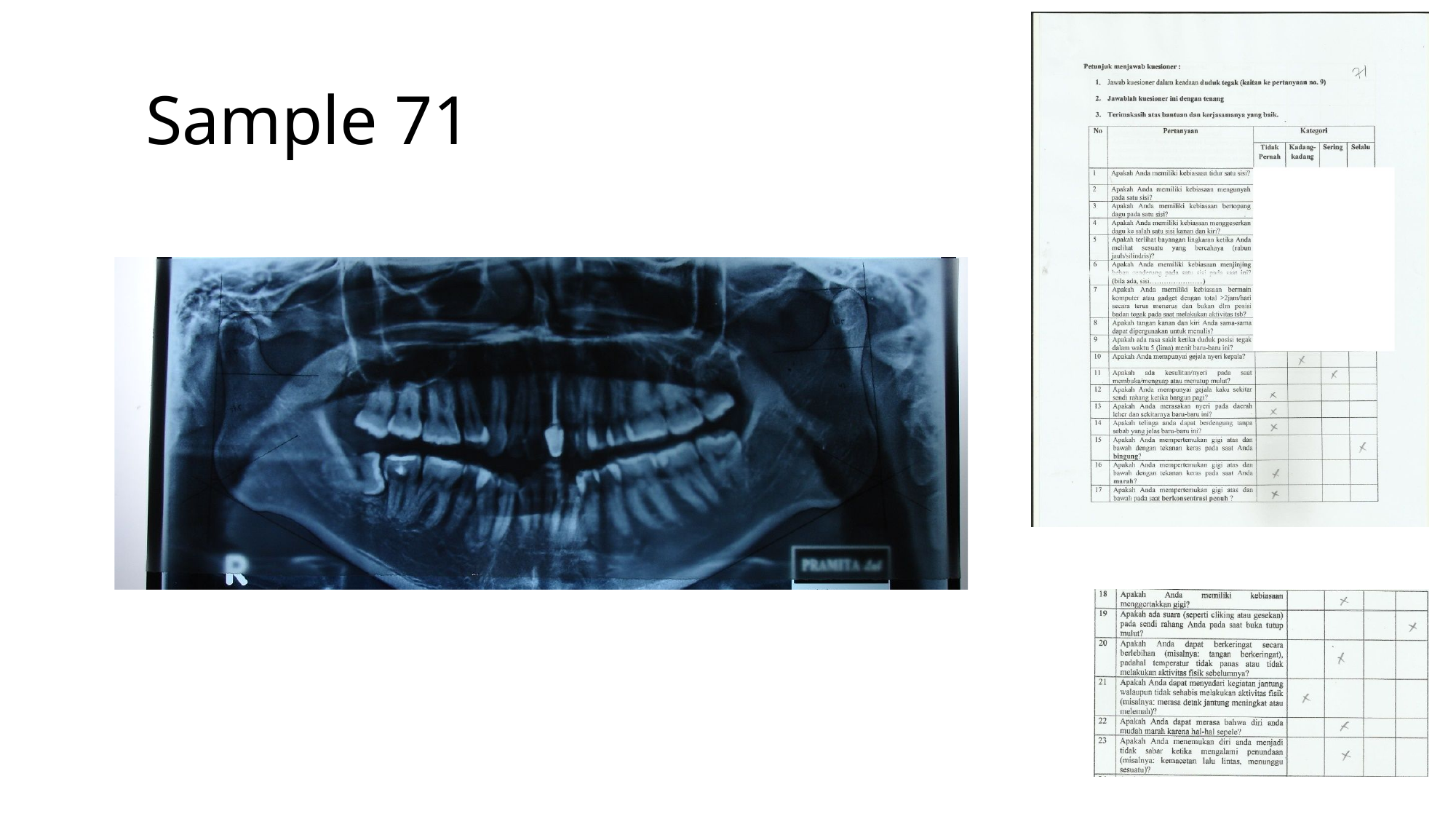

# Sample 71

## Slide 56
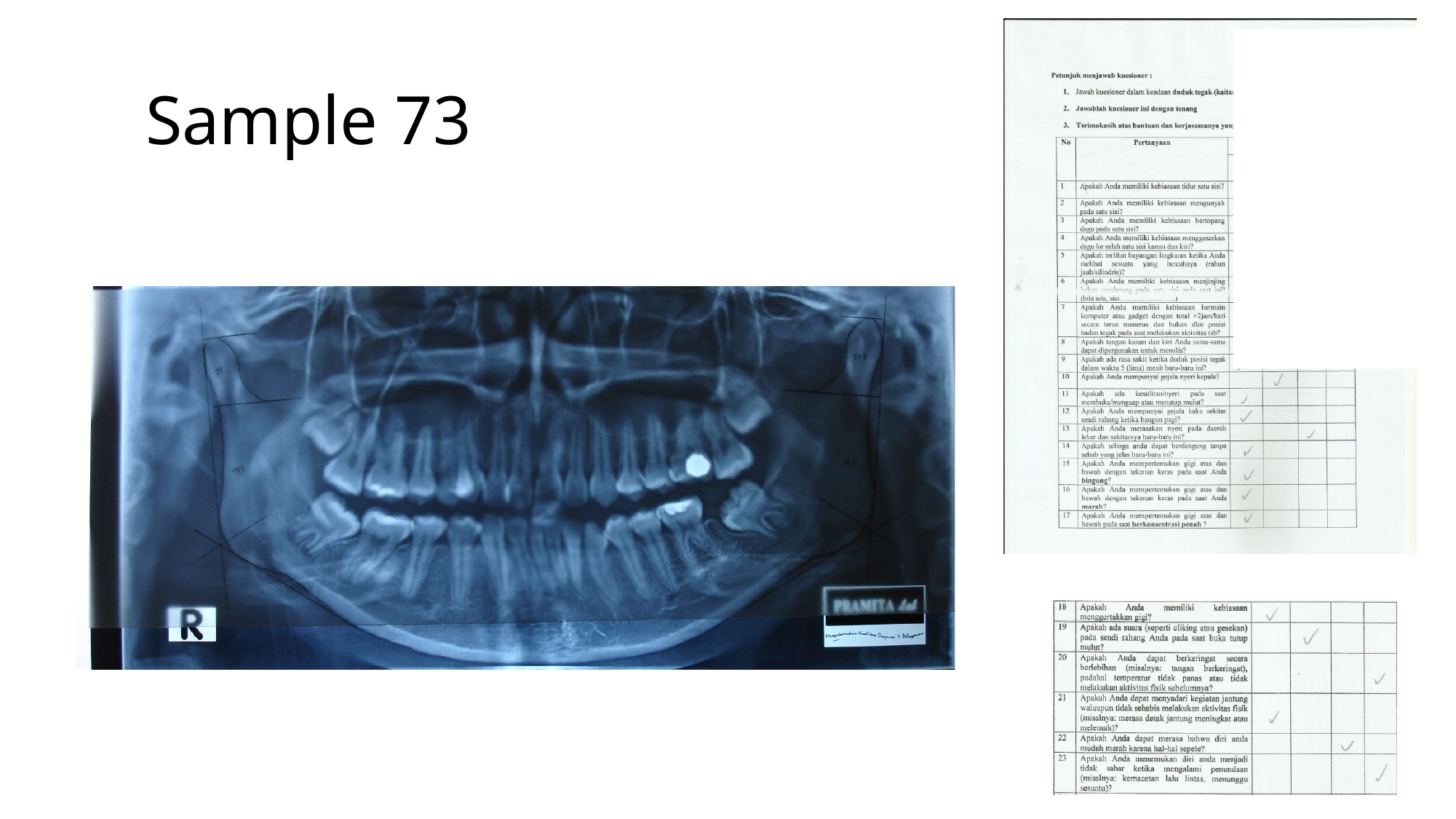

# Sample 73

## Slide 57
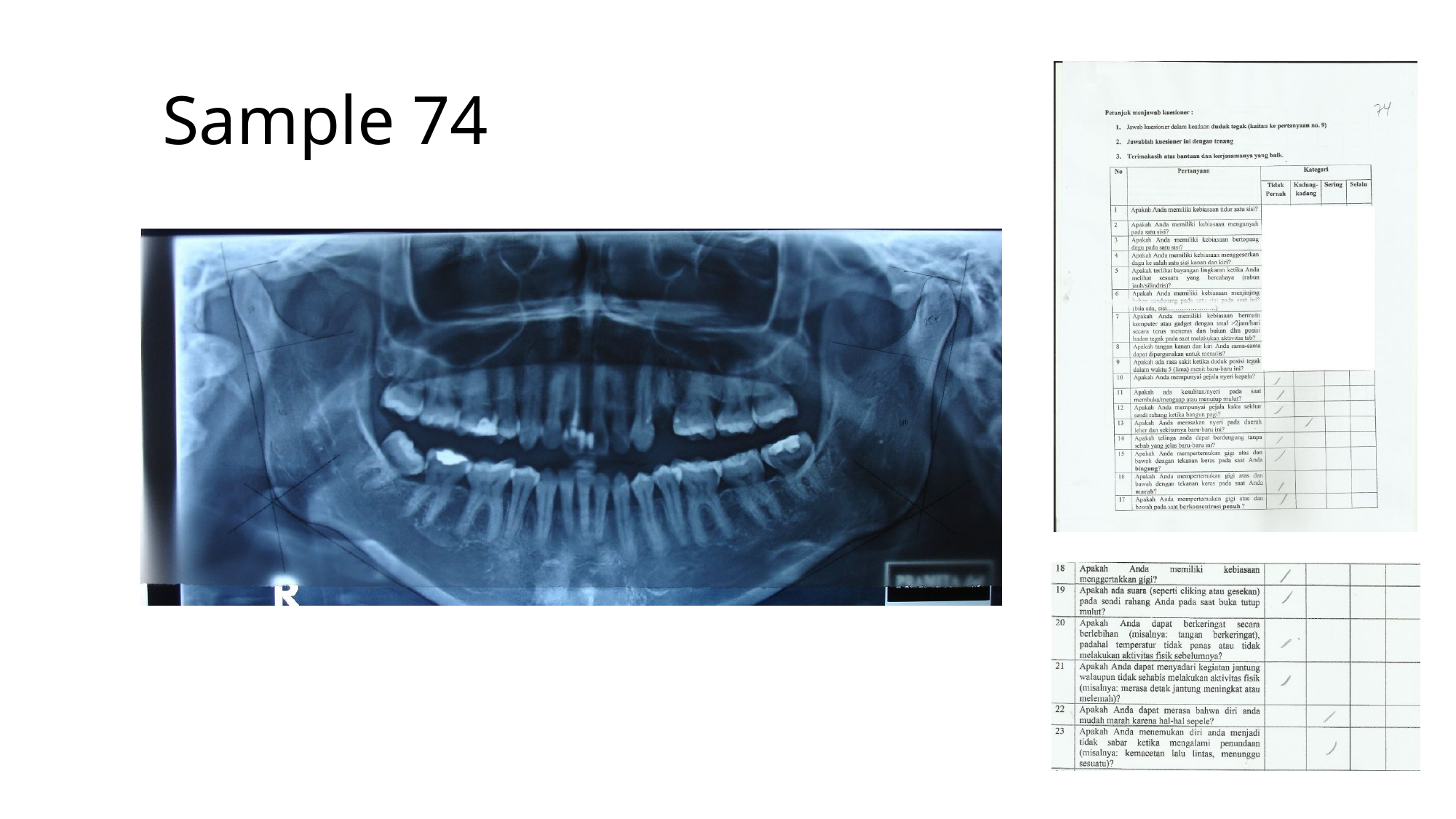

# Sample 74

## Slide 58
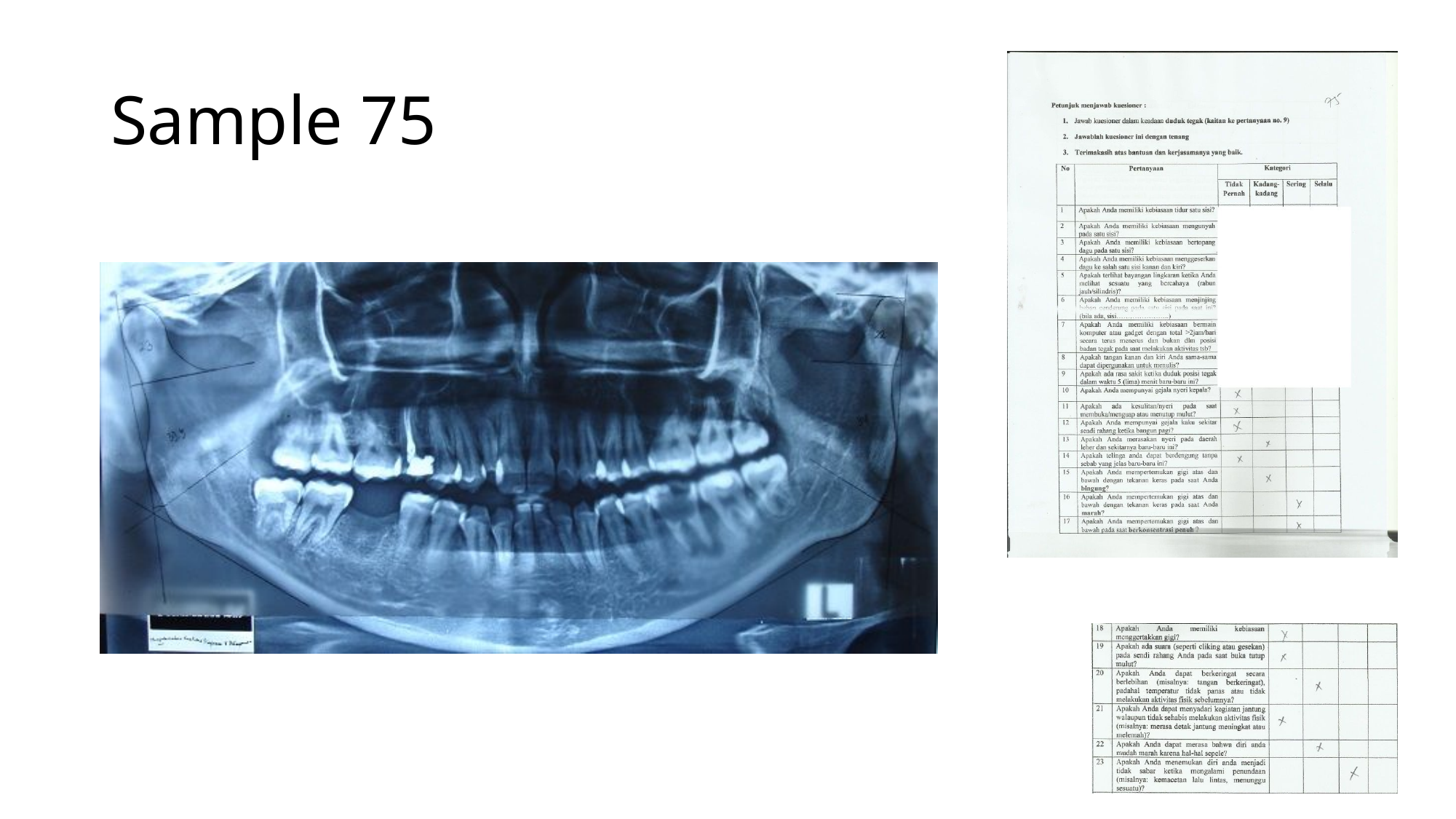

# Sample 75

## Slide 59
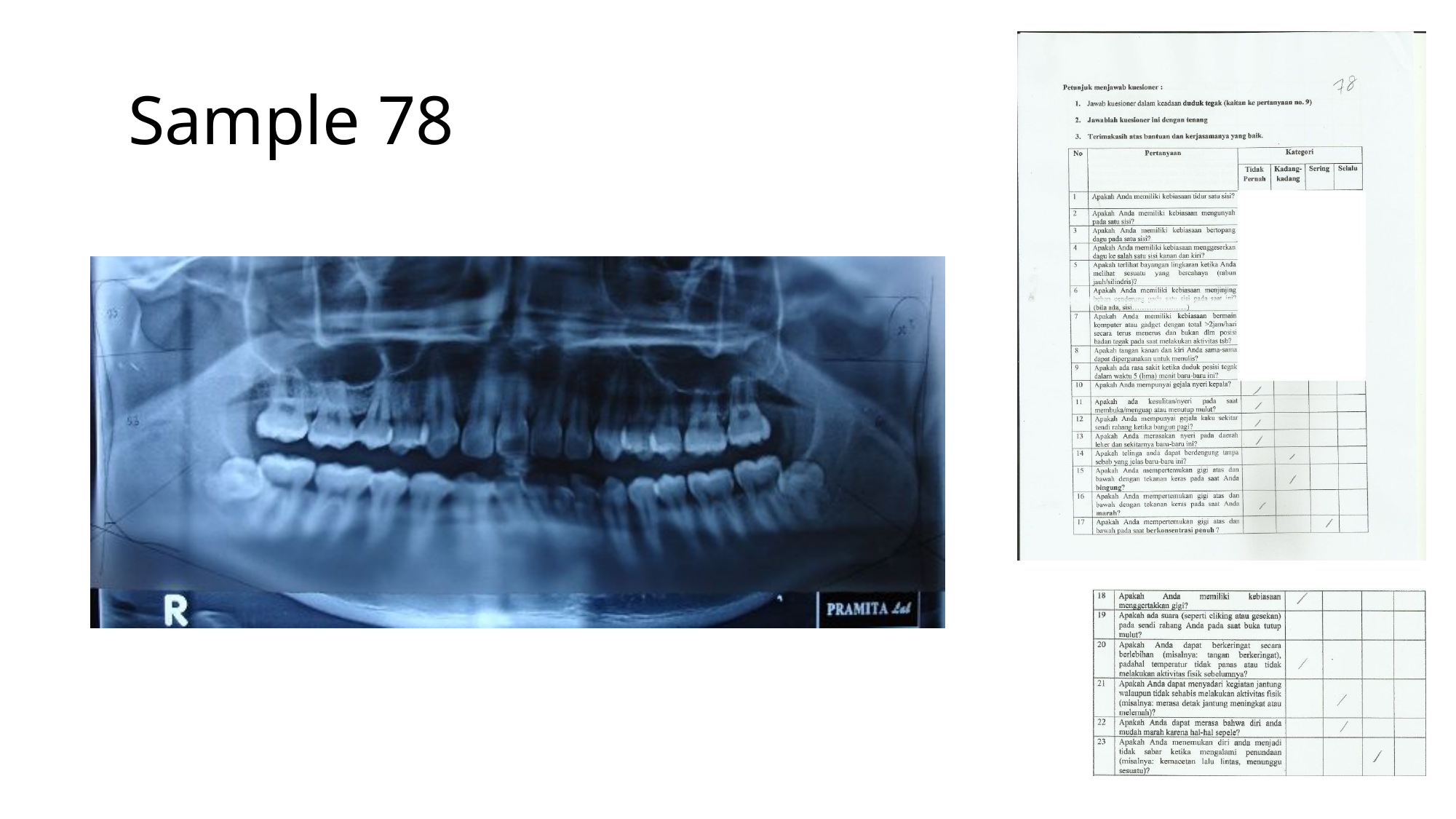

# Sample 78

## Slide 60
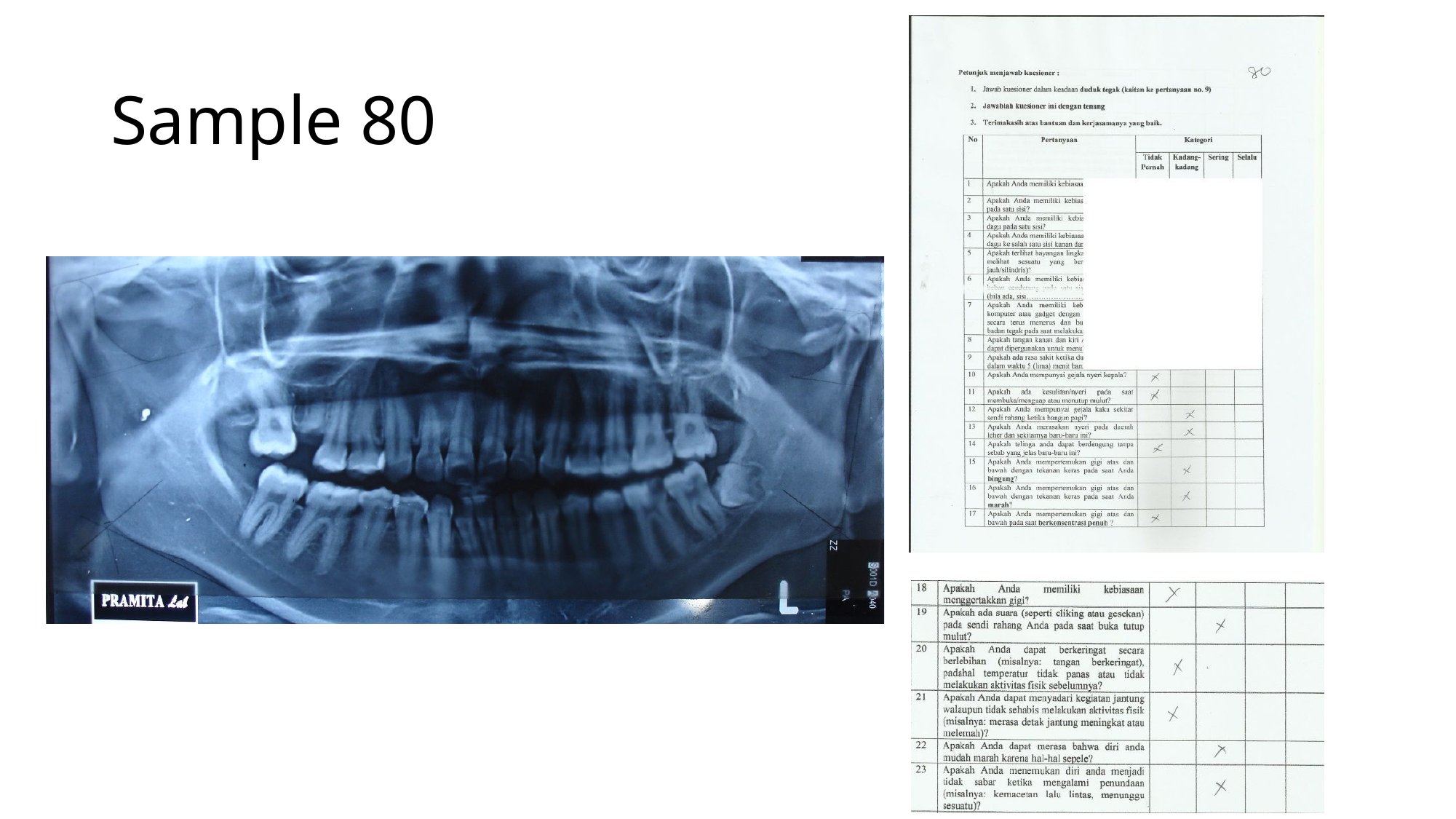

# Sample 80

## Slide 61
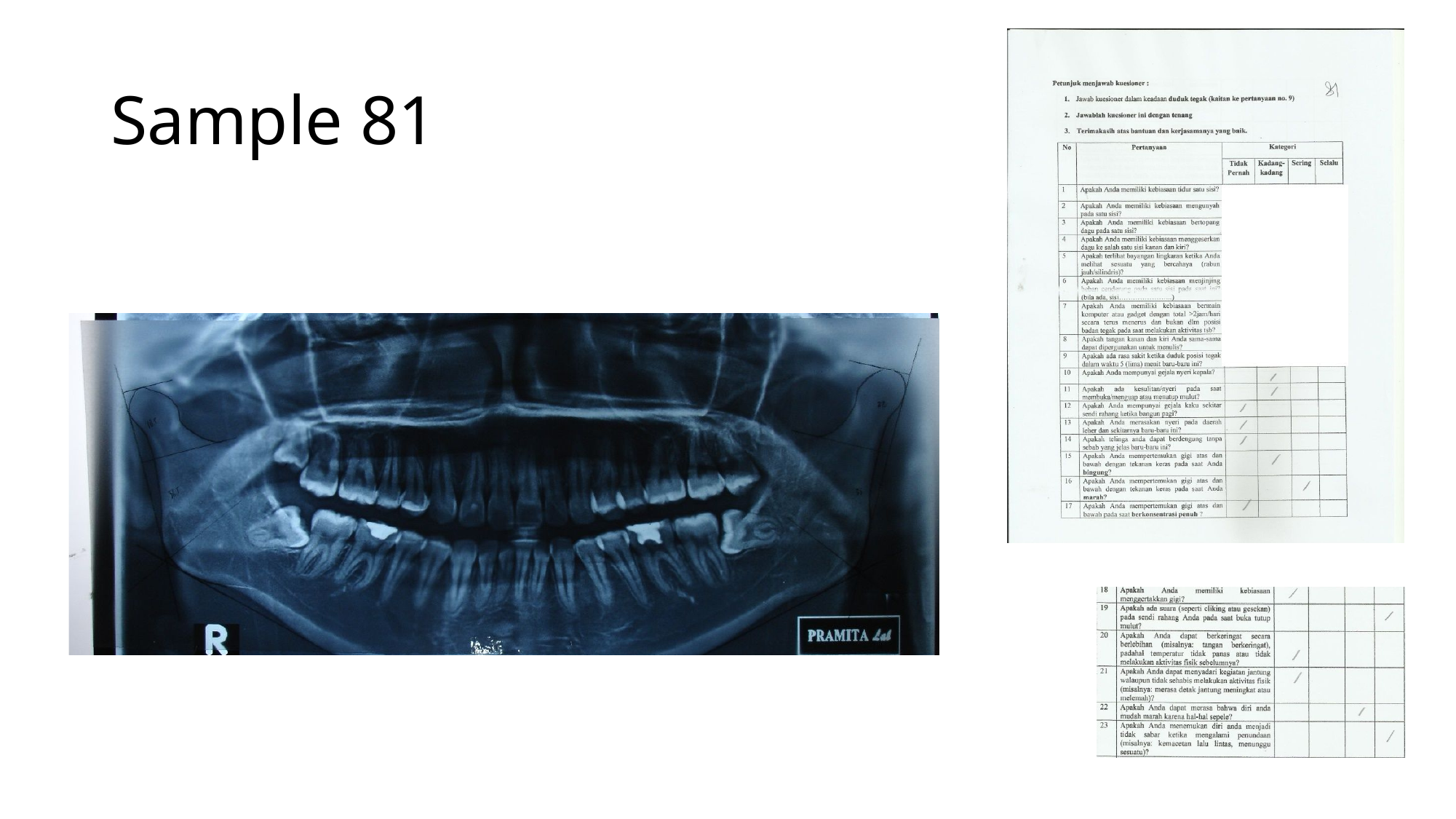

# Sample 81

## Slide 62
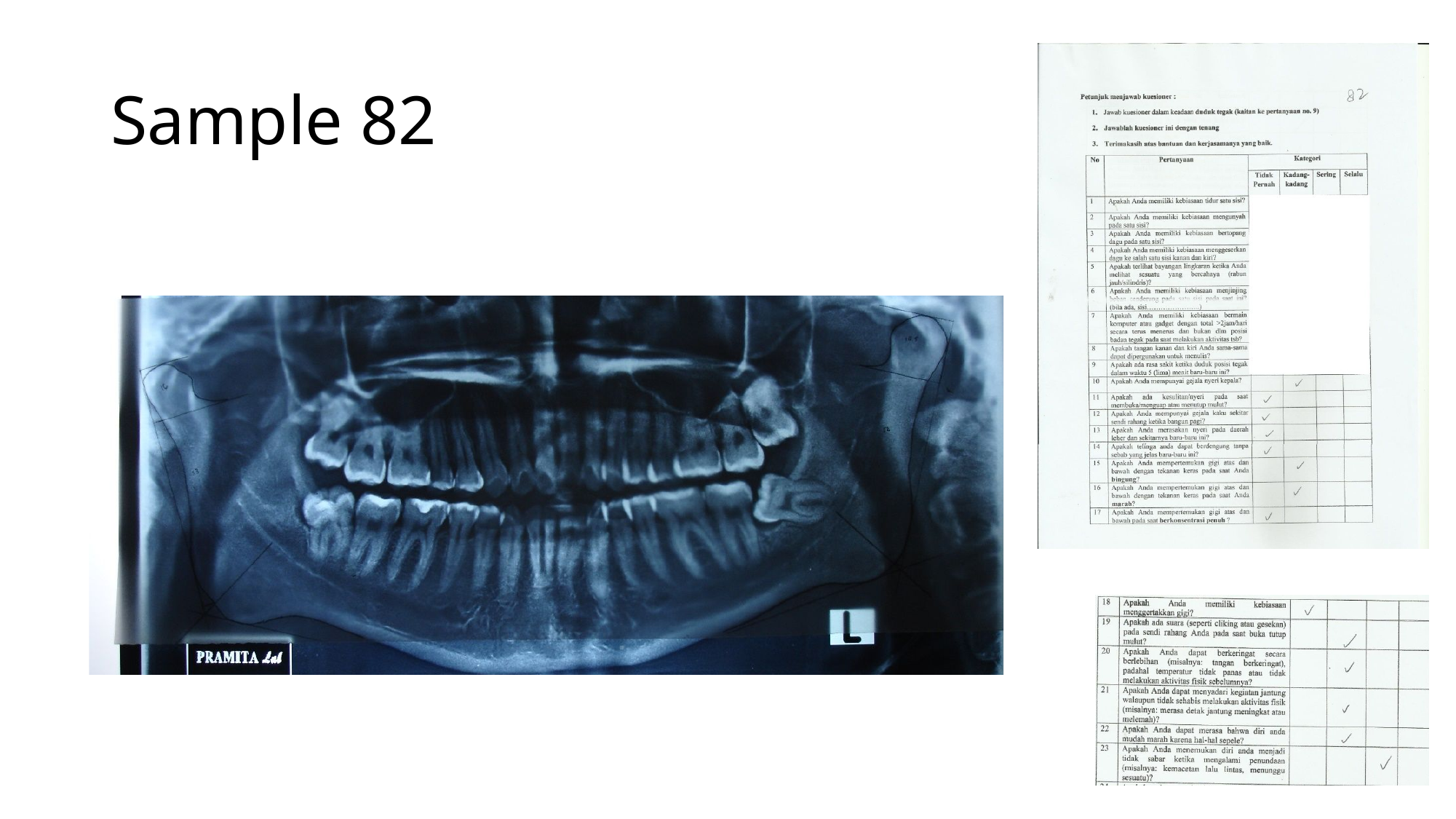

# Sample 82

## Slide 63
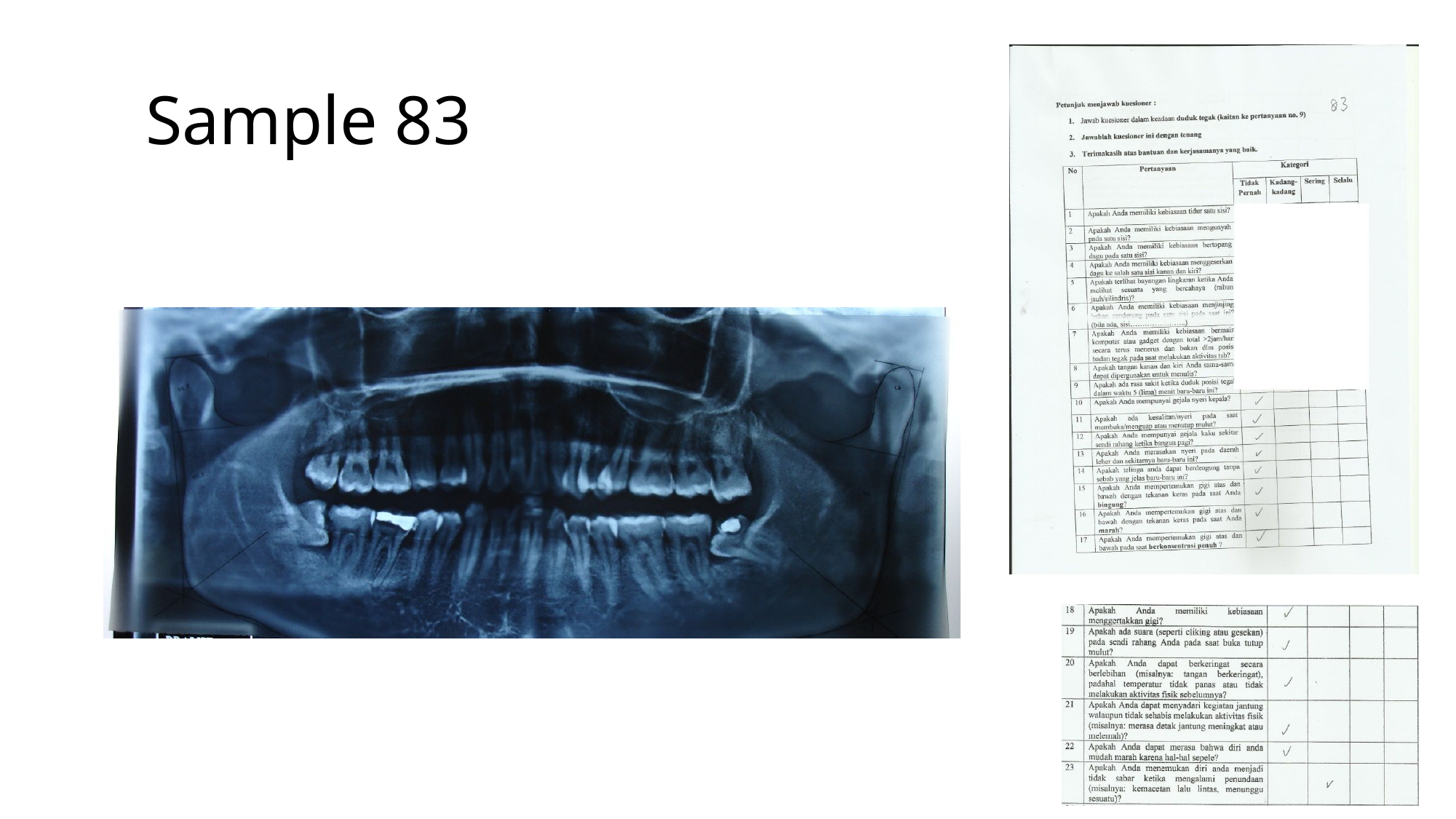

# Sample 83

## Slide 64
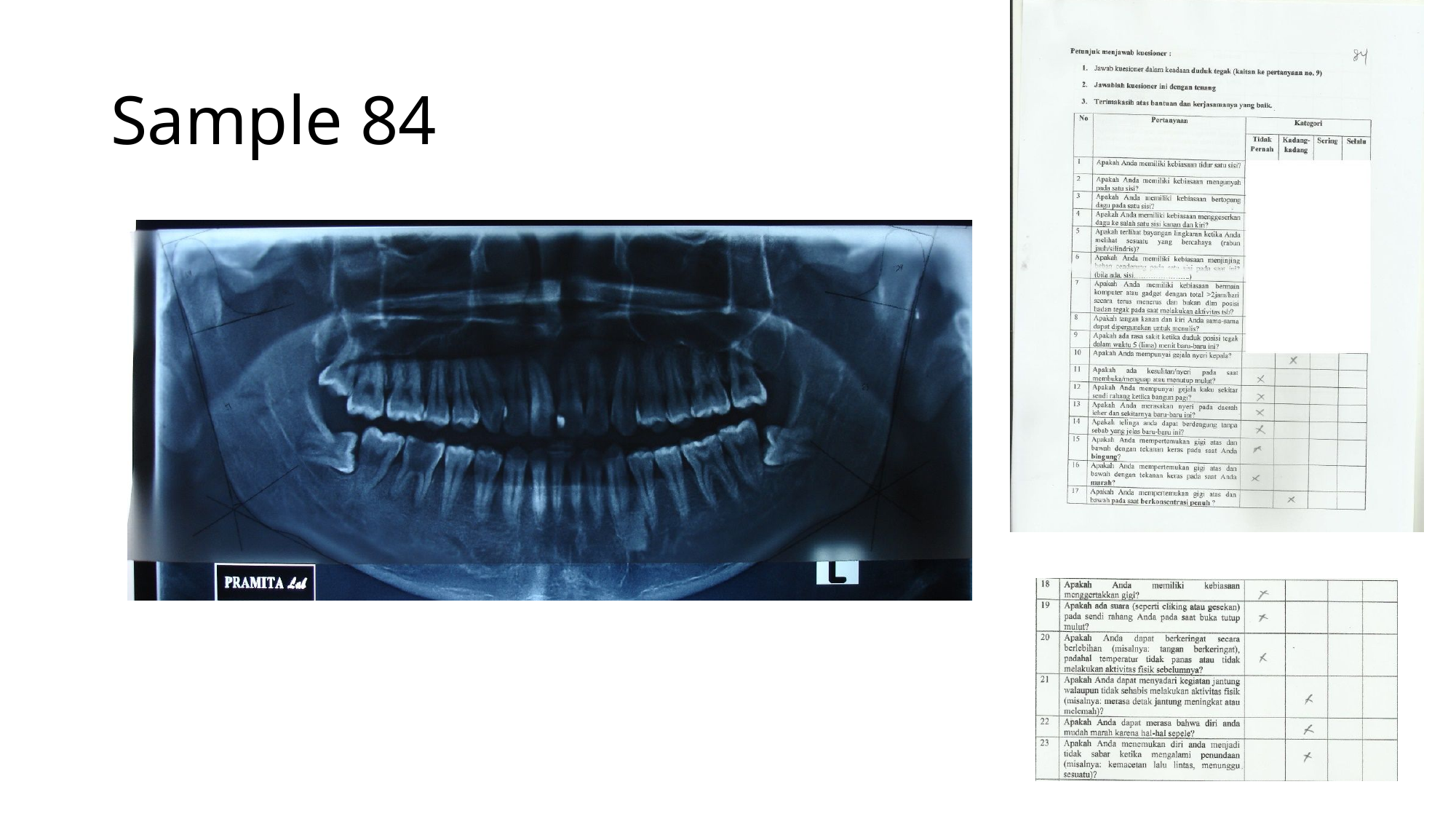

# Sample 84

## Slide 65
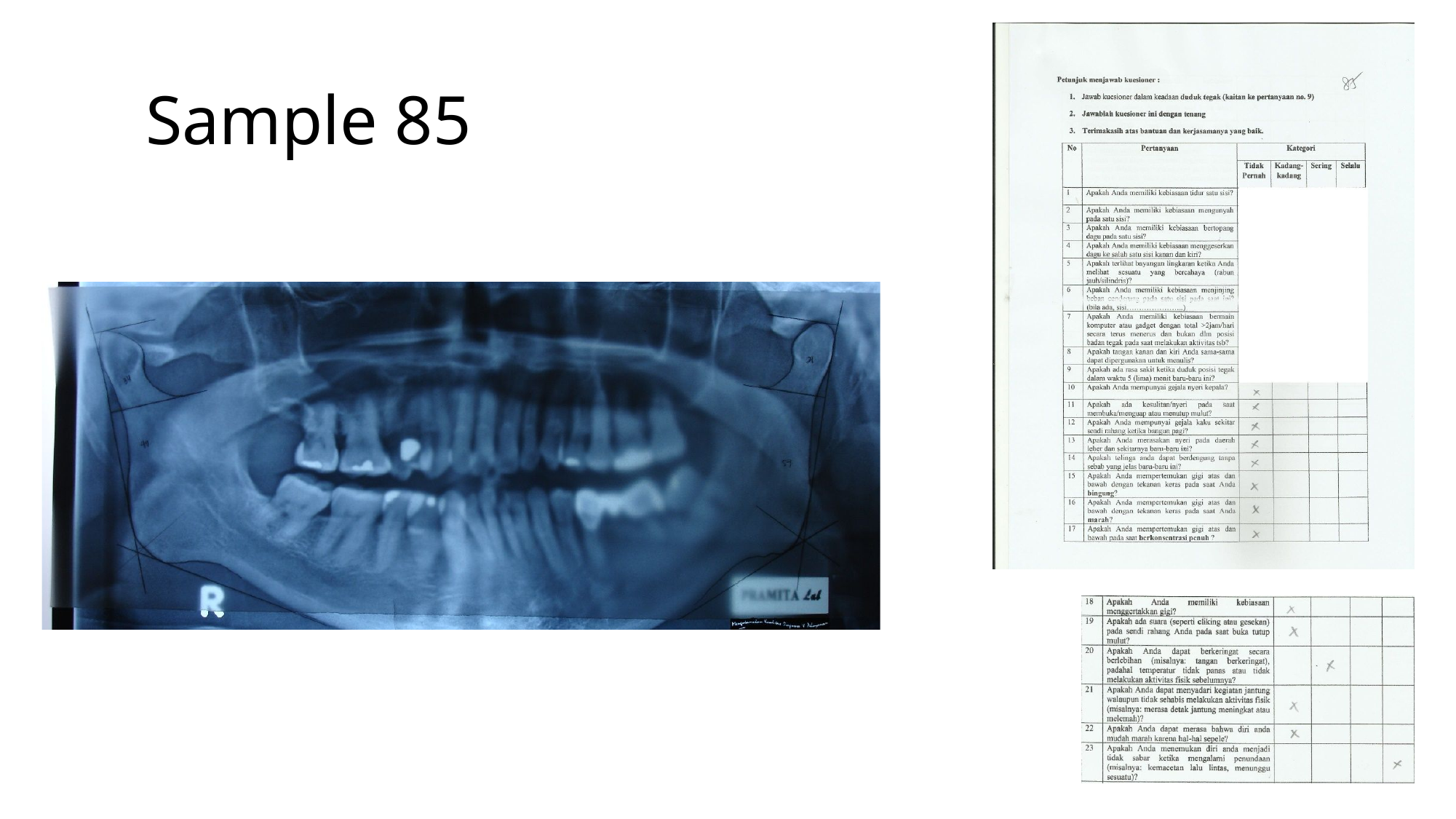

# Sample 85

## Slide 66
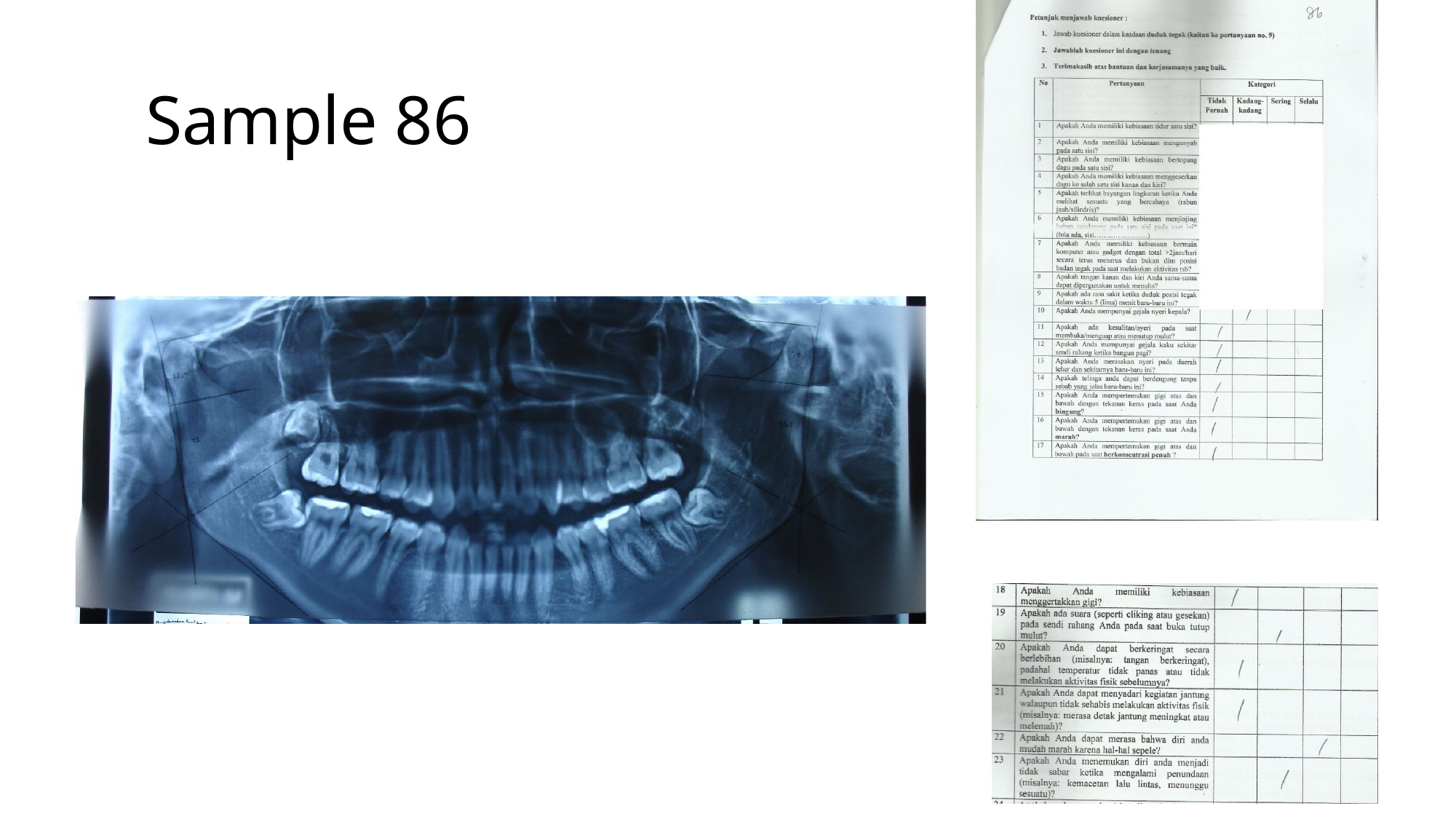

# Sample 86

## Slide 67
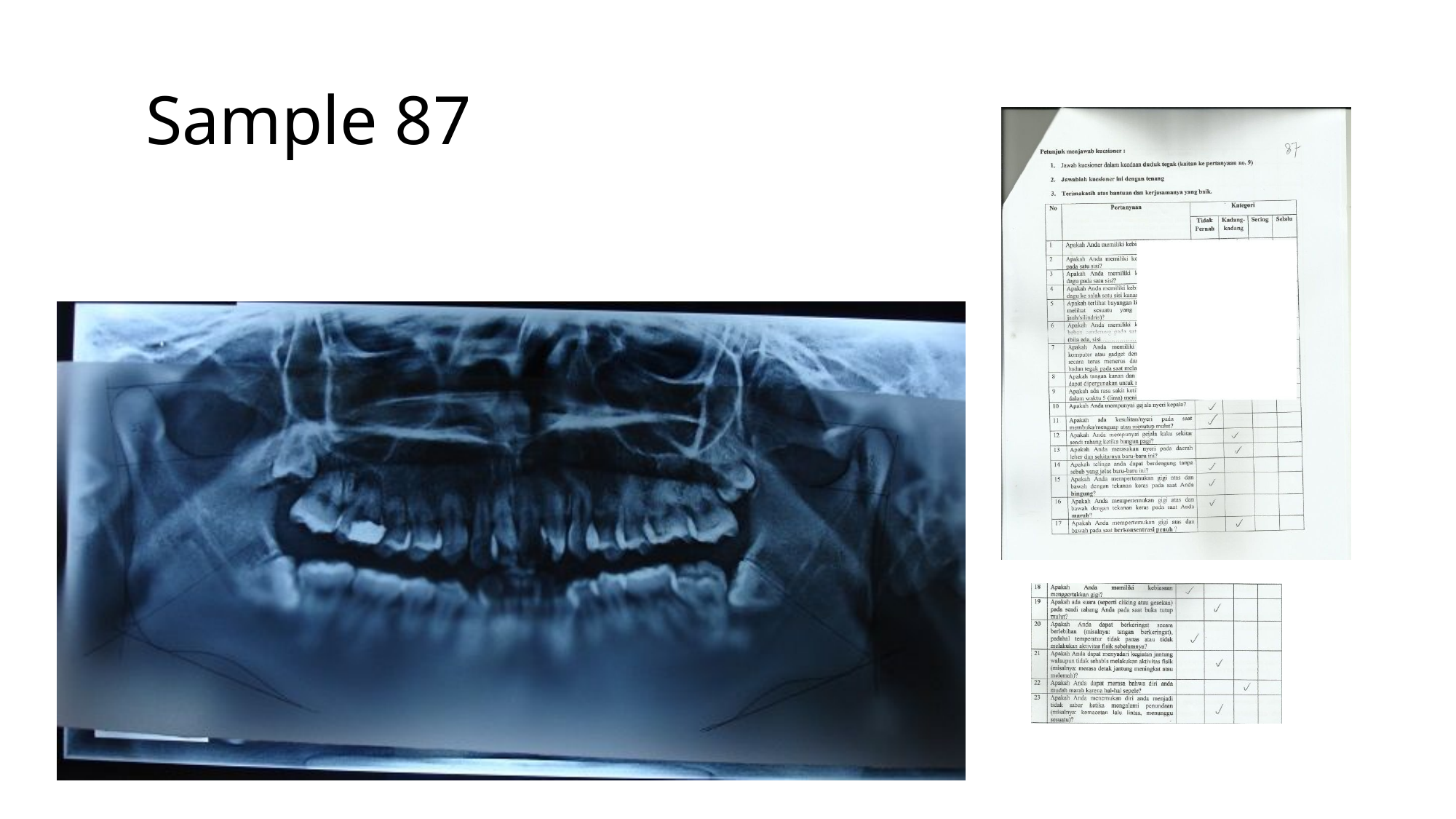

# Sample 87

## Slide 68
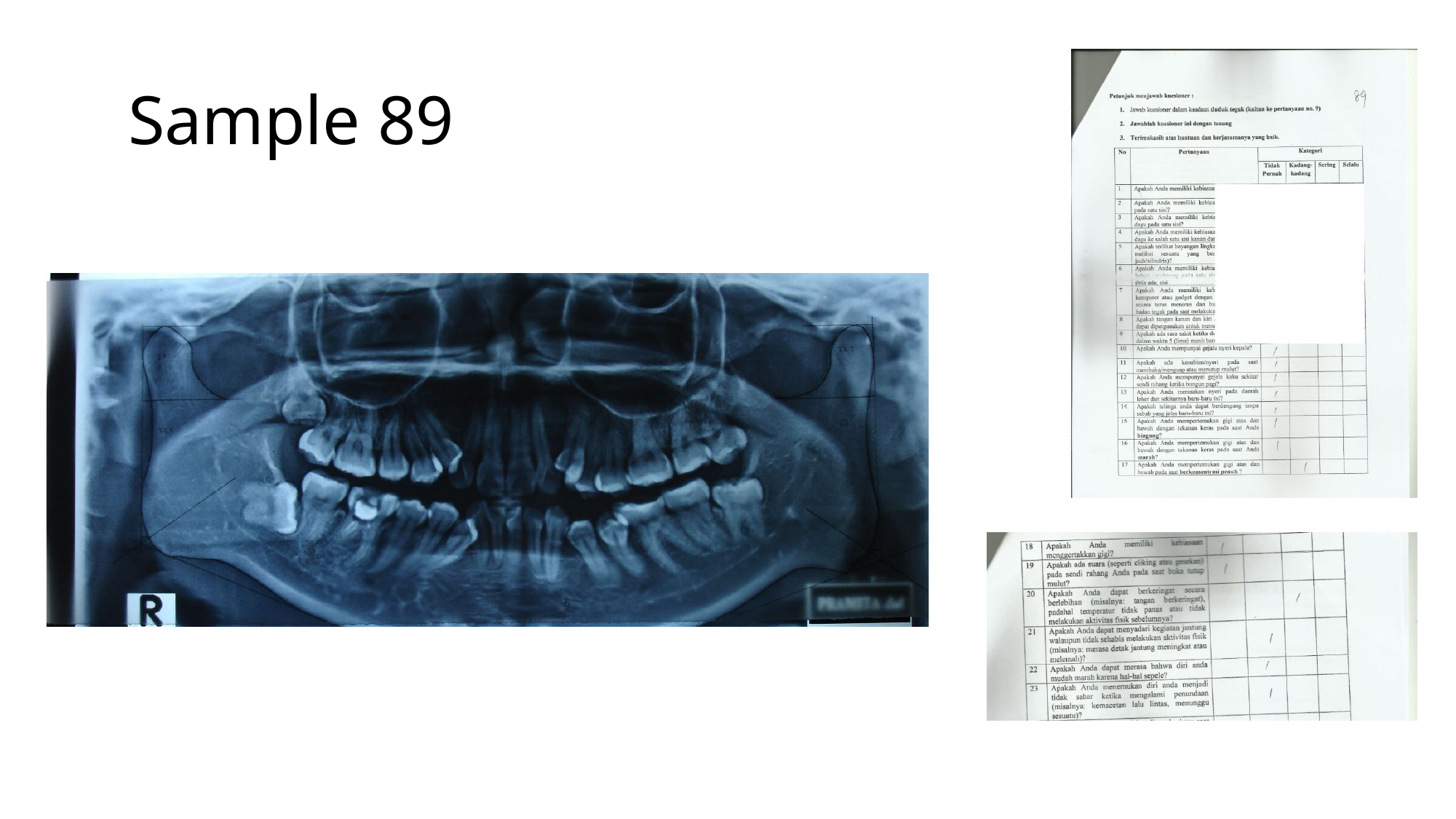

# Sample 89

## Slide 69
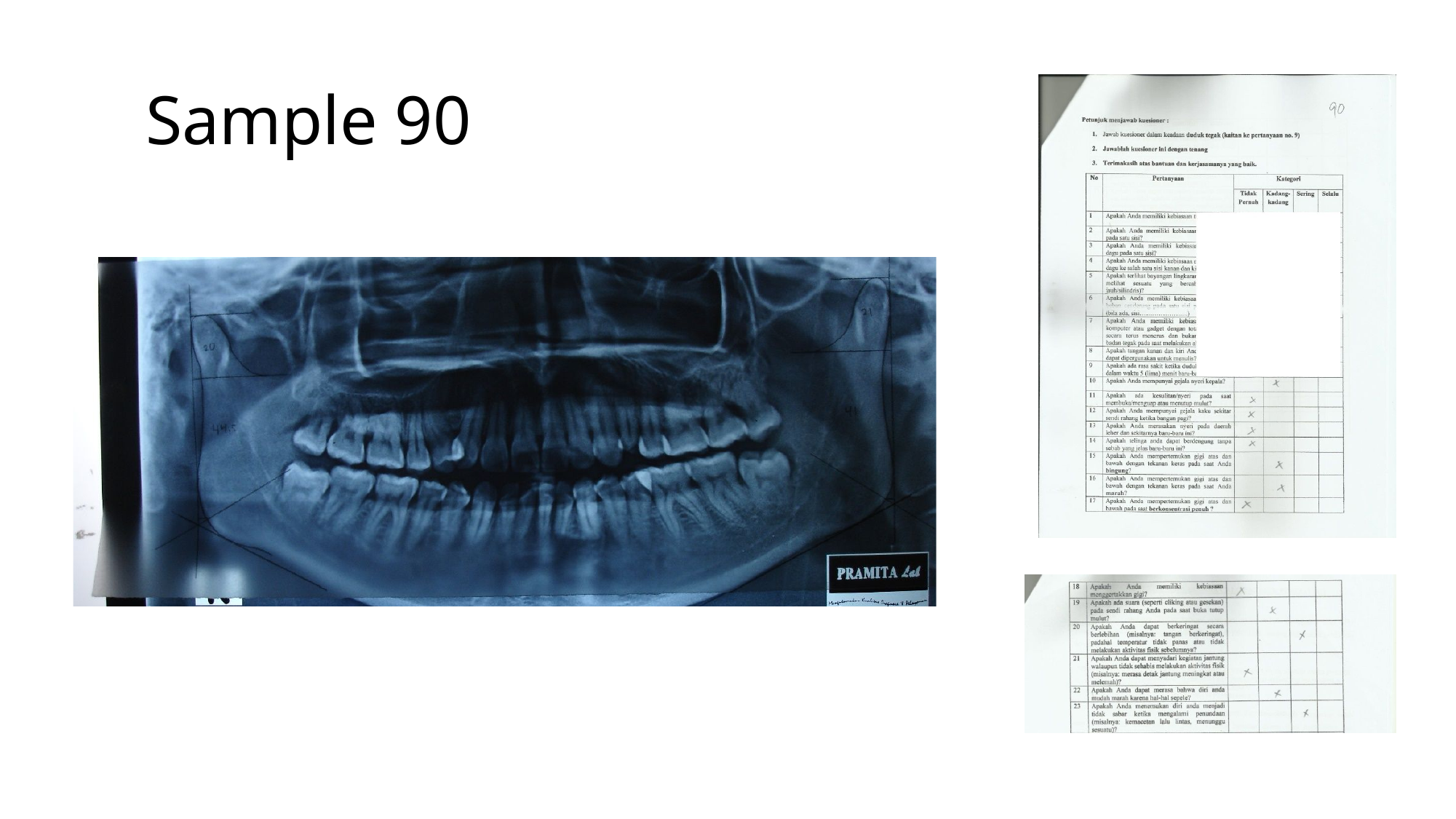

# Sample 90

## Slide 70
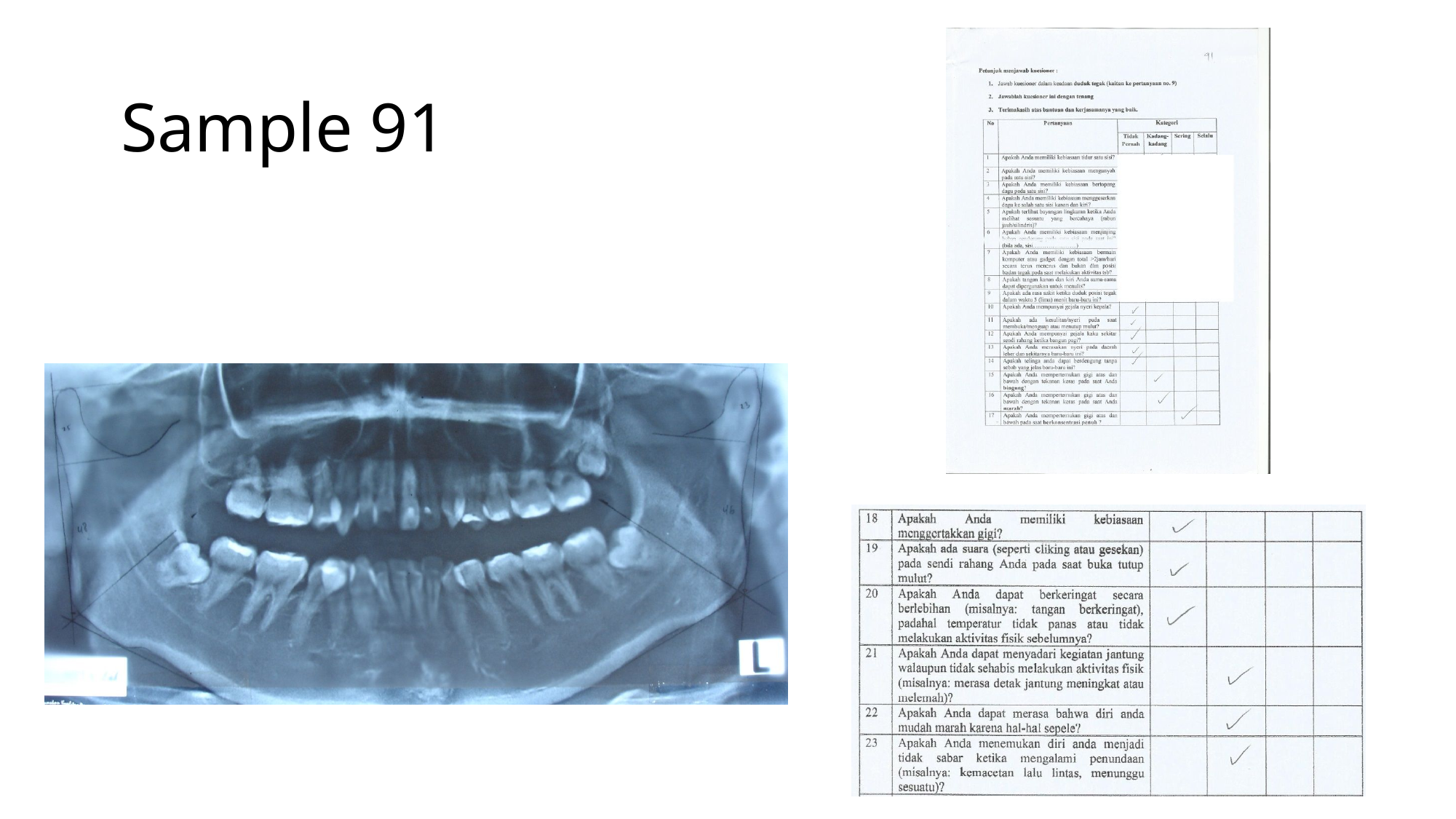

# Sample 91

## Slide 71
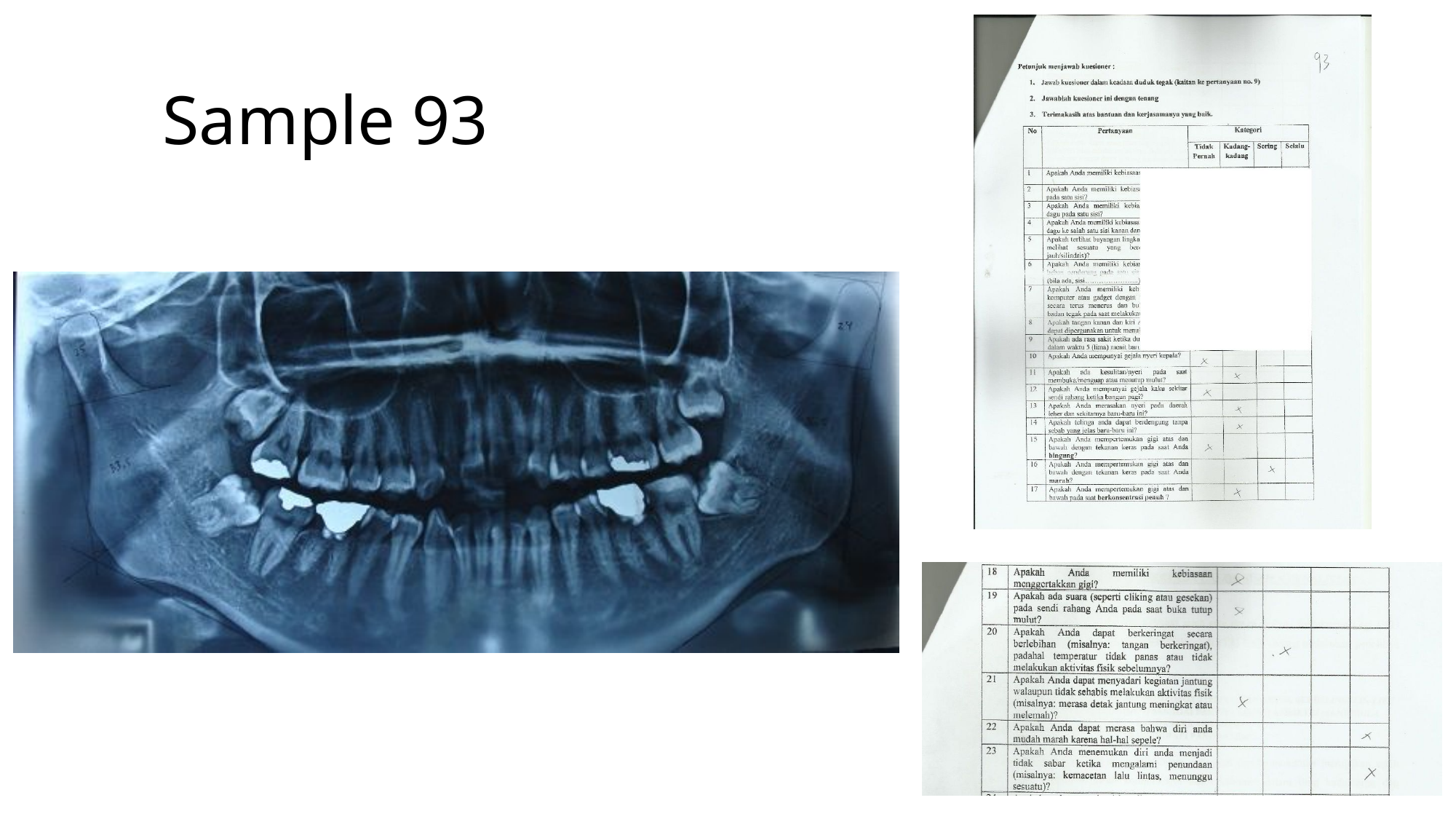

# Sample 93

## Slide 72
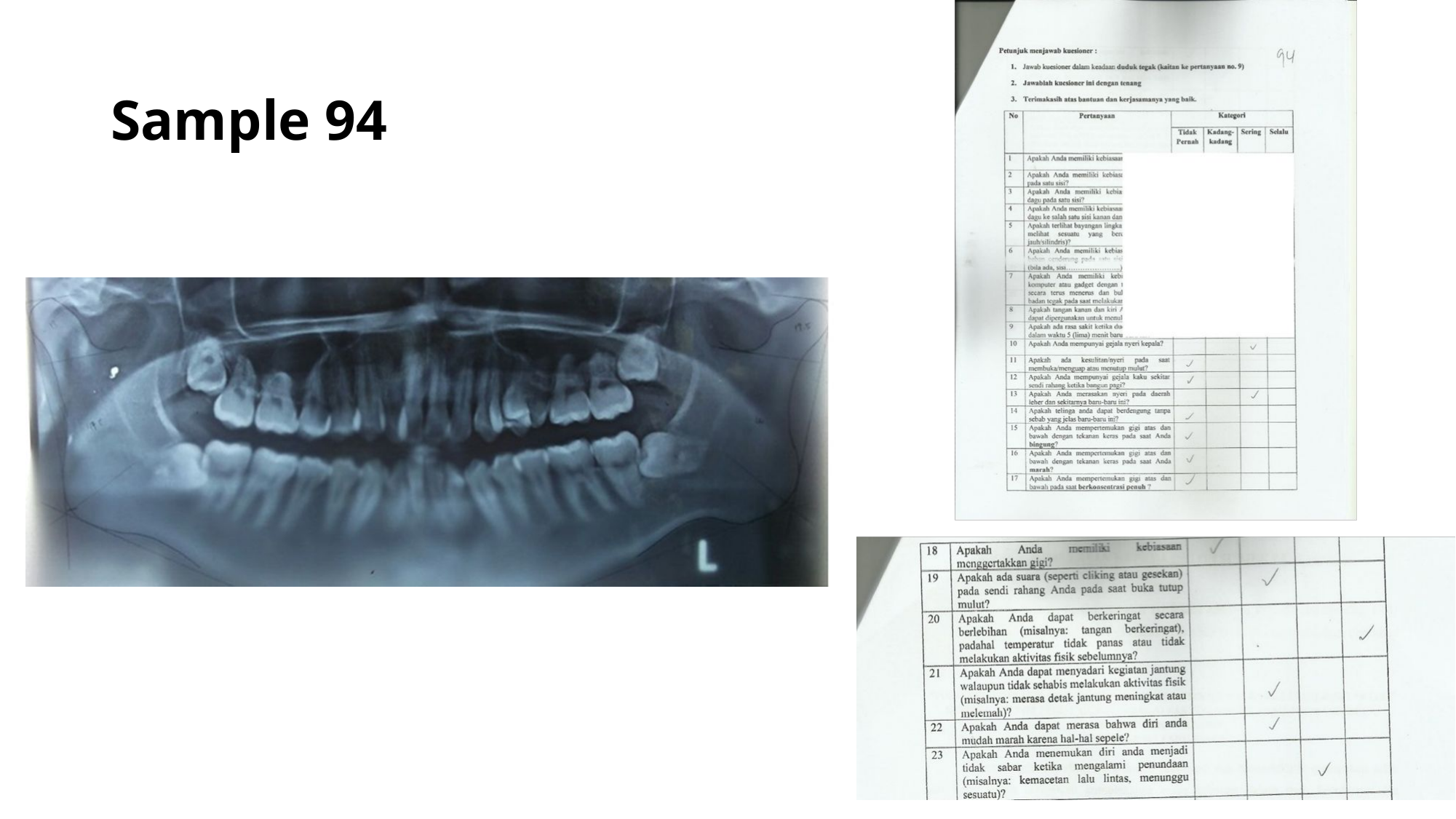

# Sample 94

## Slide 73
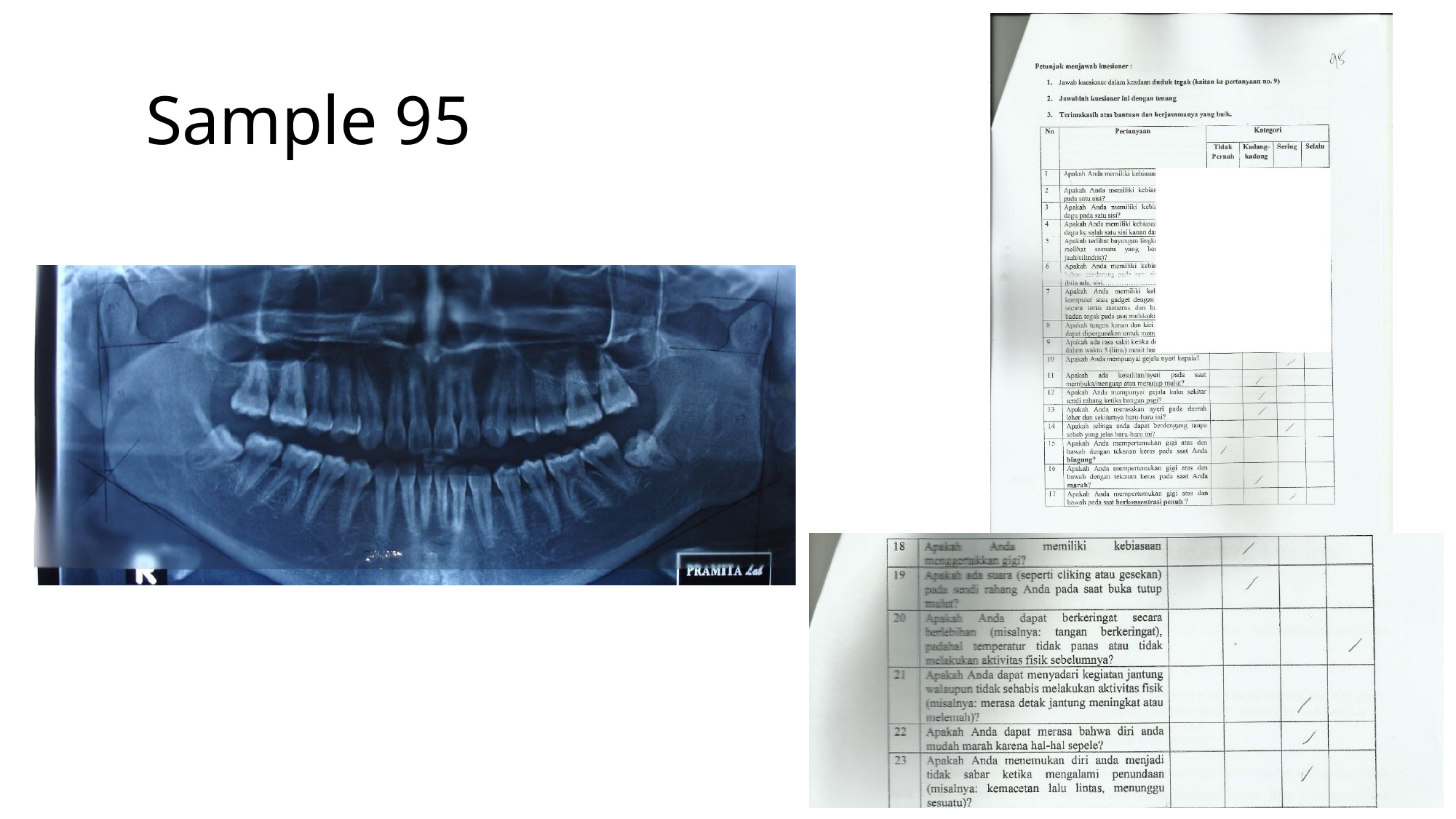

# Sample 95

## Slide 74
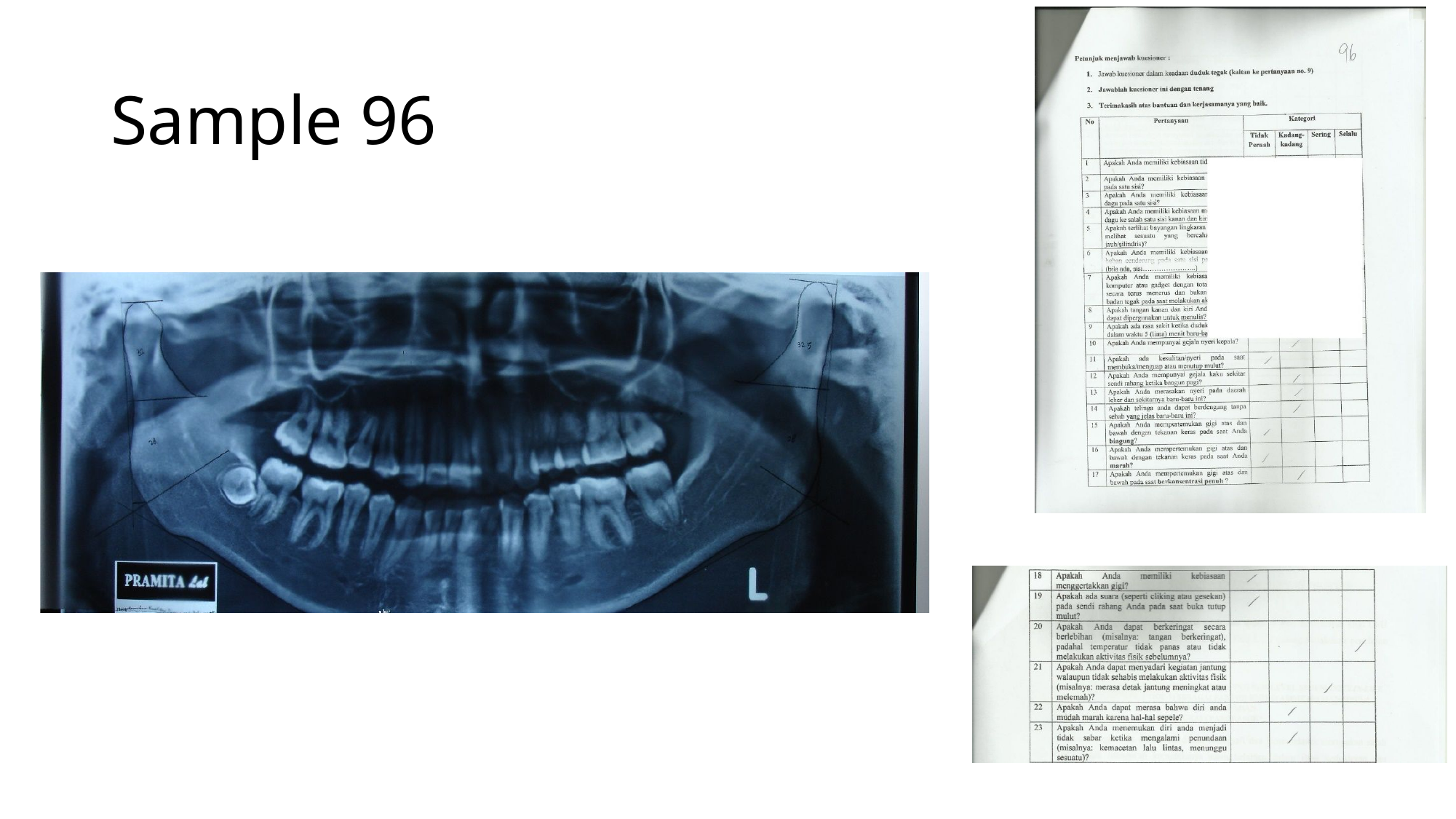

# Sample 96

## Slide 75
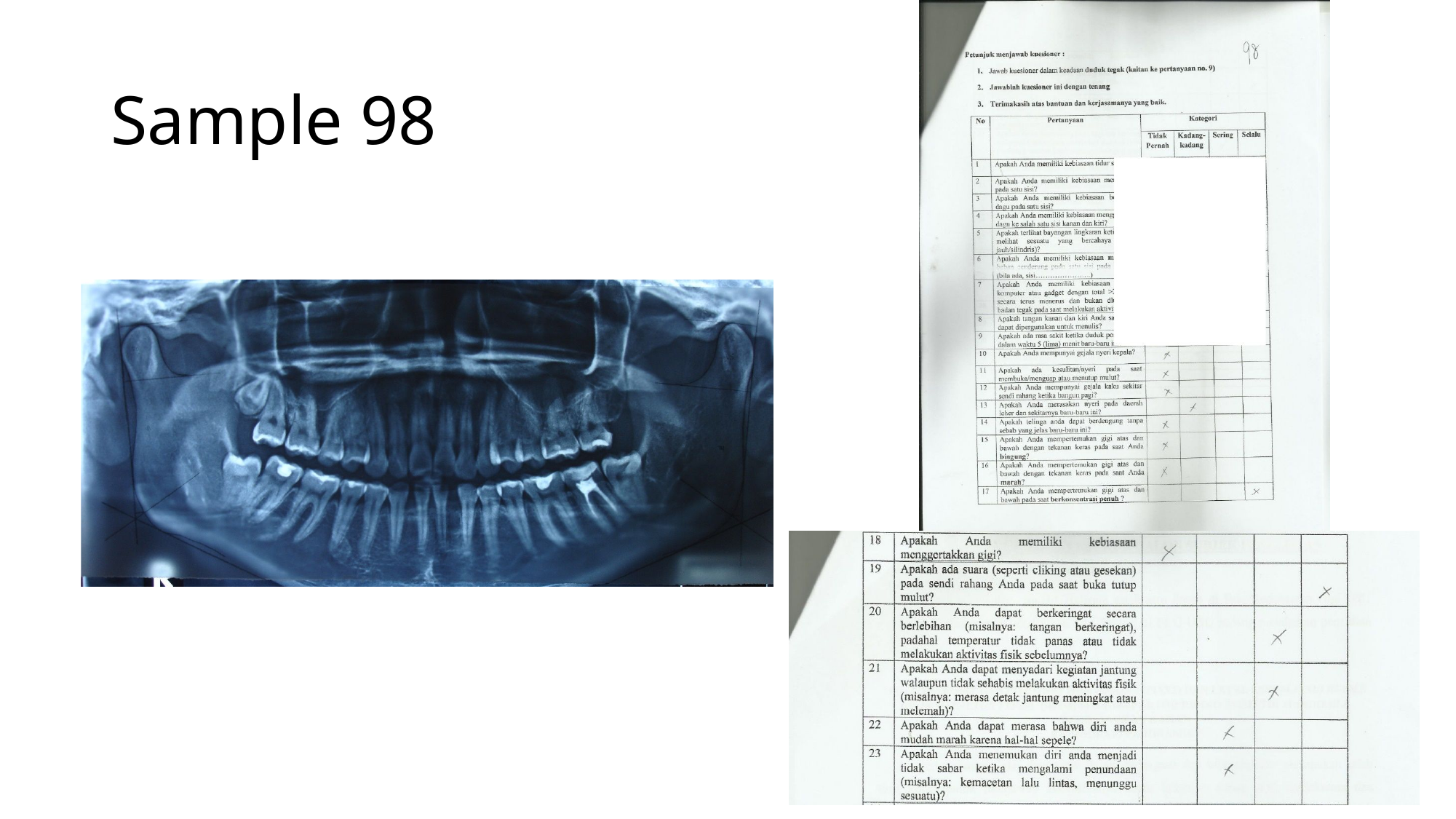

# Sample 98

## Slide 76
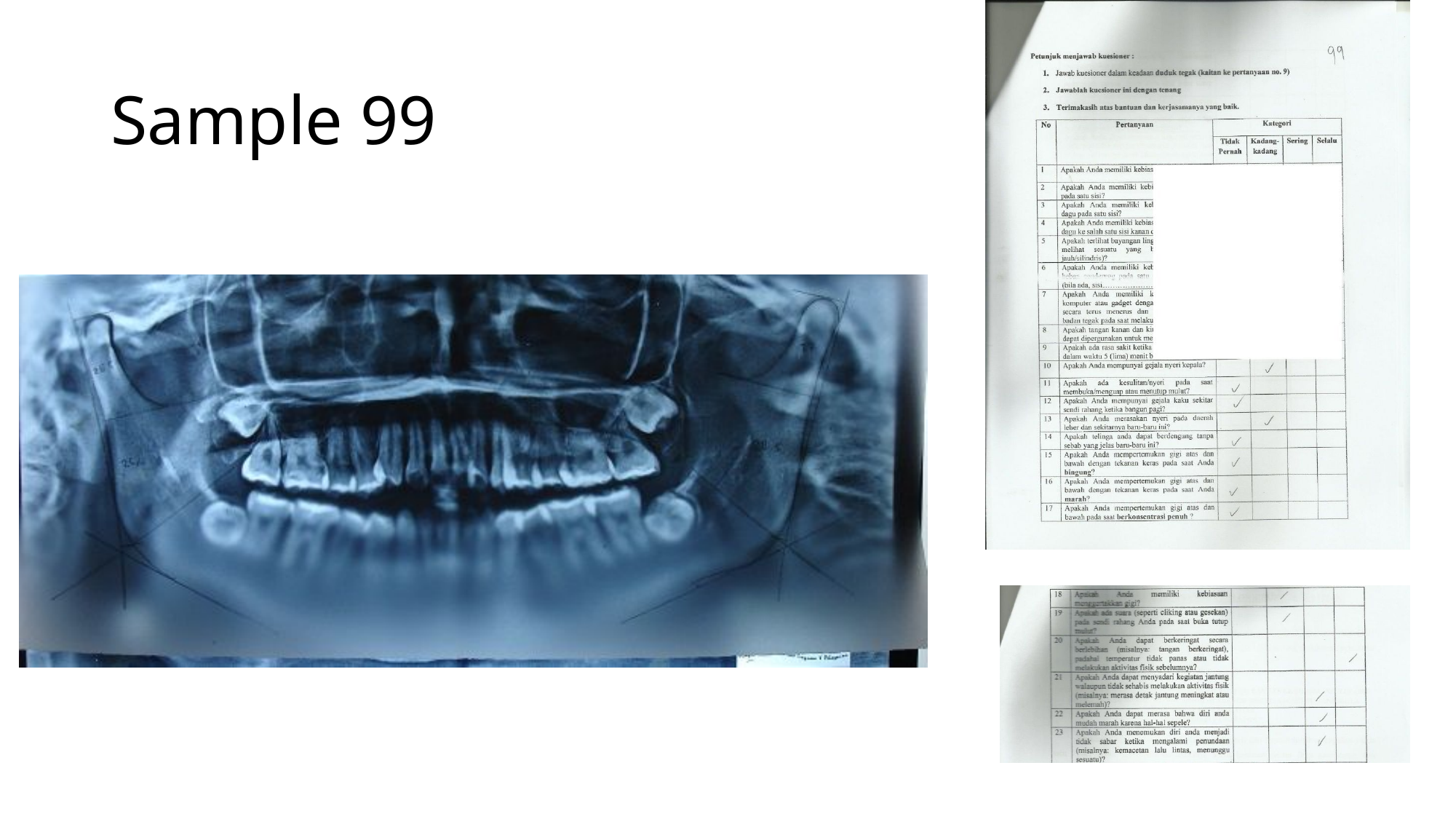

# Sample 99

## Slide 77
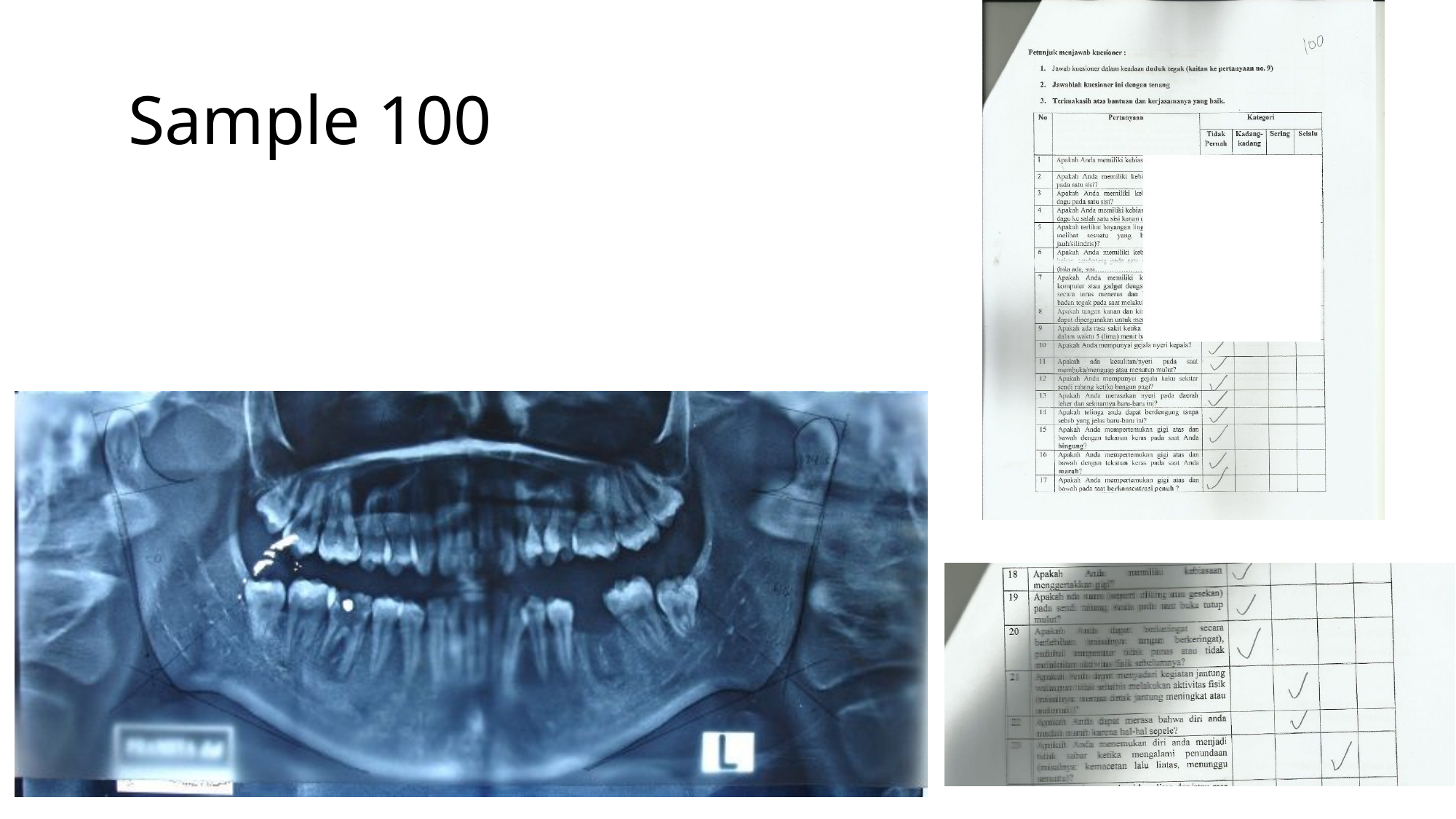

# Sample 100

## Slide 78
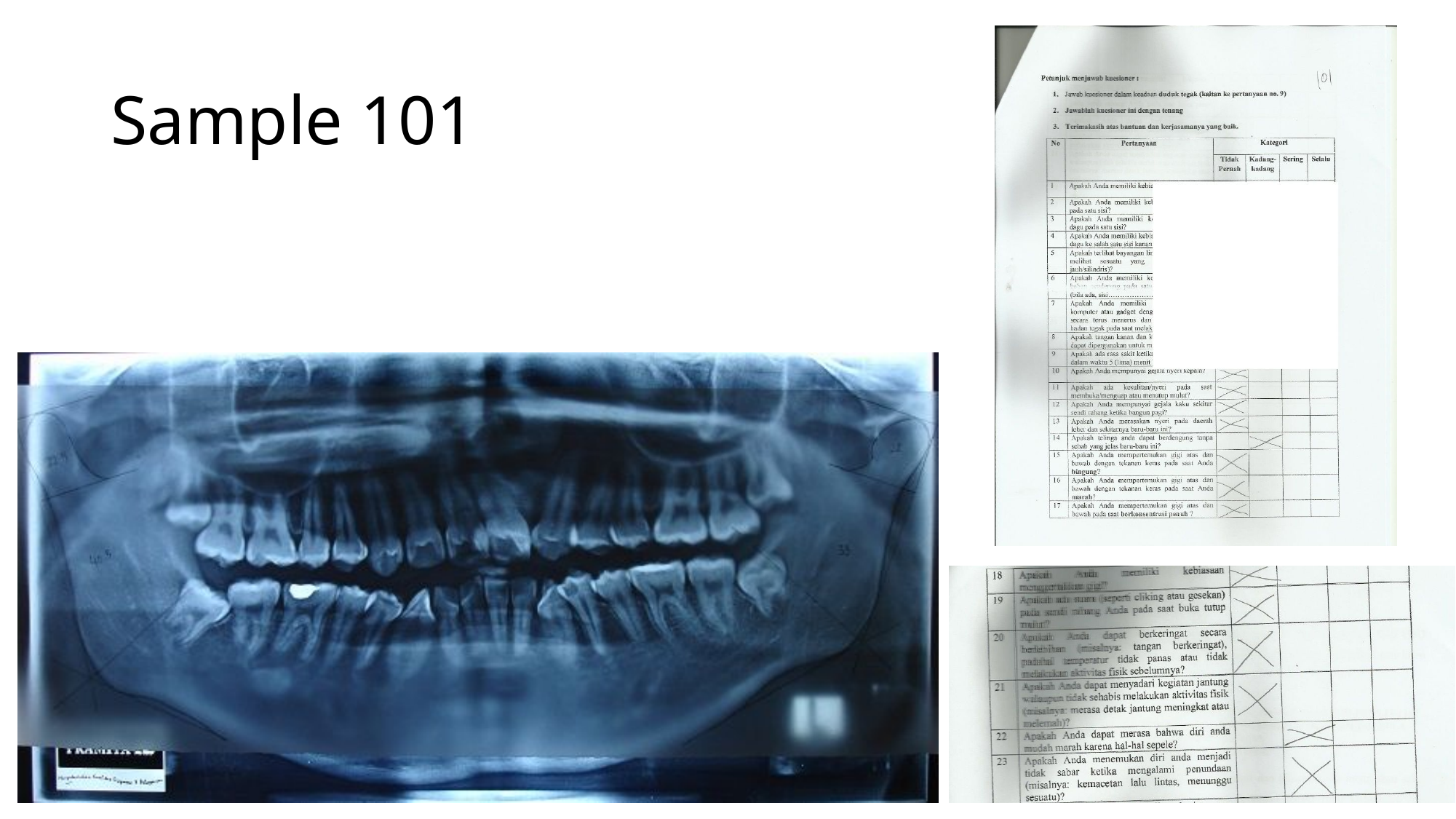

# Sample 101

## Slide 79
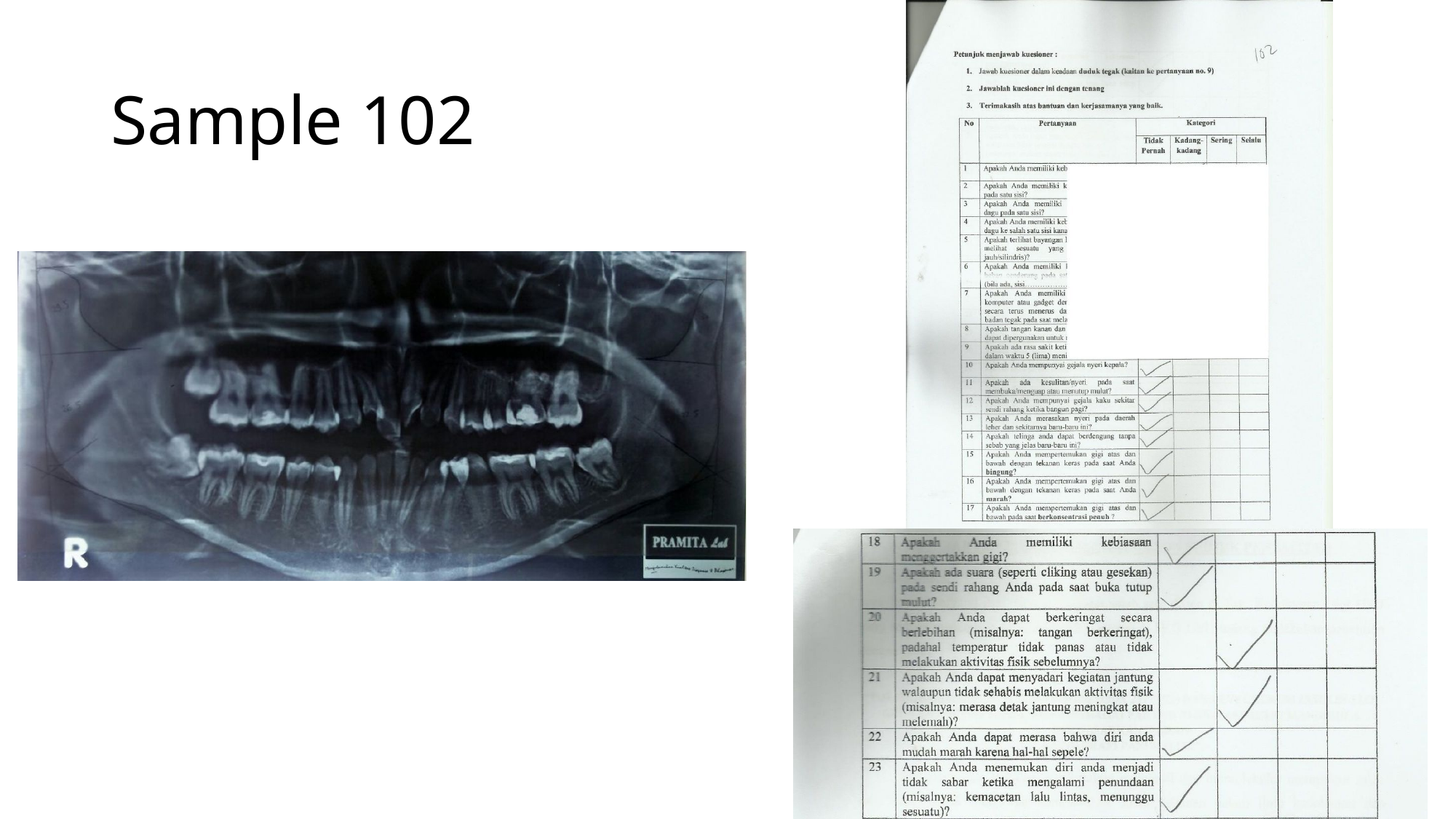

# Sample 102

## Slide 80
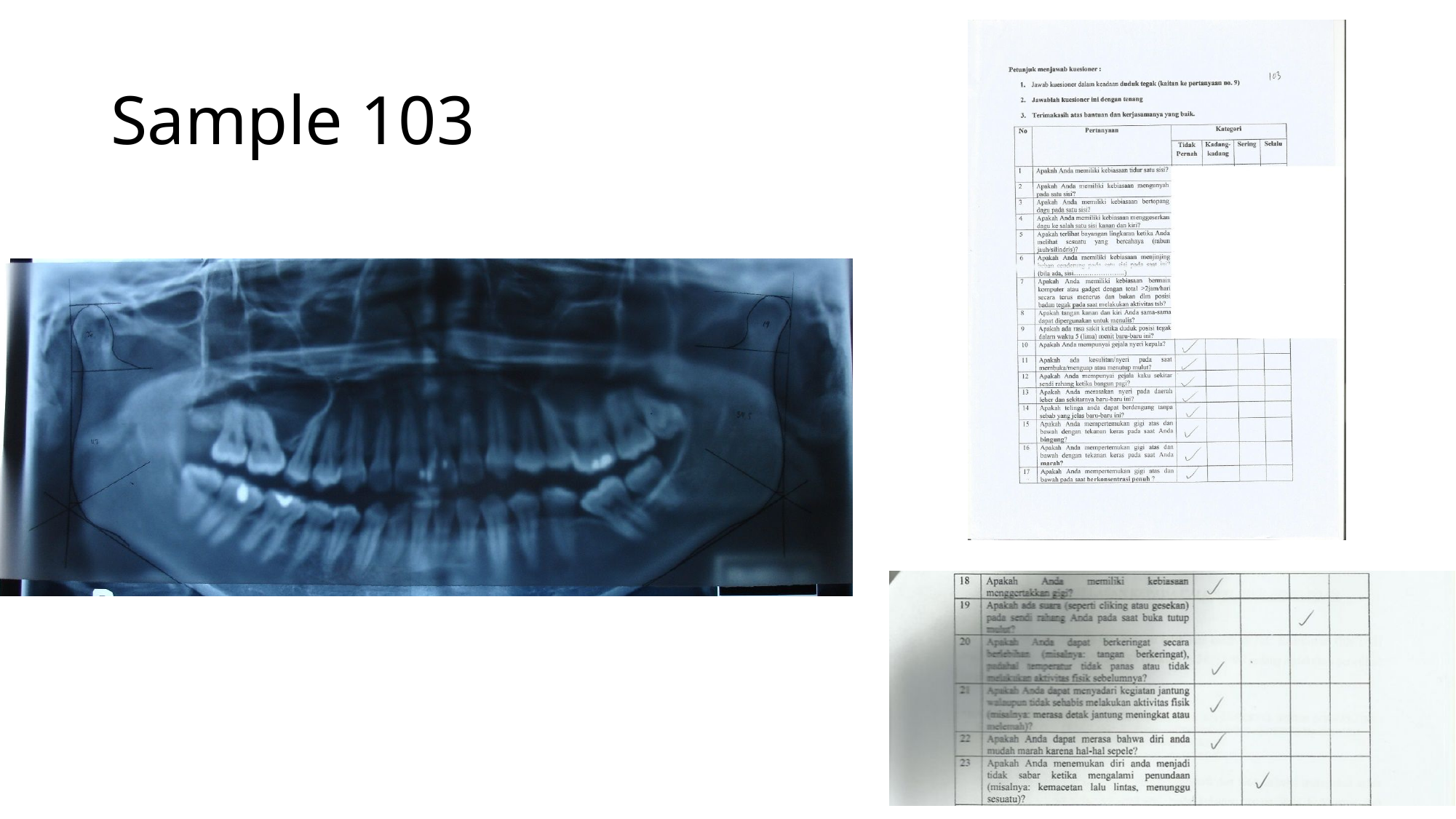

# Sample 103

## Slide 81
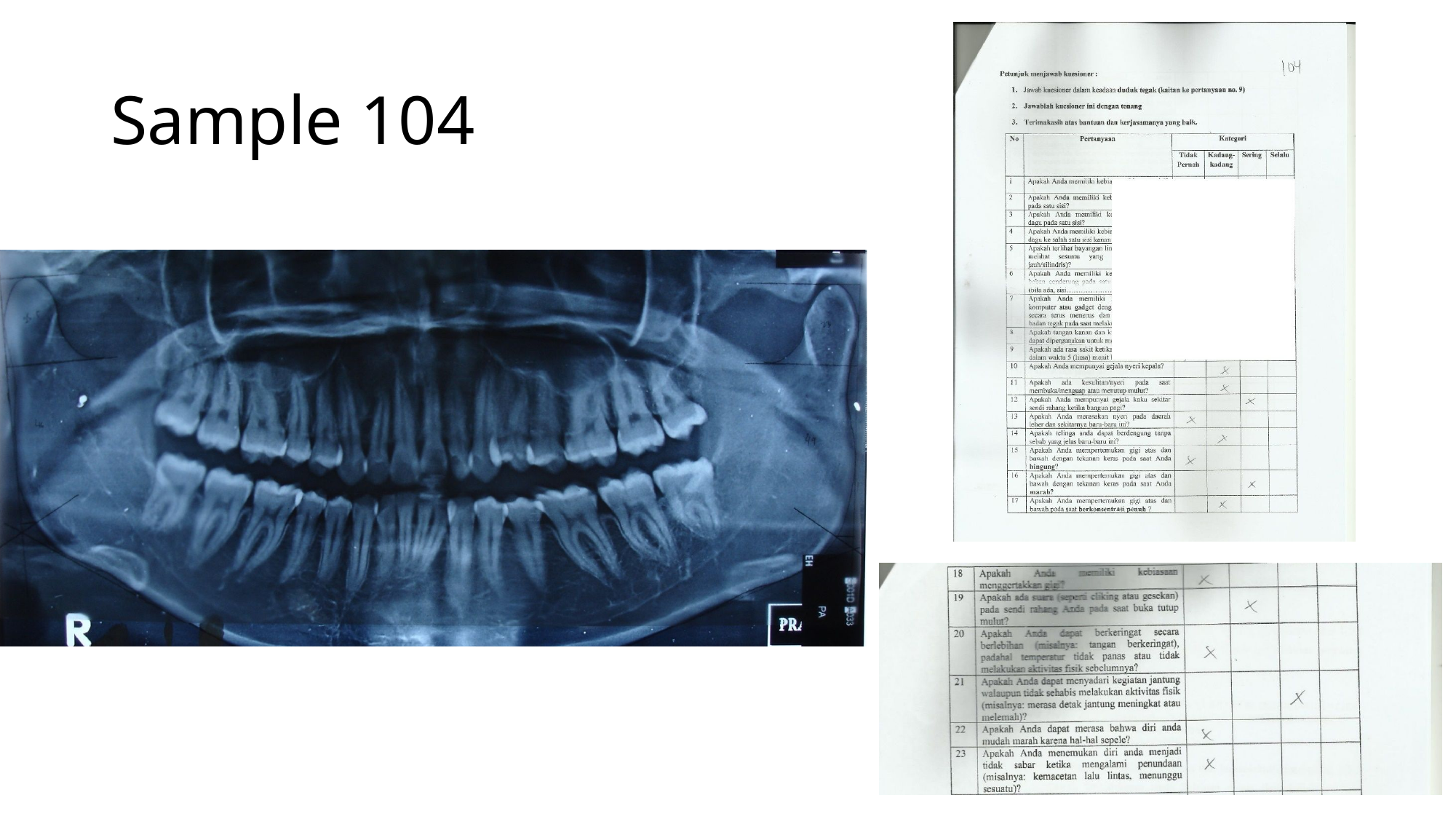

# Sample 104

## Slide 82
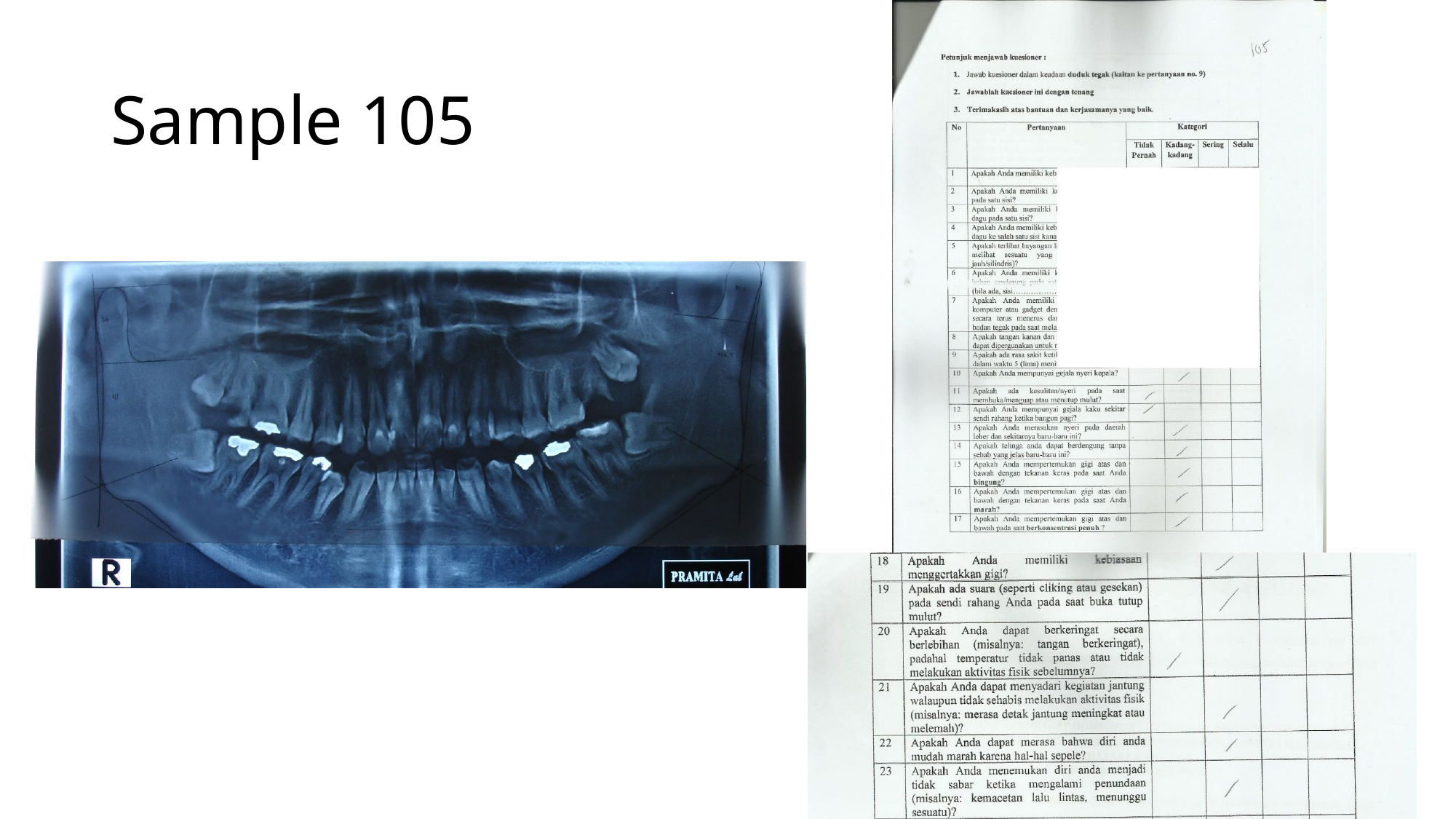

# Sample 105

## Slide 83
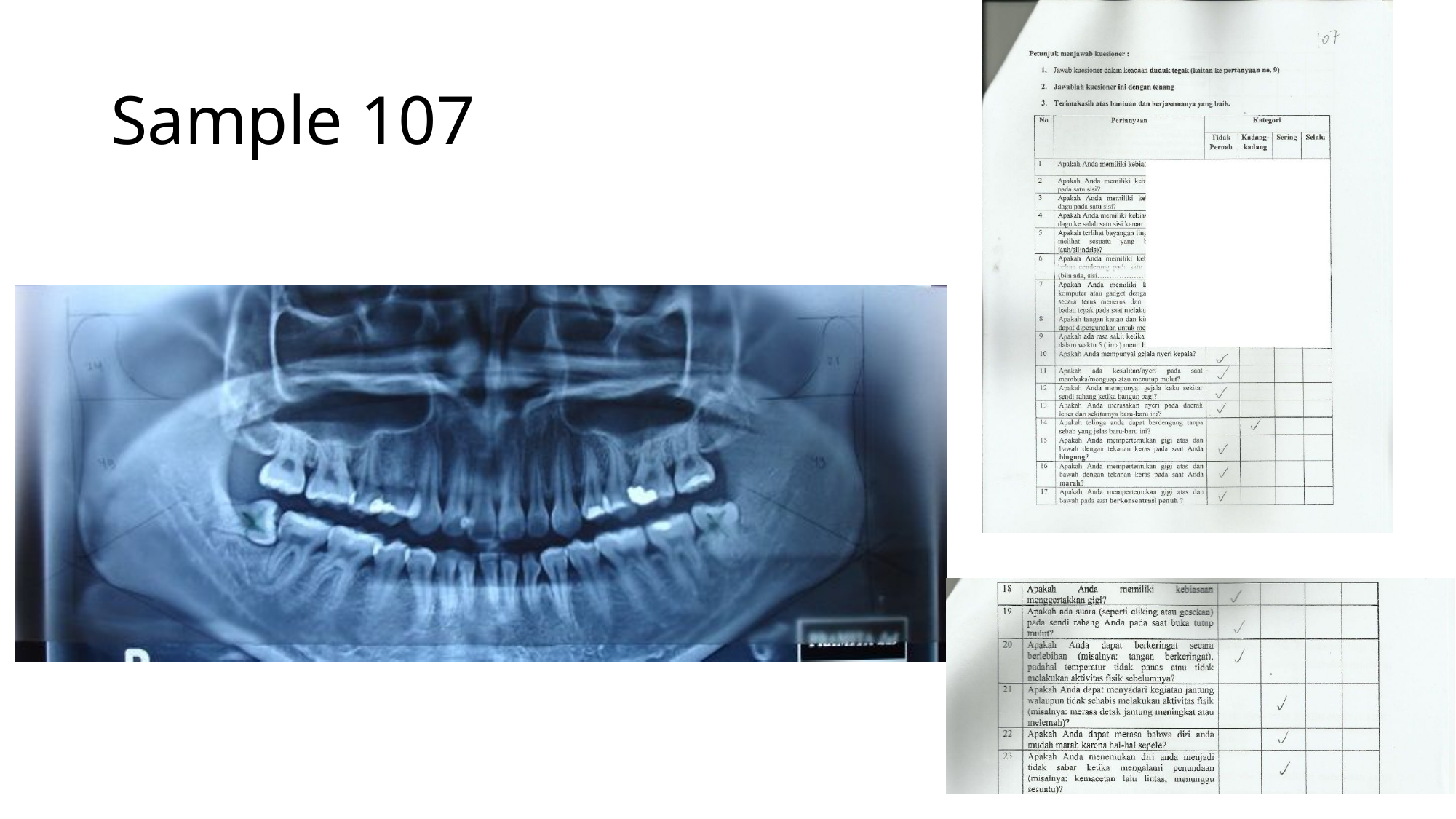

# Sample 107

## Slide 84
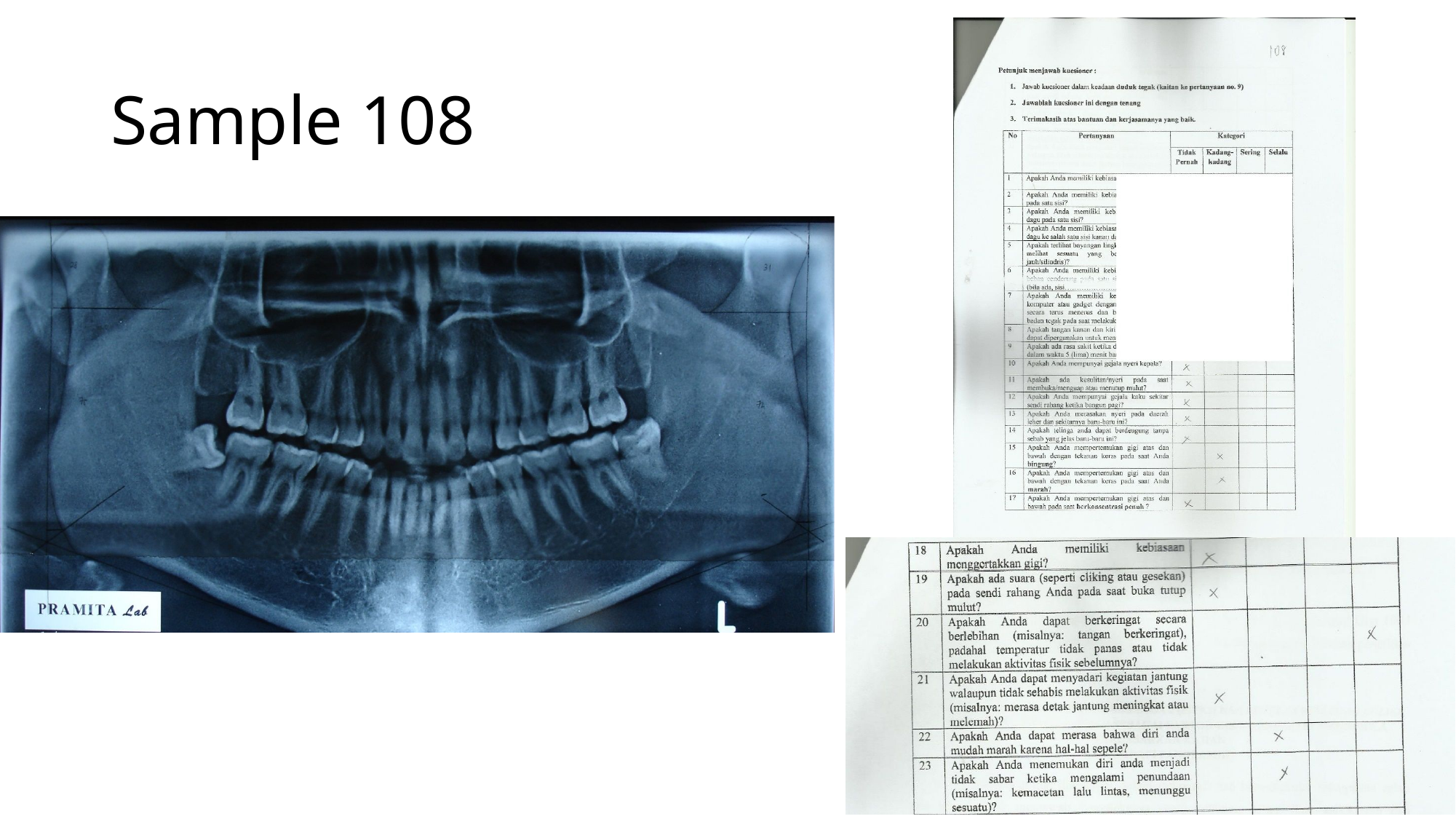

# Sample 108

## Slide 85
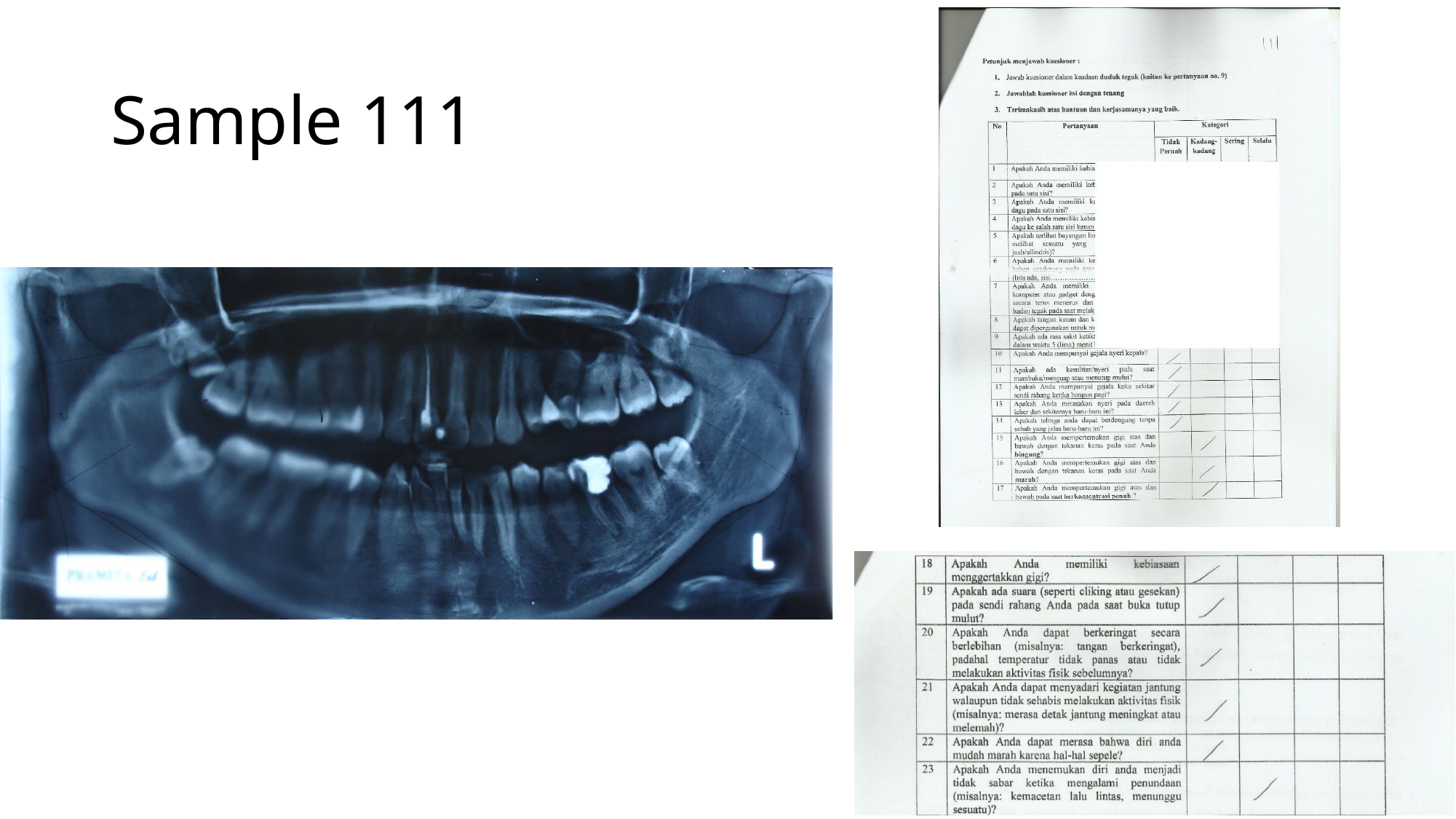

# Sample 111

## Slide 86
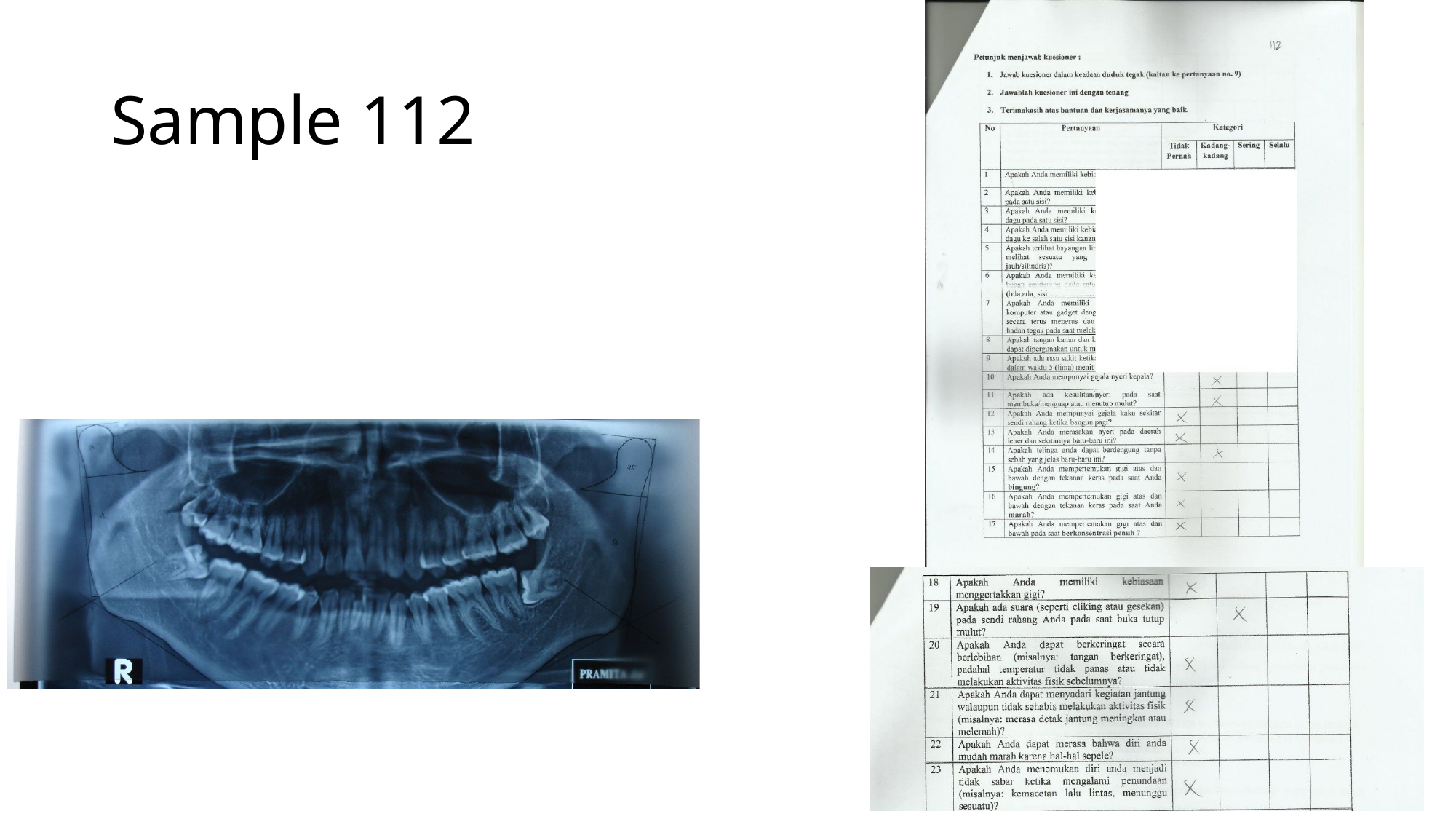

# Sample 112

## Slide 87
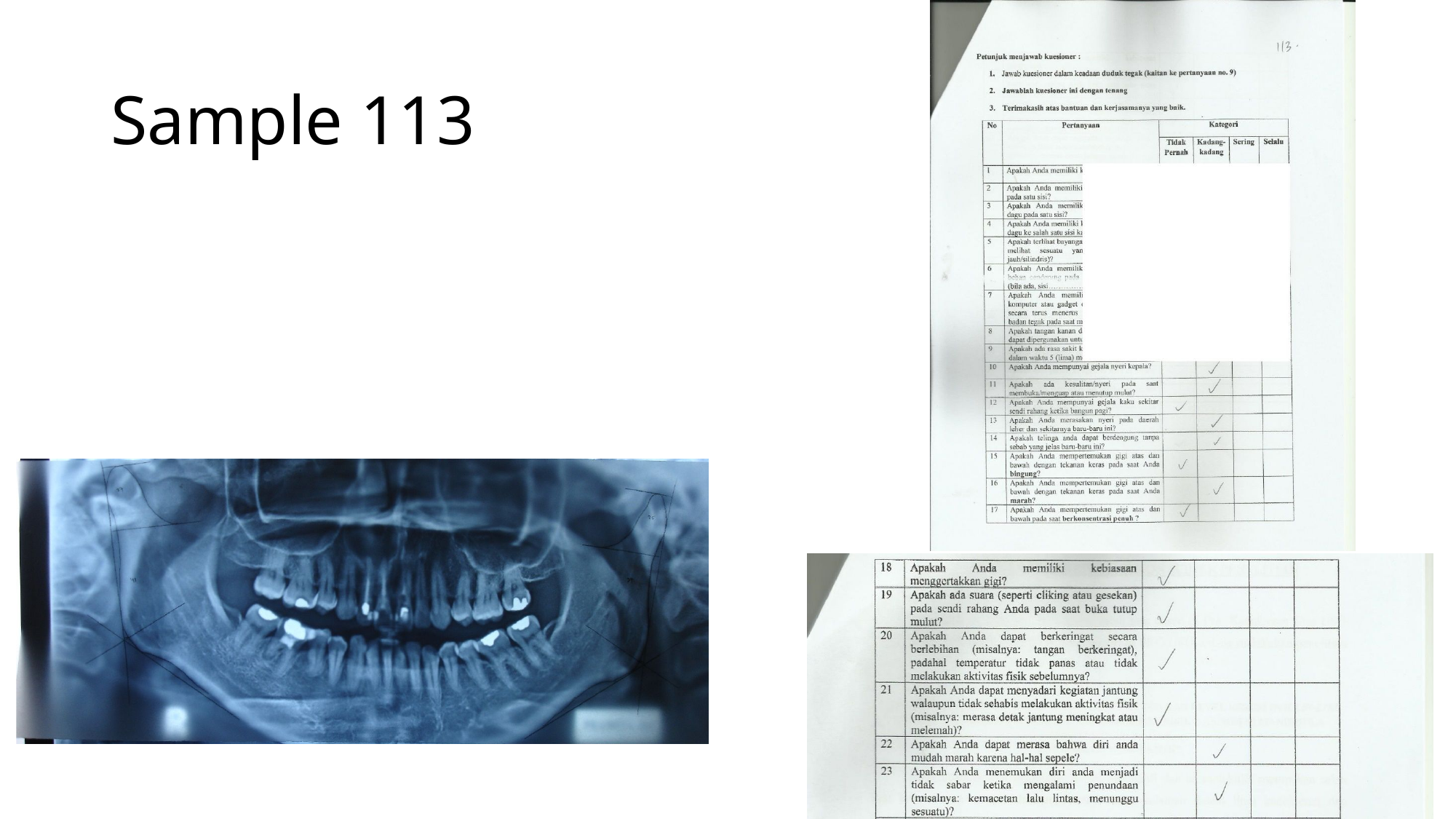

# Sample 113

## Slide 88
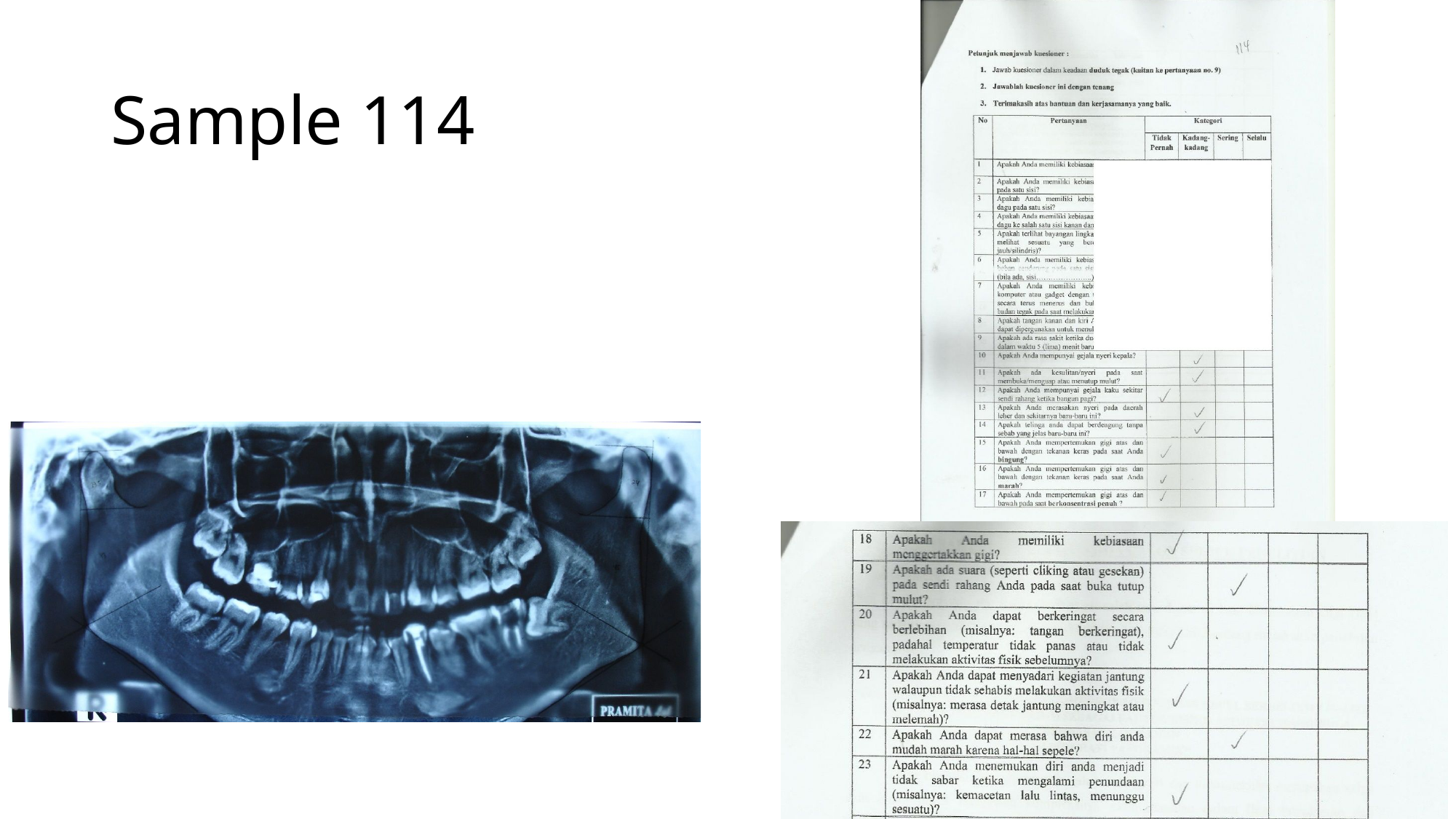

# Sample 114

## Slide 89
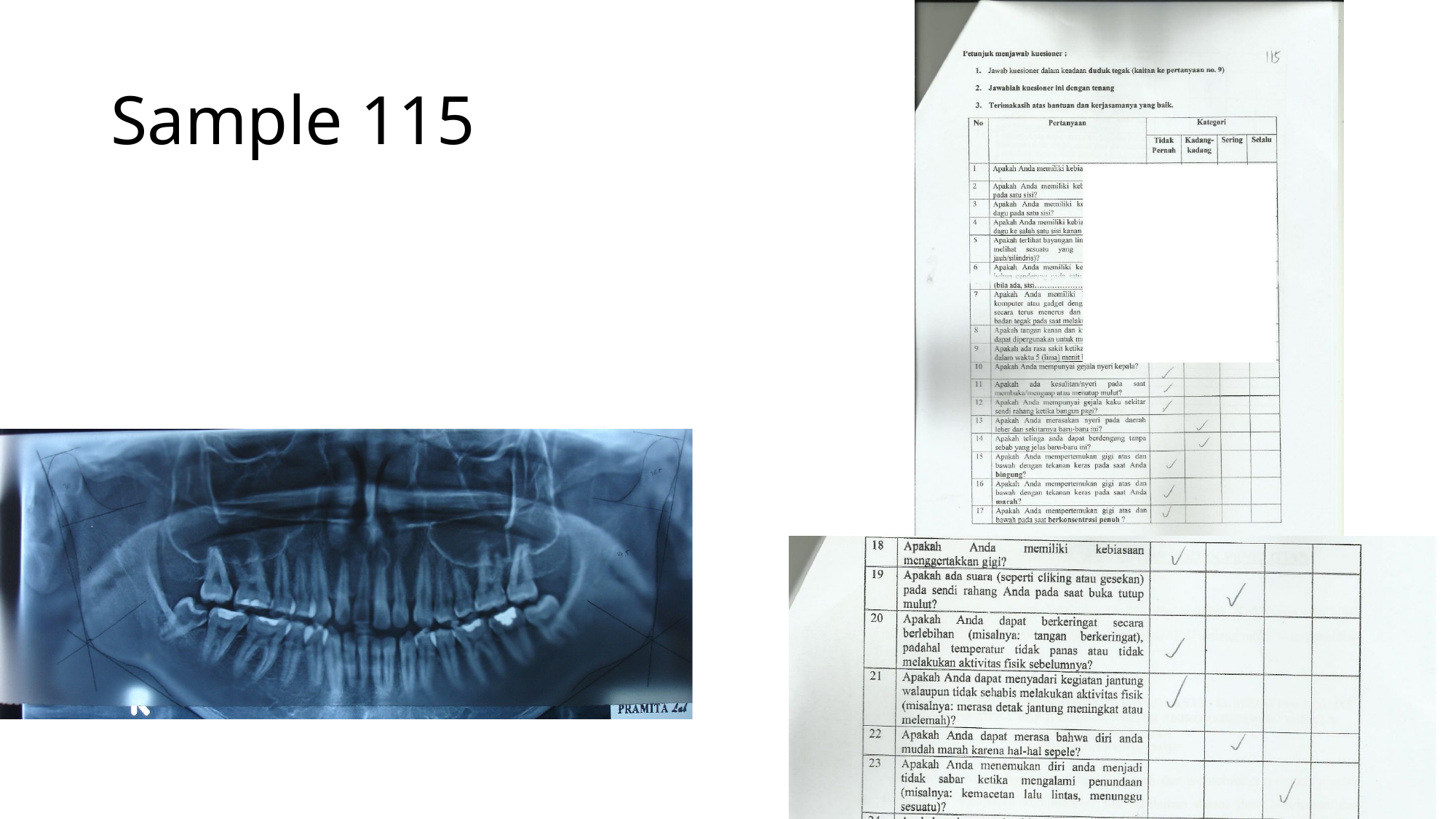

# Sample 115

## Slide 90
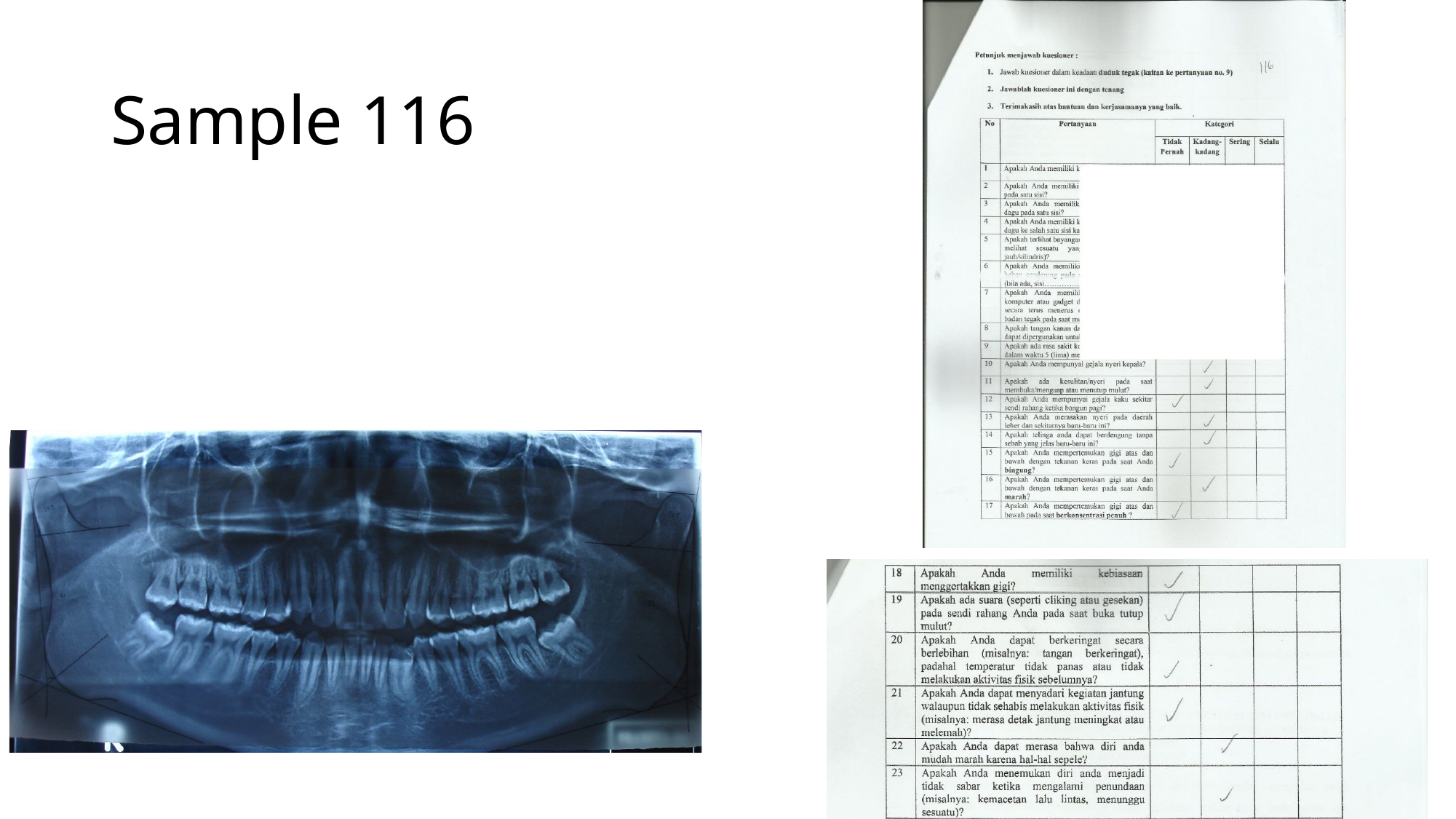

# Sample 116

## Slide 91
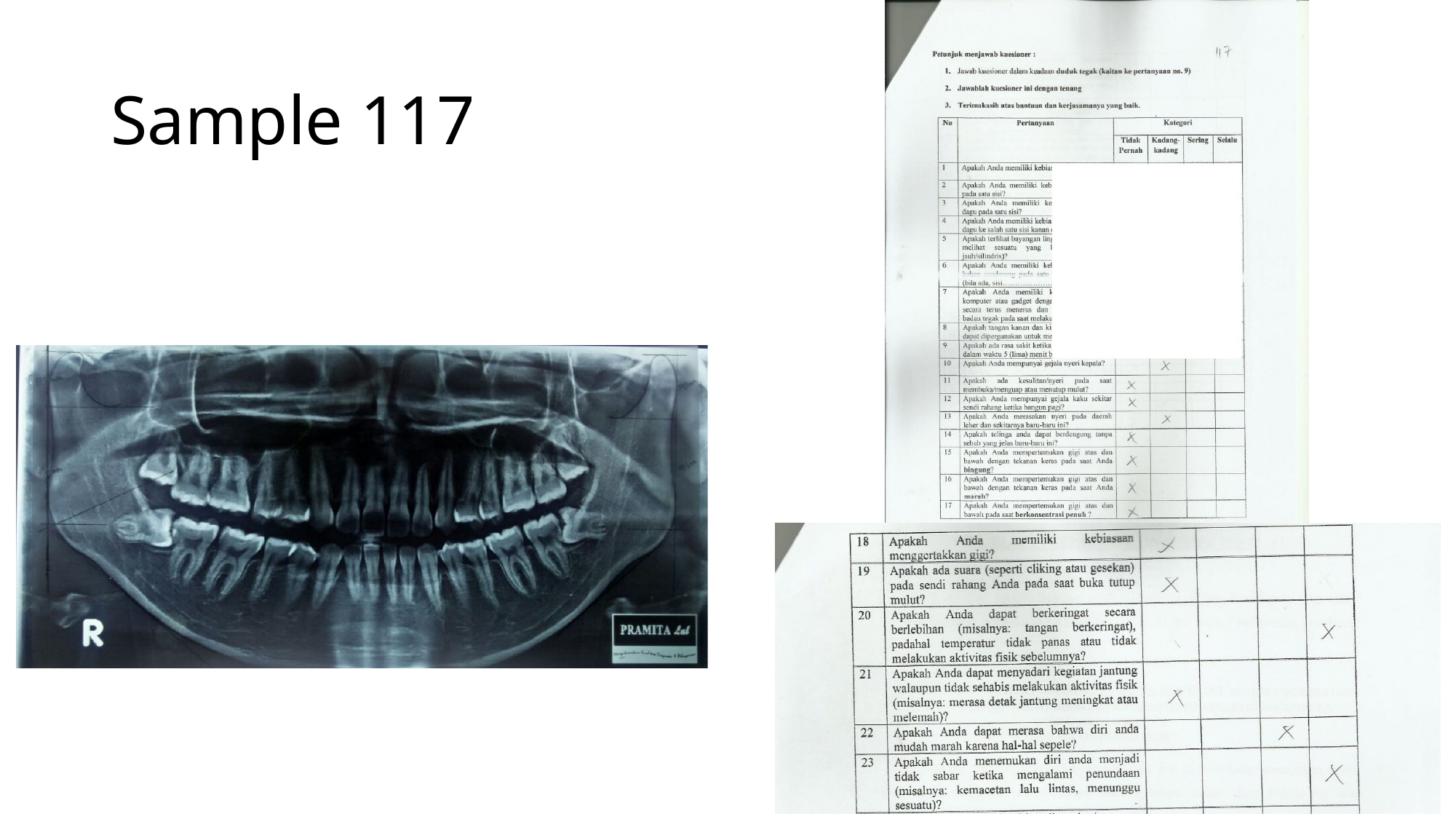

# Sample 117

## Slide 92
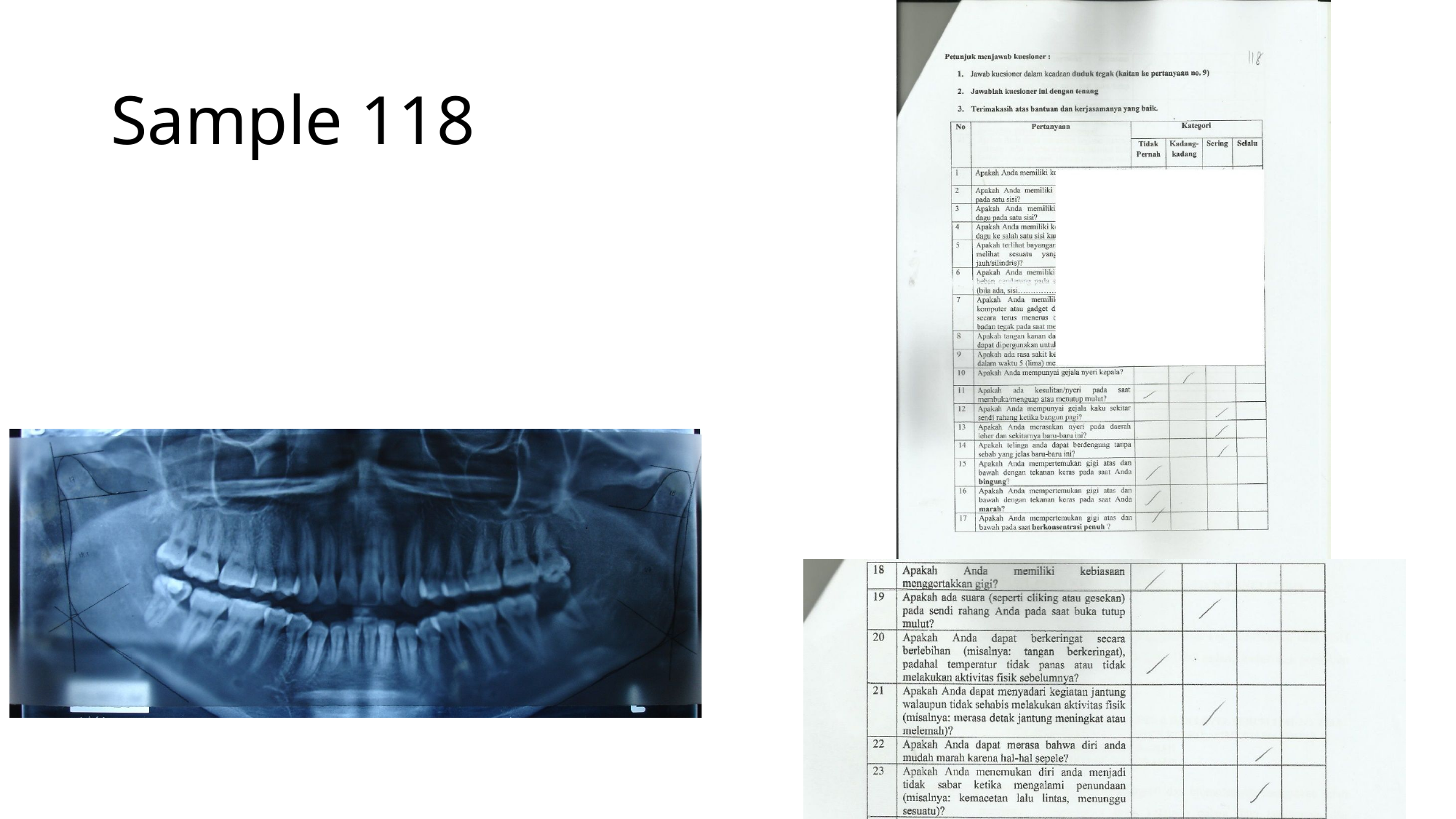

# Sample 118

## Slide 93
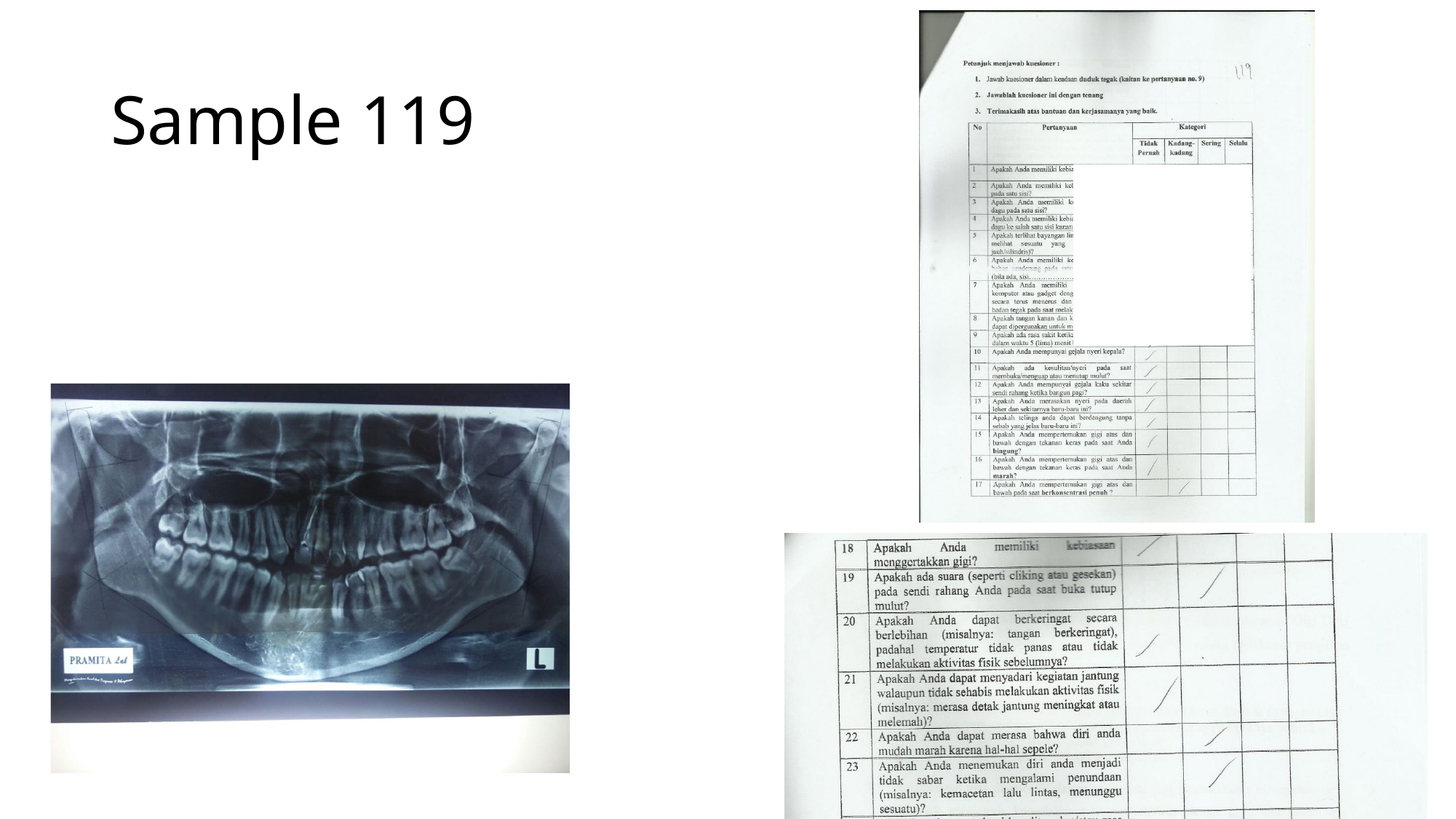

# Sample 119

## Slide 94
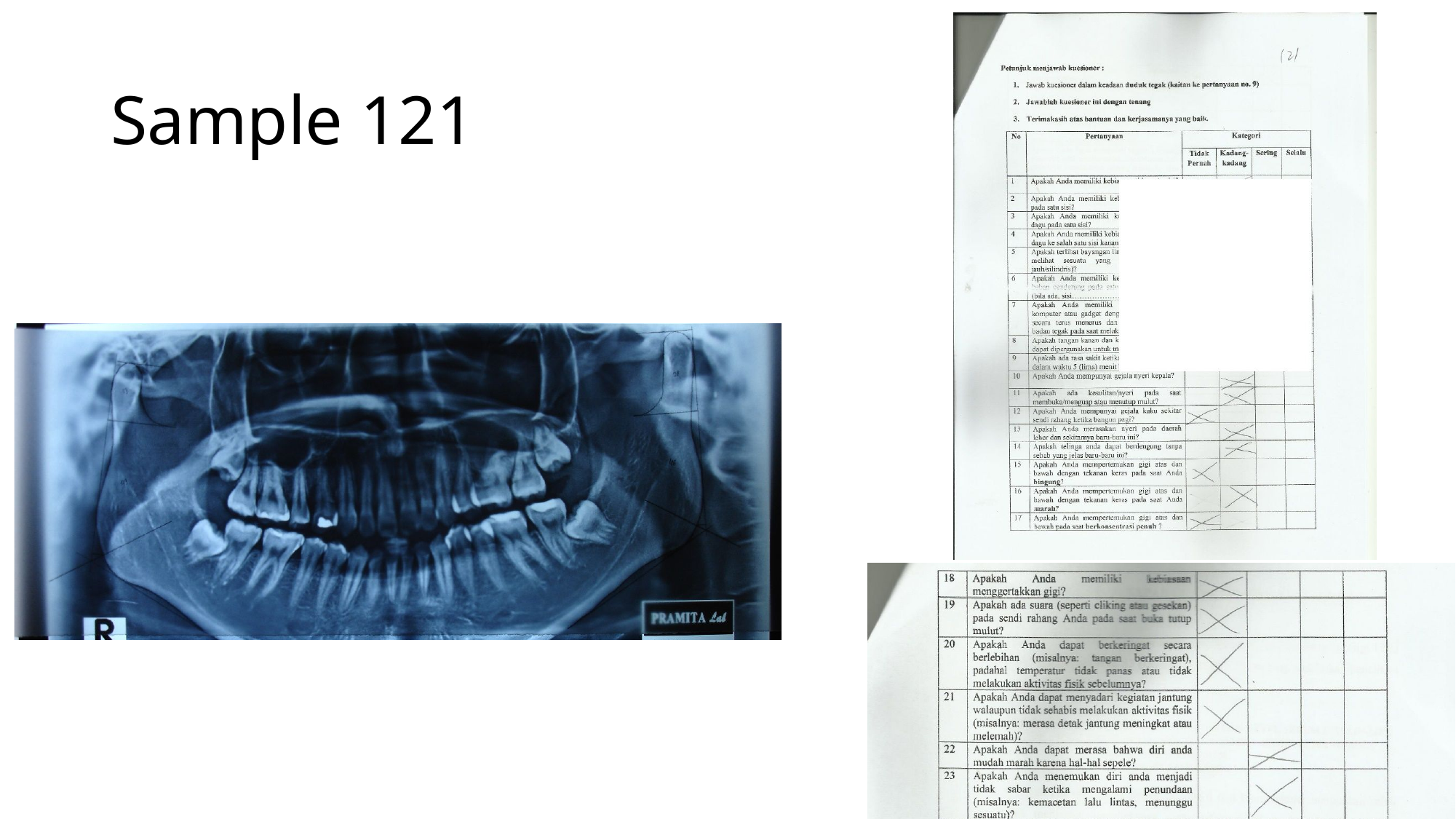

# Sample 121

## Slide 95
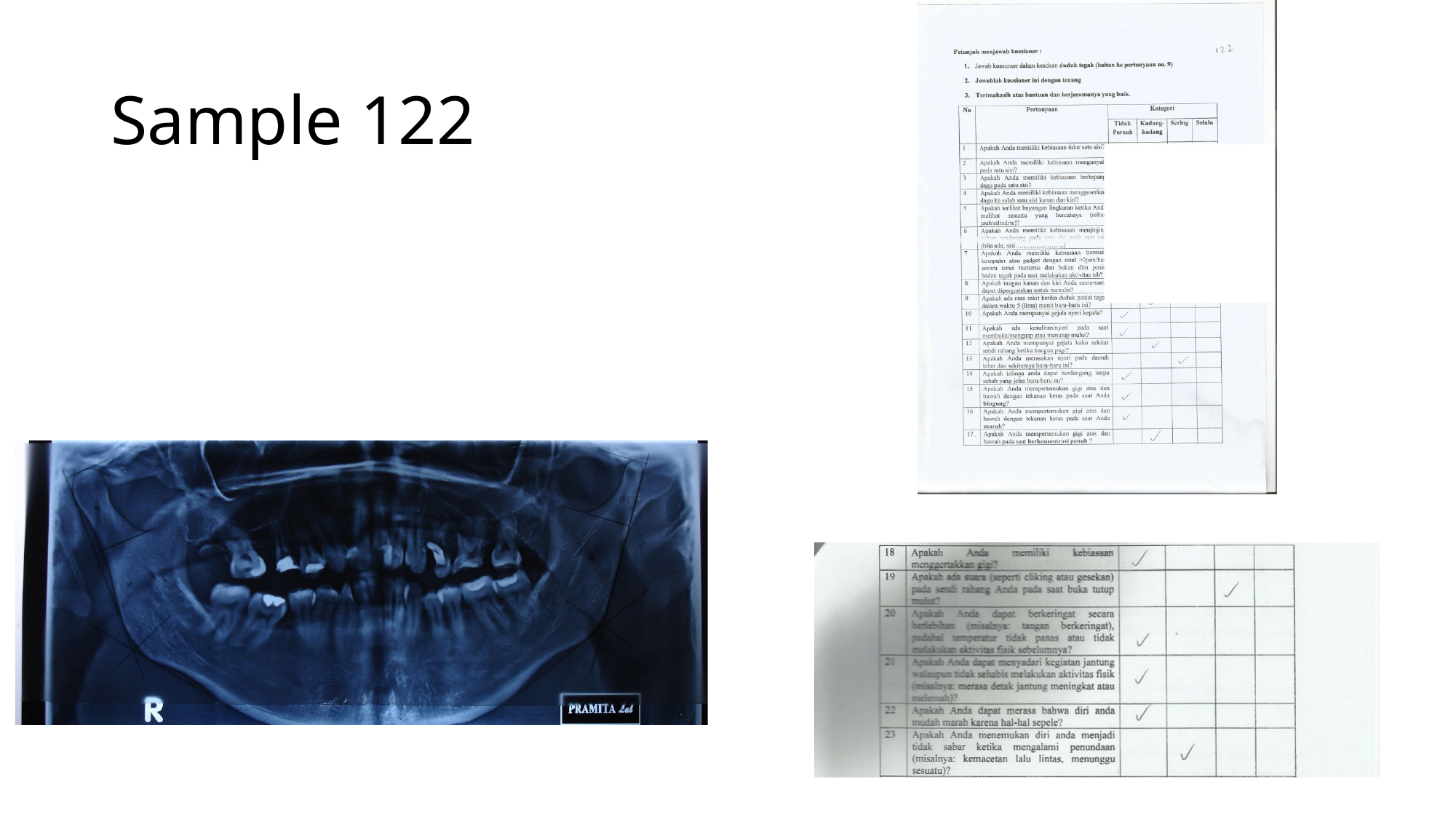

# Sample 122

## Slide 96
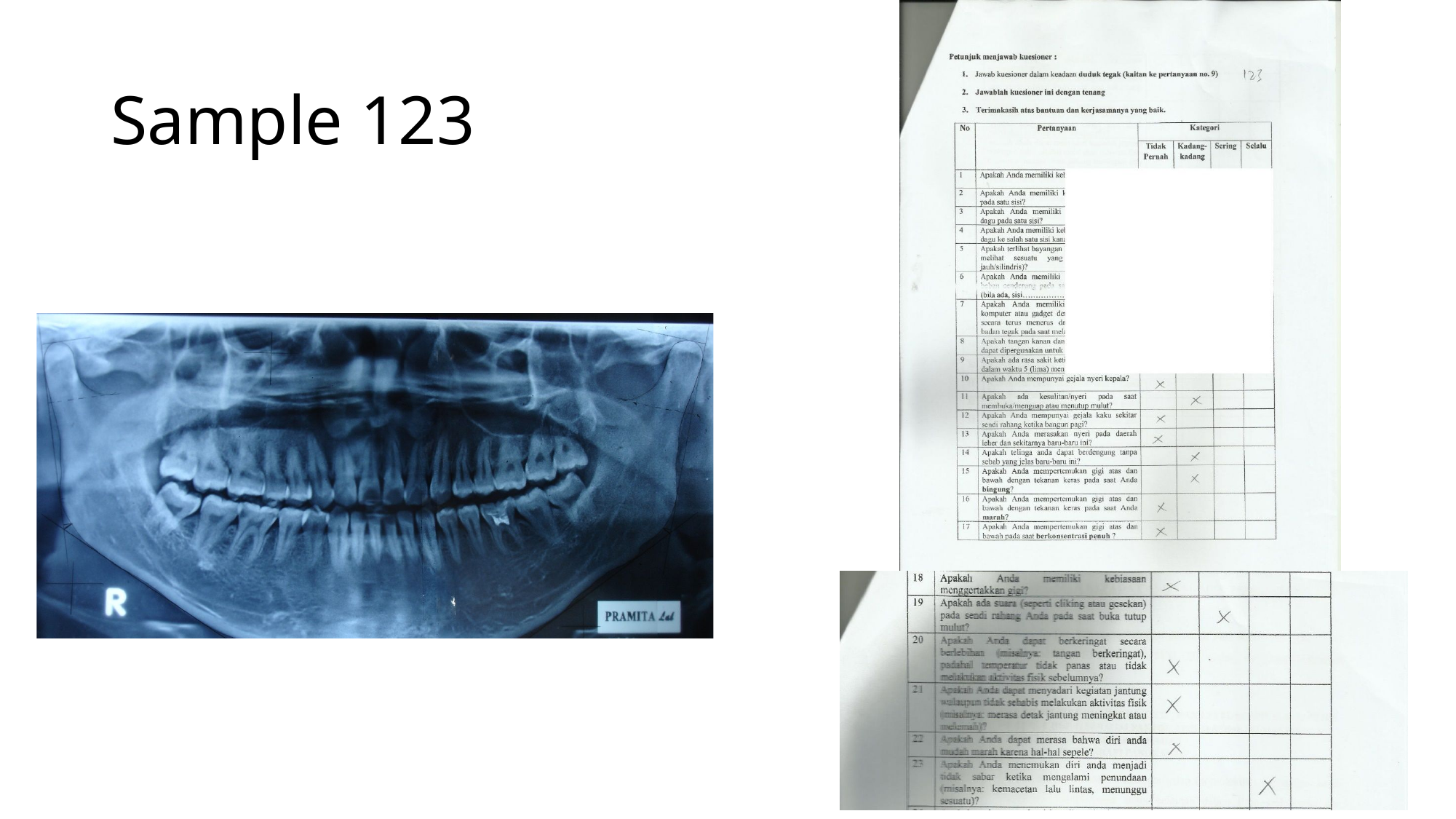

# Sample 123

## Slide 97
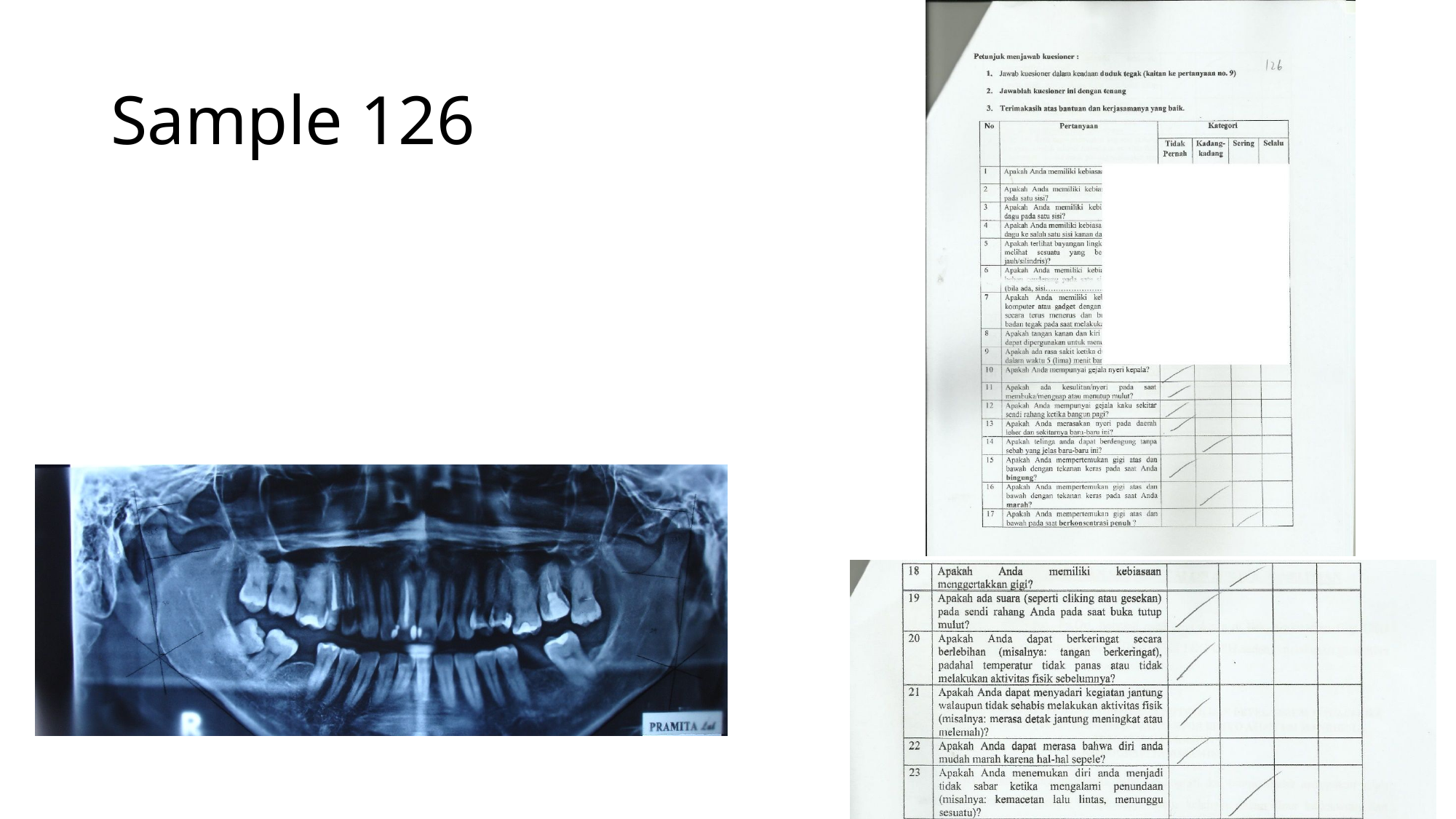

# Sample 126

## Slide 98
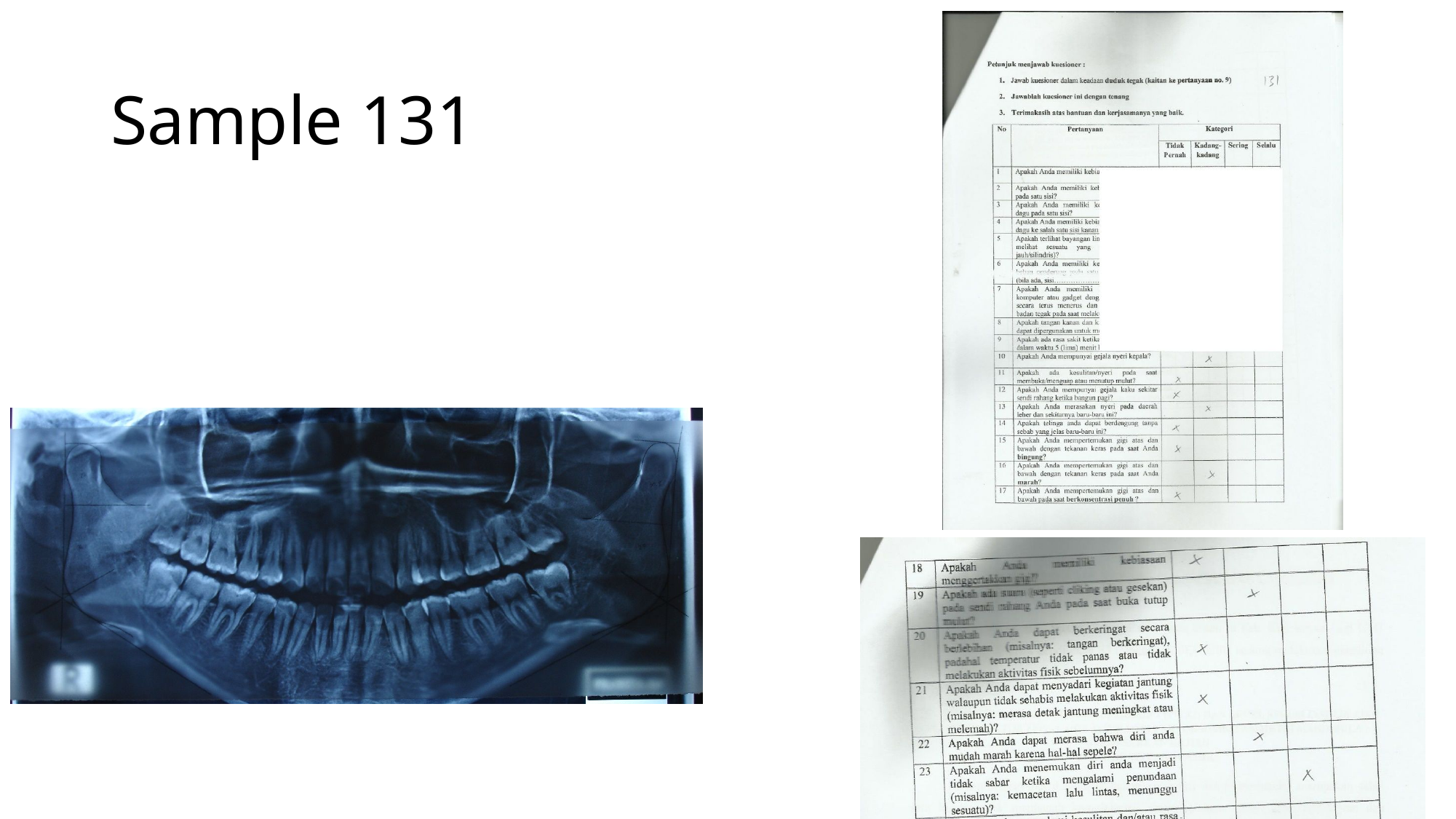

# Sample 131

## Slide 99
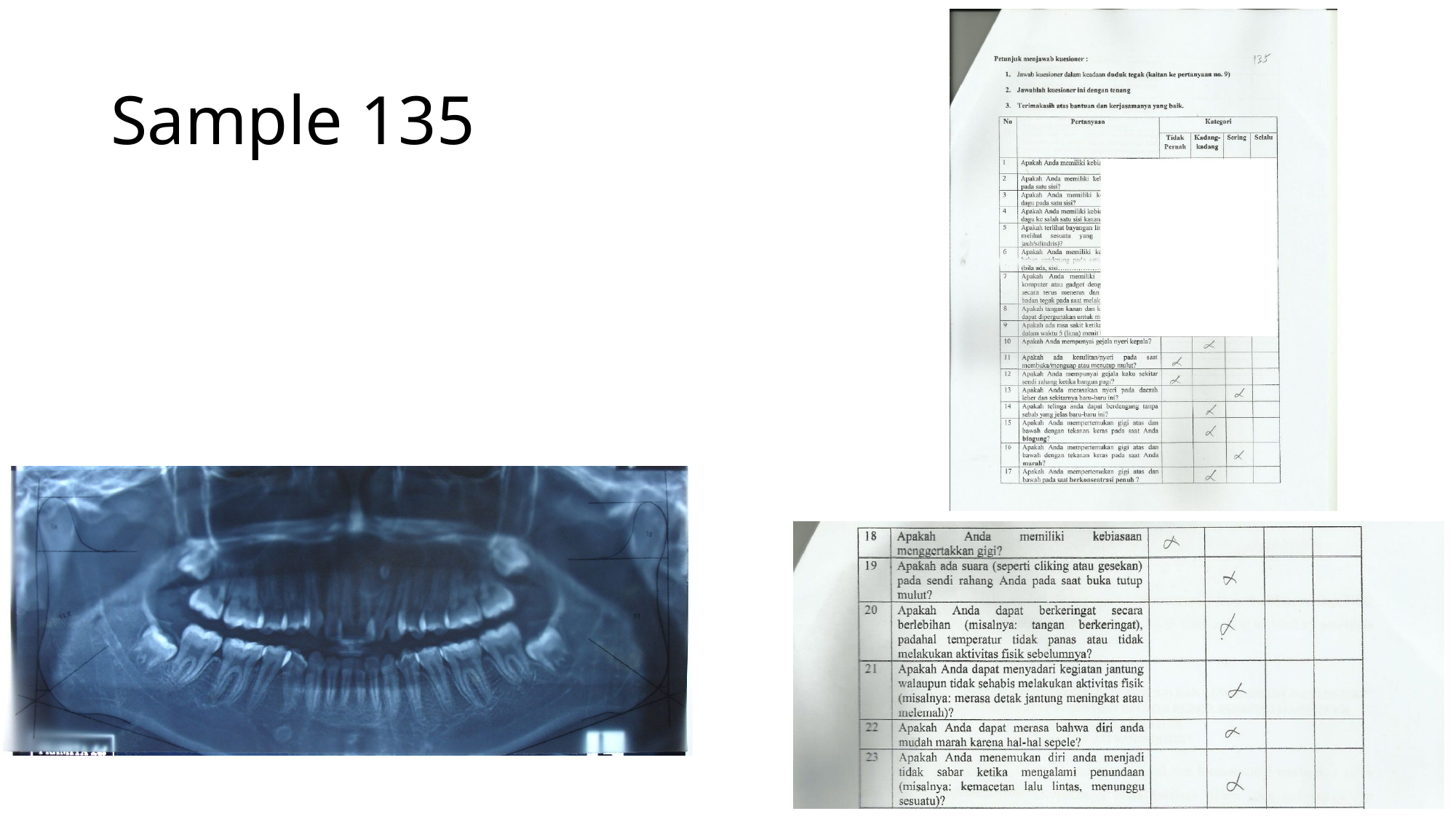

# Sample 135

## Slide 100
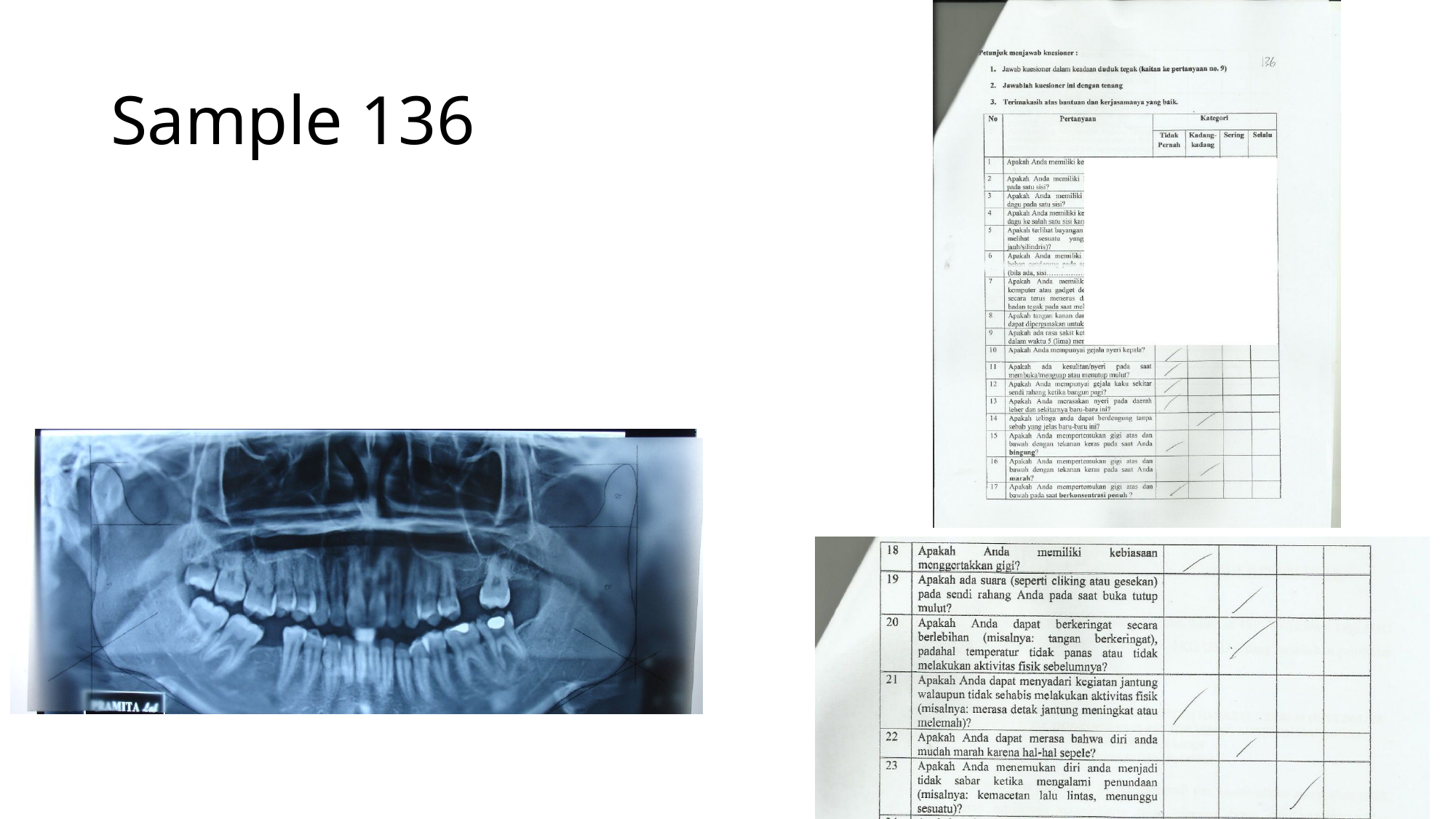

# Sample 136

## Slide 101
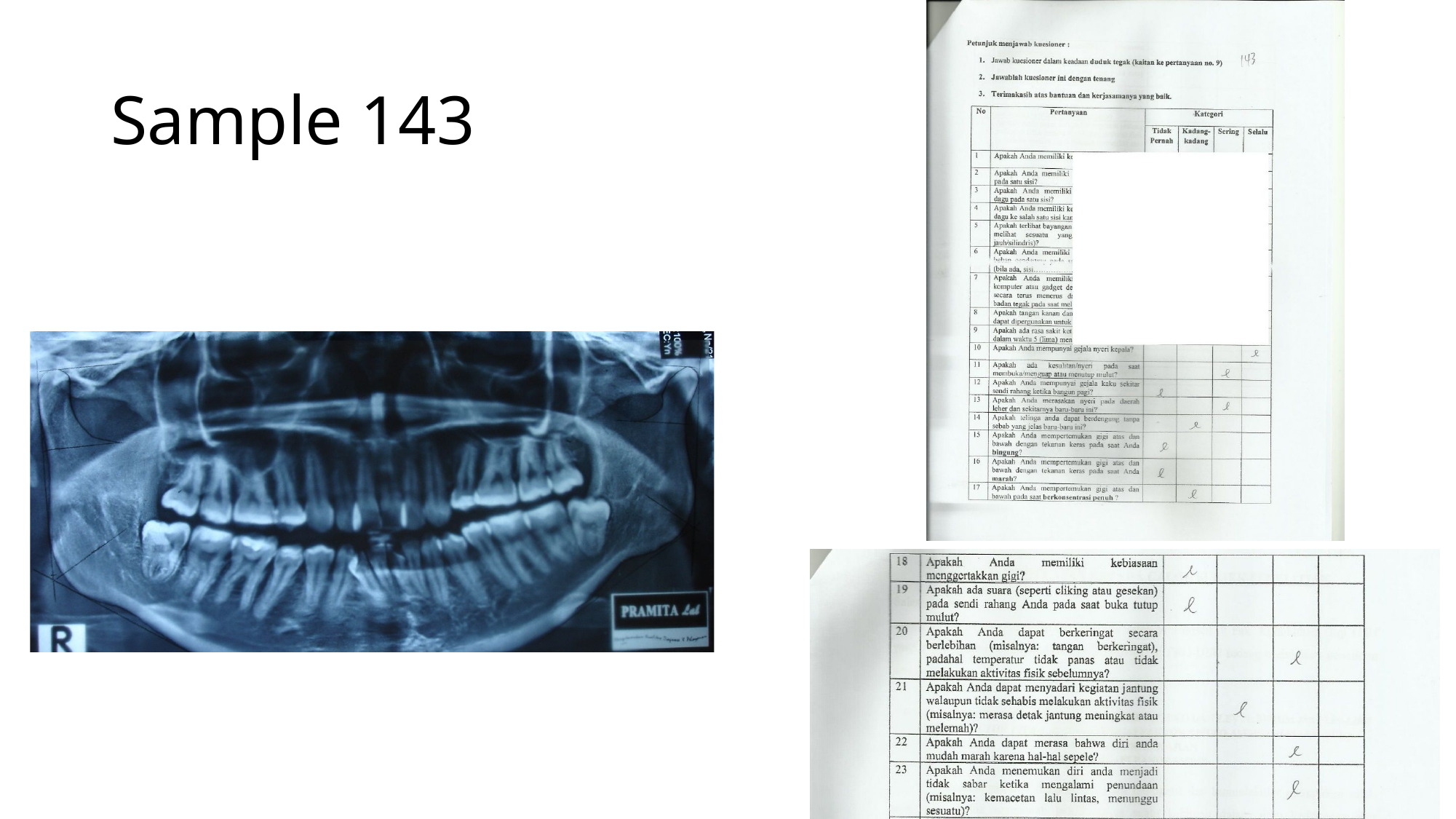

# Sample 143

## Slide 102
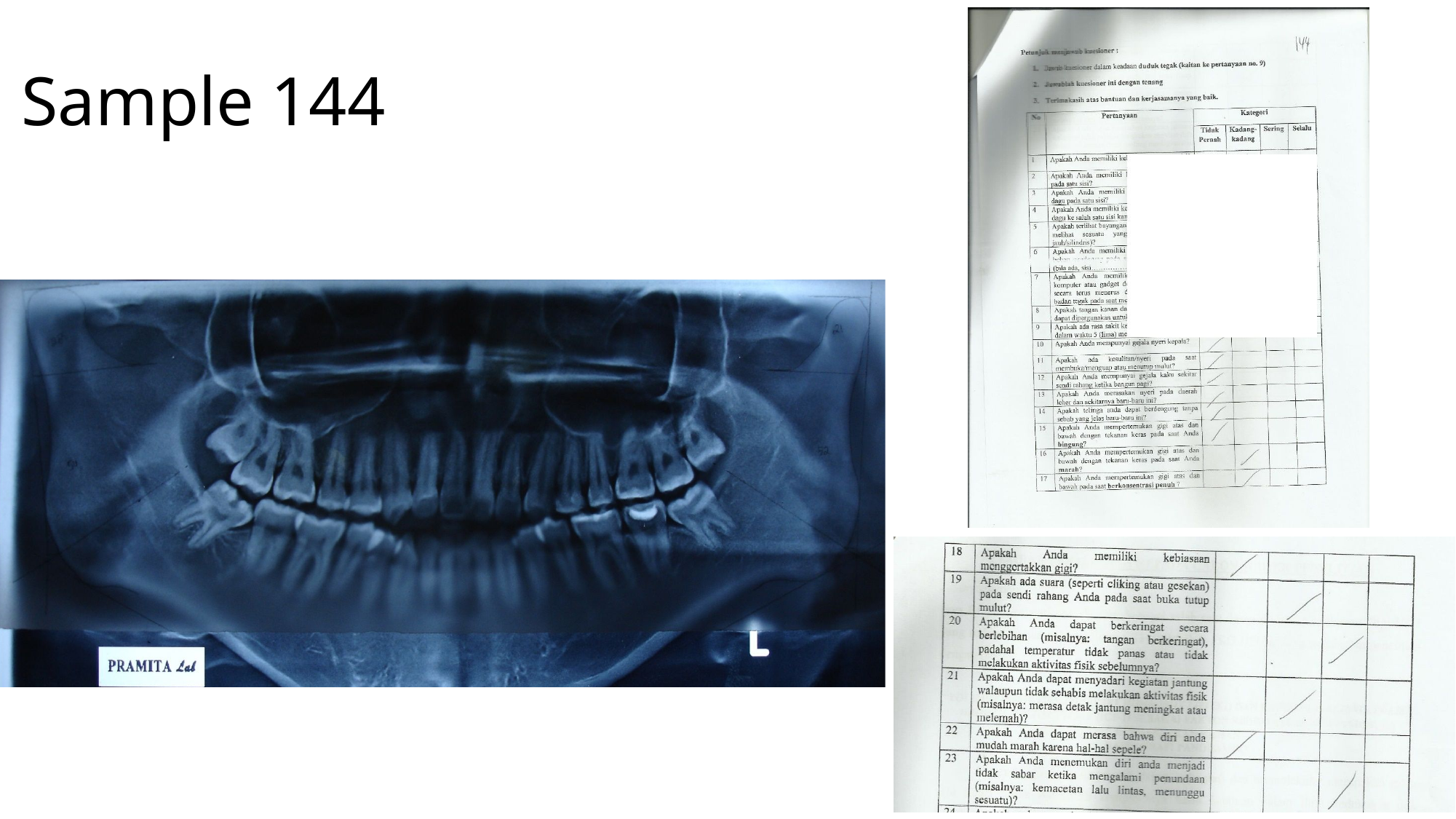

# Sample 144

## Slide 103
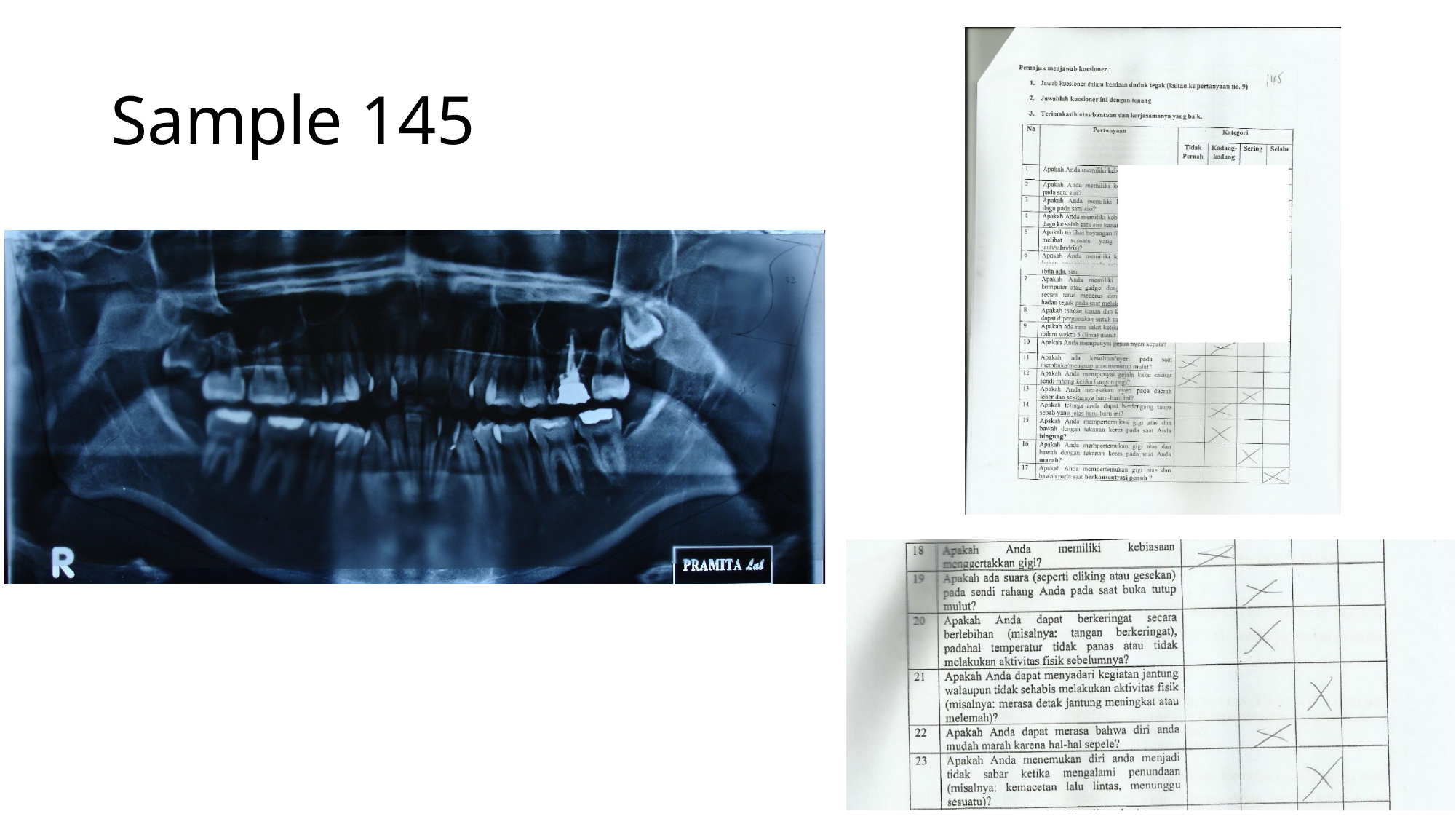

# Sample 145

## Slide 104
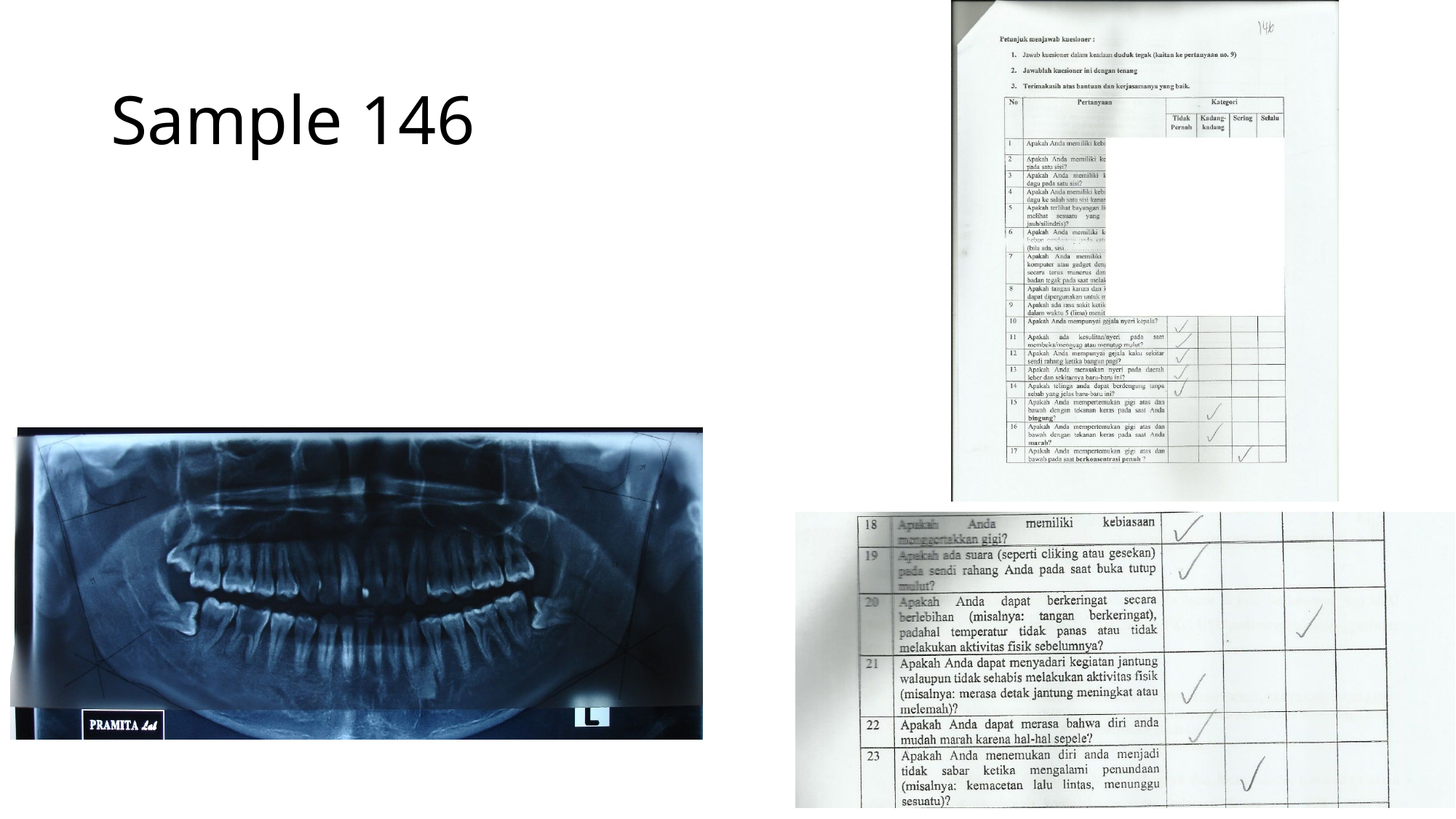

# Sample 146

## Slide 105
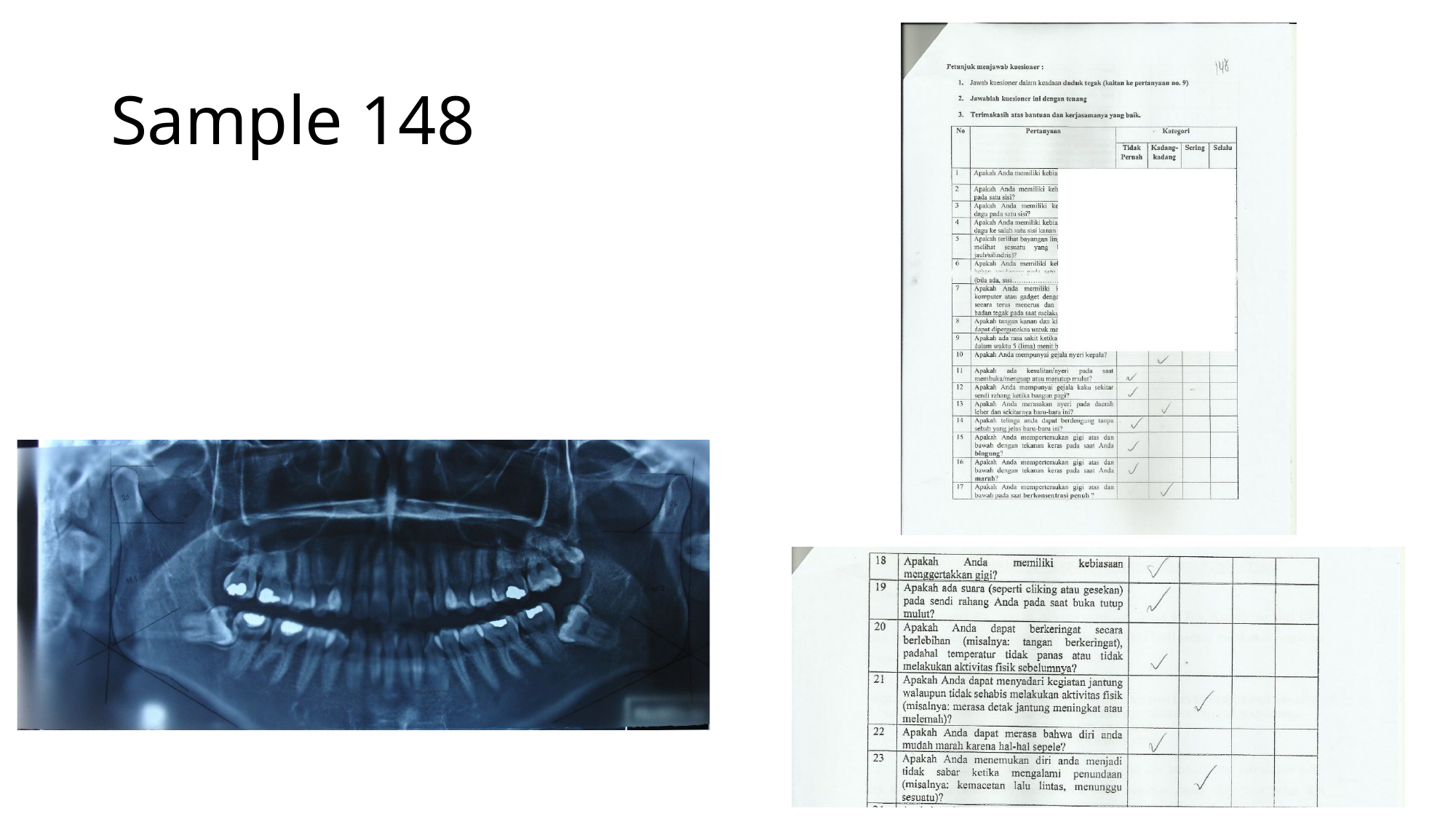

# Sample 148

## Slide 106
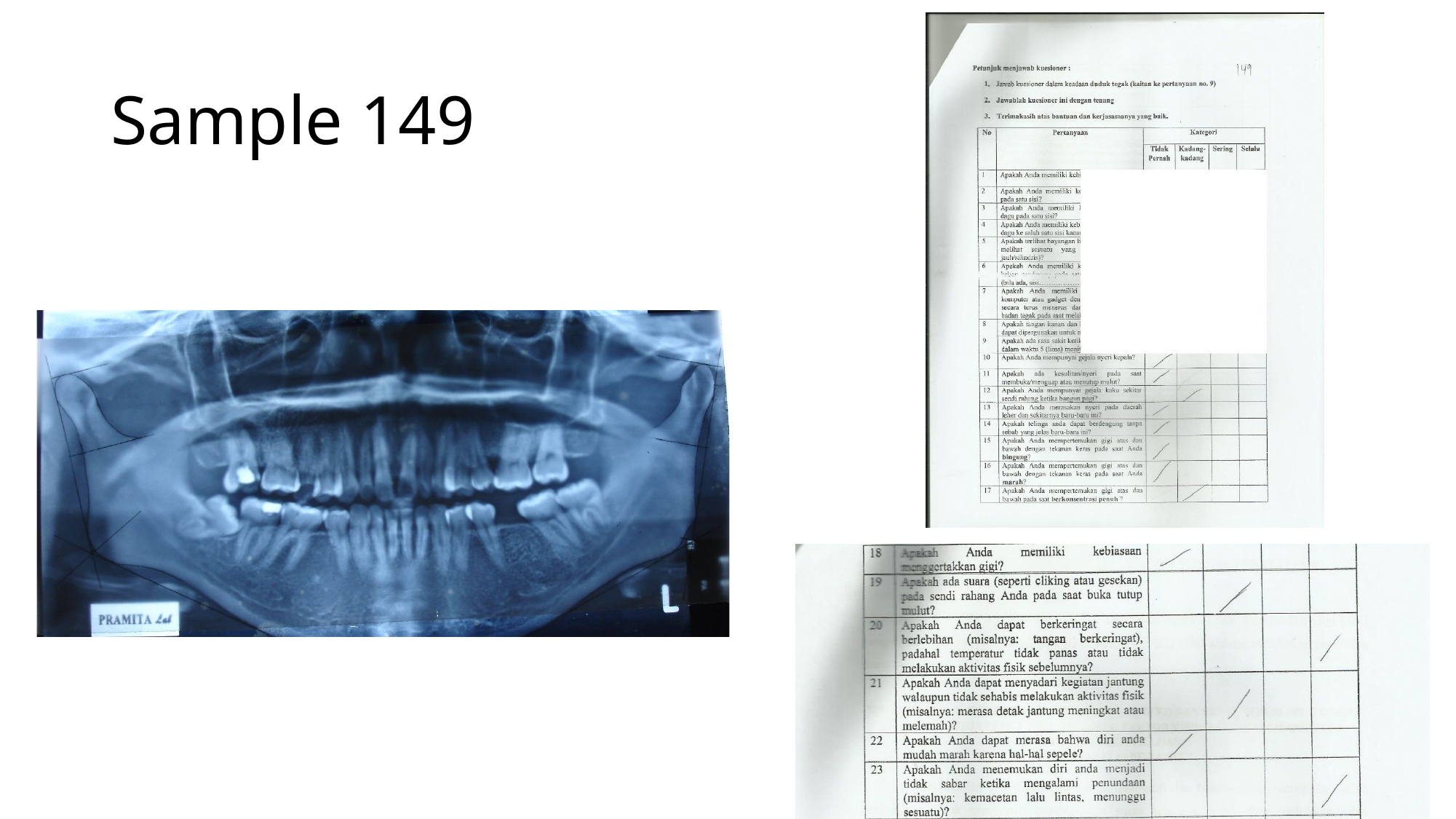

# Sample 149
