# Supplementary material for: TMD symptoms and vertical mandibular symmetry in young adult orthodontic patients in North Sumatra, Indonesia: a cross-sectional study
Source: F1000Res. 2018 Jul 16;7:697. Originally published 2018 Jun 4. [Version 2] doi: 10.12688/f1000research.14522.2 (PMC5998004; doi:10.12688/f1000research.14522.2)
Supplement: Peer review history

# Peer review: Referee response for version 1

**DOI:** 10.5256/f1000research.15809.r34686

**Published:** 2018-06-18

**Authors:** Ashok Karad

**Affiliations:** 1 Smile Care, Mumbai, Maharashtra, India

Temporomandibular disorders (TMD) in many patients constitute one of the most frequent causes of non-dental pain in the orofacial region. It is universally accepted that the etiology of TMD is multifactorial, including the structural factors such as mandibular asymmetry. However, a mild degree of asymmetry in the craniofacial region is common in humans, including in individuals with a normal facial appearance. These patients often present with complain of impaired facial appearance and occlusal malfunction, which underlines the importance of this subject. The literature shows that the Temporomandibular joint internal derangement is also associated with mandibular asymmetry. Nevertheless, how skeletal discrepancies in mandibular asymmetry relate to TMJ disorders is still debatable. 

 This article by Ervina Sofyanti et al is a great step towards evaluating the association between TMD symptoms and vertical mandibular asymmetry in 106 young adult orthodontic patients in North Sumatra, Indonesia. The results of this study show that there is no significant correlation between vertical mandibular asymmetry and TMD symptoms. 

 For better clarity and understanding, especially for the readers who are new to this subject, it would be appropriate if the authors could provide more details on Kjellberg's technique in the 'Methods' section. To enhance it further, it should include the exclusion criteria as well.

 Modern dental practice is focused on individualized diagnosis and treatment planning. Considering extreme variations in morphological and functional characteristics of an individual patient, it would be interesting to design a study involving these factors to fine-tune the results. This is because presence or absence of functional disturbances determines the discrepancy at the joint level in sagittal, vertical and transverse planes.

 The authors have done well in this interesting research work.

I have read this submission. I believe that I have an appropriate level of expertise to confirm that it is of an acceptable scientific standard.

# Response

**Published:** 2018-06-23

**Authors:** Ervina Sofyanti

**Affiliations:** Department of Orthodontics, Faculty of Dentistry, Indonesia

Dear Dr. Karad,

 Thank you very much for your kind assistance in reviewing our manuscript and valuable insight for our future work. As Dr. Narmada's comment also about the *Kjellberg's *technique, we will revise and update the material and method section.

# Peer review: Referee response for version 1

**DOI:** 10.5256/f1000research.15809.r34685

**Published:** 2018-06-12

**Authors:** Farhad B. Naini, Ashraf Messiha

**Affiliations:** 1 St George’s NHS Foundation Trust, London, UK; 2 St George’s Hospital & Medical School, London, UK

The article by Sofyanti et al. regarding the potential correlation between symptoms of temporomandibular joint dysfunction/disorder (TMD) and vertical mandibular symmetry is timely. 106 young adults referred for orthodontic treatment were questioned formally about TMD symptoms, and their responses correlated with objective evaluation of their orthopantomographs (OPTs). Of the 63 patients confirmed as having some degree of vertical mandibular asymmetry, and presumably requiring orthodontic treatment, 39 presented with symptoms of TMD, and 24 did not have any symptoms of TMD. The conclusion of the study was that there does not appear to be a significant association between vertical mandibular asymmetry and TMD symptoms.

 The conclusion of this investigation is not surprising. All else being equal, mandibular asymmetries without functional problems would logically not lead to any greater likelihood of TMD symptoms than the rest of the population. Though TMD may be multifactorial in origin, psychosocial stress appears to be a common thread. Patients with excellent dental occlusions may develop TMD, particularly during stressful periods in their lives, whereas many patients with quite complex malocclusions may never develop TMD. Nevertheless, malocclusions linked to parafunctional activities or leading to functional occlusal problems may demonstrate a higher preponderance to TMD. As such, it would be very interesting to repeat this study looking at the effects of mandibular lateral displacements (functional shifts) and any potential correlations with TMD. True skeletal asymmetries without lateral mandibular displacements are less likely to lead to TMD, but lateral displacements and associated functional problems may be of greater concern. Additionally, It would be potentially interesting to evaluate the prevalence of TMD comparing patients with predominantly vertical mandibular asymmetries (hemimandibular hyperplasia) and those with predominantly horizontal mandibular asymmetries (hemimandibular elongation).

 Another interesting question is the correlation of the variety of TMD symptoms in relation to specific malocclusions, whether dental or skeletal. Different TMD symptoms point to different aetiological factors. For example, with TM joint noises, clicking tends to be a sign of disc displacement/internal derangement, whereas crepitus may be due to arthritic-type changes. In the same way, myogenic pain, or trismus, may have diverse aetiology. It is worth exploring the potential links of dentoskeletal malocclusions not only to TMD as an all-encompassing diagnosis, but to the different symptoms comprising the diagnosis of TMD.

 It should also be noted that there is improved clinical awareness, and enhanced imaging availability, with t1 or t2-weighted magnetic resonance imaging (MRI) used to establish more accurately the aetiology and nature of a dysfunctioning joint. Therefore, in cases where asymmetry directly results from previous trauma, or with developmental, congenital, and hypermobility disorders, it is disc position that seems to be the determining factor in the overall stability of the mechanics of the joint. Disruption, or disc displacement, leads to various degrees of arthrogenic and neurogenic symptoms of TMD, as opposed to myogenic TMD that seems to be a common symptom amongst the anxious and stressed TMD sufferer.

 The authors should be congratulated on a thought-provoking article.

We have read this submission. We believe that we have an appropriate level of expertise to confirm that it is of an acceptable scientific standard.

# Response

**Published:** 2018-06-23

**Authors:** Ervina Sofyanti

**Affiliations:** Department of Orthodontics, Faculty of Dentistry, Indonesia

Dear Dr Farhad and Dr. Messiha,

Thank you very much for your kind assistance in reviewing our manuscript and providing us with valuable advices for the manuscript itself as well as for our future work. To respond with the reviews that have been provided, please allow us to comment as follows: 

1. **“It would be very interesting to repeat this study looking at the effects of mandibular lateral displacements (functional shifts) and any potential correlations with TMD. True skeletal asymmetries without lateral mandibular displacements are less likely to lead to TMD, but lateral displacements and associated functional problems may be of greater concern”.**

Thank you very much for this valuable insight. The authors have noted the concern of the presence of lateral displacement to the occurrence of TMD for our future work. This was not included in this project as this was just an initial pilot project.

2. **"It would be potentially interesting to evaluate the prevalence of TMD comparing patients with predominantly vertical mandibular asymmetries (hemimandibular hyperplasia) and those with predominantly horizontal mandibular asymmetries (hemimandibular elongation)".**

We thank you again for this valuable insight and definitely will be considered in our future work.

3. **"Therefore, in cases where asymmetry directly results from previous trauma, or with developmental, congenital, and hypermobility disorders, it is disc position that seems to be the determining factor in the overall stability of the mechanics of the joint".**

In this study, the possible influence of any history of traumatic facial injury as well as congenital disease have been eliminated. And with regards to the position of the disc, this was not considered in this study but will surely be considered for our future work. The possible use of CBCT may perhaps assist our future work in determining disc position.

# Peer review: Referee response for version 1

**DOI:** 10.5256/f1000research.15809.r34688

**Published:** 2018-06-11

**Authors:** Ida Bagus Narmada

**Affiliations:** 1 Faculty of Dental Medicine, Airlangga University, Surabaya, Indonesia

Overall your paper is very good and well done, but here are some minor suggestions:

- Could you please state more about the Kjellberg’s technique, it is not well described in the methods section of your paper?
- Could you please inform about the study design (descriptive or analytic study)?
- Could you please inform about the sampling method and how to determine the sample size?
- You only mention the inclusion criteria, what about the exclusion criteria? Please mention it.
- Please mention and inform who examined the patient? How to examine the patient? Please explain.
- How about the competency of the examiner? How many people? How to calibrate each examiner’s perception. Please mention also who is in charge for radiographic image interpretation?

I have read this submission. I believe that I have an appropriate level of expertise to confirm that it is of an acceptable scientific standard.

# Response

**Published:** 2018-06-23

**Authors:** Ervina Sofyanti

**Affiliations:** Department of Orthodontics, Faculty of Dentistry, Indonesia

Dear Dr. Narmada, 

Thank you very much for your kind assistance in reviewing our manuscript. According to your some minor suggestions, we have revised within material and method section.

This is descriptive analytic study with cross-sectional approach and selection of sample with simple random sampling. In radiography analysis, we did inter-rater and intra-rater examiner.

TB : the consultant in dentomaxillofacial radiograph. ES did the radiographic image interpretation under supervised of TB. We traced the radiography manually according *Kjellberg's* technique as followed : CH is defined as the distance from CO (the highest point of the condylar head) to the mandibular notch (the deepest point between the coronoid process and the condylar process). RH is the distance from CO to the GO’ (the reflection of subdivision tangen from ramus and corpus mandibular to the ramus borderline). In order to obtain vertical mandibular symmetry based on the ratio of condylar and ramus height (Kjellberg’s technique).
